# Effective Targeting of Melanoma Cells by Combination of Mcl-1 and Bcl-2/Bcl-x_L_/Bcl-w Inhibitors

**DOI:** 10.3390/ijms25063453

**Published:** 2024-03-19

**Authors:** Zhe Peng, Bernhard Gillissen, Antje Richter, Tobias Sinnberg, Max S. Schlaak, Jürgen Eberle

**Affiliations:** 1 Skin Cancer Centre Charité, Department of Dermatology and Allergy, Charité—Universitätsmedizin Berlin, Charitéplatz 1, 10117 Berlin, Germany; zhe.peng@charite.de (Z.P.); tobias.sinnberg@charite.de (T.S.); max.schlaak@charite.de (M.S.S.); 2 Clinical Medicine, University of South China, Hengyang 421001, China; 3 Department of Hematology, Oncology, and Tumor Immunology, Charité—Universitätsmedizin Berlin, 13125 Berlin, Germany; bernhard.gillissen@charite.de (B.G.); antje.richter@charite.de (A.R.); 4 Division of Dermatooncology, Department of Dermatology, University of Tübingen, 72076 Tübingen, Germany

**Keywords:** melanoma, skin cancer, BH3 mimetics, therapeutic strategies, apoptosis induction, apoptotic pathways

## Abstract

Recent advances in melanoma therapy have significantly improved the prognosis of metastasized melanoma. However, large therapeutic gaps remain that need to be closed by new strategies. Antiapoptotic Bcl-2 proteins critically contribute to apoptosis deficiency and therapy resistance. They can be targeted by BH3 mimetics, small molecule antagonists that mimic the Bcl-2 homology domain 3 (BH3) of proapoptotic BH3-only proteins. By applying in vitro experiments, we aimed to obtain an overview of the possible suitability of BH3 mimetics for future melanoma therapy. Thus, we investigated the effects of ABT-737 and ABT-263, which target Bcl-2, Bcl-x_L_ and Bcl-w as well as the Bcl-2-selective ABT-199 and the Mcl-1-selective S63845, in a panel of four *BRAF*-mutated and *BRAF*-WT melanoma cell lines. None of the inhibitors showed significant effectiveness when used alone; however, combination of S63845 with each one of the three ABTs almost completely abolished melanoma cell survival and induced apoptosis in up to 50–90% of the cells. Special emphasis was placed here on the understanding of the downstream pathways involved, which may allow improved applications of these strategies. Thus, cell death induction was correlated with caspase activation, loss of mitochondrial membrane potential, phosphorylation of histone H2AX, and ROS production. Caspase dependency was demonstrated by a caspase inhibitor, which blocked all effects. Upregulation of Mcl-1, induced by S63845 itself, as reported previously, was blocked by the combinations. Indeed, Mcl-1, as well as XIAP (X-linked inhibitor of apoptosis), were strongly downregulated by combination treatments. These findings demonstrate that melanoma cells can be efficiently targeted by BH3 mimetics, but the right combinations have to be selected. The observed pronounced activation of apoptosis pathways demonstrates the decisive role of apoptosis in the loss of cell viability by BH3 mimetics.

## 1. Introduction

A strong increase in melanoma incidence has been reported since the 1960s. Melanoma makes up approximately 1.7% of all newly diagnosed primary malignant cancers, 0.7% of all cancer deaths, and 90% of deaths from skin tumors worldwide [1,2]. Based on the identification of *BRAF* mutations [3] and the PD-1 immune checkpoint blockade [4], effective therapeutic strategies have been developed in the last decade. These strategies apply selective inhibitors for the MAP kinases BRAF and MEK [5] as well as anti-PD1 antibodies as monotherapy or in combination with anti-CTLA4 antibodies to stimulate the anti-tumor immune response. In this way, the 5-year survival rate of patients with metastatic melanoma could be increased to >30%, or to up to 57% through the use of combination therapies [6,7,8]. However, the new therapies are not suitable for all patients, due to about 50% of patients exhibiting a *BRAF*-WT phenotype, and around half of the patients lacking a sufficient response to immunotherapy [6,9]. Furthermore, tumor relapse and therapy resistance often follow within a few months or years [10], and adverse effects, often related to immunotherapy, affect patient’s quality of life [11]. Therefore, alternative therapies continue to be urgently sought.

The development of cancer is based on multiple cellular mechanisms, including the interruption of proapoptotic pathways, which furthermore plays a decisive role in drug resistance [12]. Therefore, induction of apoptosis through extrinsic or intrinsic apoptosis pathways represents a predominant goal in cancer therapy as a manner in which tumor cells can be eliminated [13]. Extrinsic pathways are triggered by the binding of different death ligands to their cognate death receptors, which leads to the activation of initiator caspase-8 [14]. Initiator caspases mediate the processing and activation of effector caspases, in particular caspase-3, which drives the processing of a long list of death substrates [15].

Along with the induction of apoptosis, DNA repair is abrogated. Thus, the DNA repair enzyme PARP (poly ADP-ribose polymerase) is inactivated through cleavage of the 116 kD form by caspase-3 and -7 to generate 89 kD and 24 kD fragments [16]. DNA strand breaks induced in the course of apoptosis are furthermore accompanied by phosphorylation of histone H2AX (γ-H2AX), which may support apoptotic DNA fragmentation [17,18].

On the other hand, intrinsic apoptosis pathways can be initiated by different types of cellular dysregulation, including DNA damage incurring in the course of chemotherapy. These pathways are characteristically associated with loss of mitochondrial membrane potential and the release of proapoptotic mitochondrial factors, such as cytochrome c [19]. In melanoma cells, anti-tumor strategies have been furthermore related to the production of reactive oxygen species (ROS), which may either accompany apoptosis induction or function as an initial step [20,21].

Mitochondrial apoptosis pathways are critically regulated by the family of Bcl-2 proteins, which includes anti-apoptotic proteins (Mcl-1, Bcl-2, Bcl-x_L_, Bcl-w) and pro-apoptotic multidomain proteins (Bax, Bak), as well as a number of proapoptotic BH3-only proteins (e.g., Bim, Bad, Bid and Puma) [19,22]. According to present models, Bax and Bak can mediate mitochondrial permeability, but are antagonized by antiapoptotic Bcl-2 proteins through heterodimerization. Pro-apoptotic BH3-only proteins can bind to the different anti-apoptotic Bcl-2 proteins to release Bax and Bak and thus function as pro-apoptotic triggers [22]. Due to the important roles of intrinsic apoptosis pathways in melanoma cells, Bcl-2 proteins represent promising targets for melanoma therapy [23,24,25].

Anti-apoptotic Bcl-2 proteins have been frequently reported as being overexpressed in hematological malignancies, as well as in solid tumors [26,27,28,29]. In melanoma, high Bcl-2 expression is characteristic and correlates with apoptosis resistance, invasion, tumor growth, and metastasis [23,30,31]. Mcl-1 and Bcl-x_L_ have also been identified as critical for melanoma cell survival and therapy resistance [32,33]. Bcl-w was reported to cooperate with oncogene activation in the development and progression of cancer [34], and knockdown of Bcl-w has been shown to increase the sensitivity of melanoma cells to chemotherapy [35]. Significant expression of all four proteins has been demonstrated in melanoma cell lines [36,37].

A number of small molecule antagonists, termed “BH3-mimetics”, have been developed to target anti-apoptotic Bcl-2 proteins in cancer. These mimic the BH3 domain of BH3-only proteins and bind to the hydrophobic groove of anti-apoptotic Bcl-2 proteins, inhibiting their activity [29,38]. ABT-737 and ABT-263 (Navitoclax) target Bcl-2, Bcl-x_L_, and Bcl-w, [39,40], while ABT-199 (Venetoclax) is selective for Bcl-2 [41], and S63845 is selective for Mcl-1 [42].

BH3 mimetics have shown efficiency in hematological malignancies and are also considered as candidate drugs for solid tumors such as melanoma. Here, we report impressive efficiency in terms of loss of cell viability, apoptosis induction, and activation of pro-apoptotic pathways when ABT-737, ABT-263, or ABT-199 were combined with the Mcl-1 inhibitor S63845. On the contrary, single treatments were largely ineffective.

## 2. Results

### 2.1. Significant Decrease in Cell Viability Only after Combination of BH3 Mimetics

To evaluate the efficacy of BH3 mimetics in melanoma cells, two *BRAF*-mutated melanoma cell lines (A-375, Mel-HO) and two *BRAF*-WT cell lines (MeWo, SK-Mel-23) were treated with ABT-737, ABT-263, or ABT-199 (ABTs) alone or in combination with S63845. While ABT-737 and ABT-263 inhibit Bcl-2, Bcl-x_L_, and Bcl-w [39,40], ABT-199 was described as specific for Bcl-2 [41], and S63845 selectively targets Mcl-1 [42] (Supplementary Figure S1). The three ABTs were used in increasing concentrations (0.01, 0.1 and 1 µM), while S63845 was used at 1 µM, based on our previous experience with this inhibitor [43].

**Figure 1:**
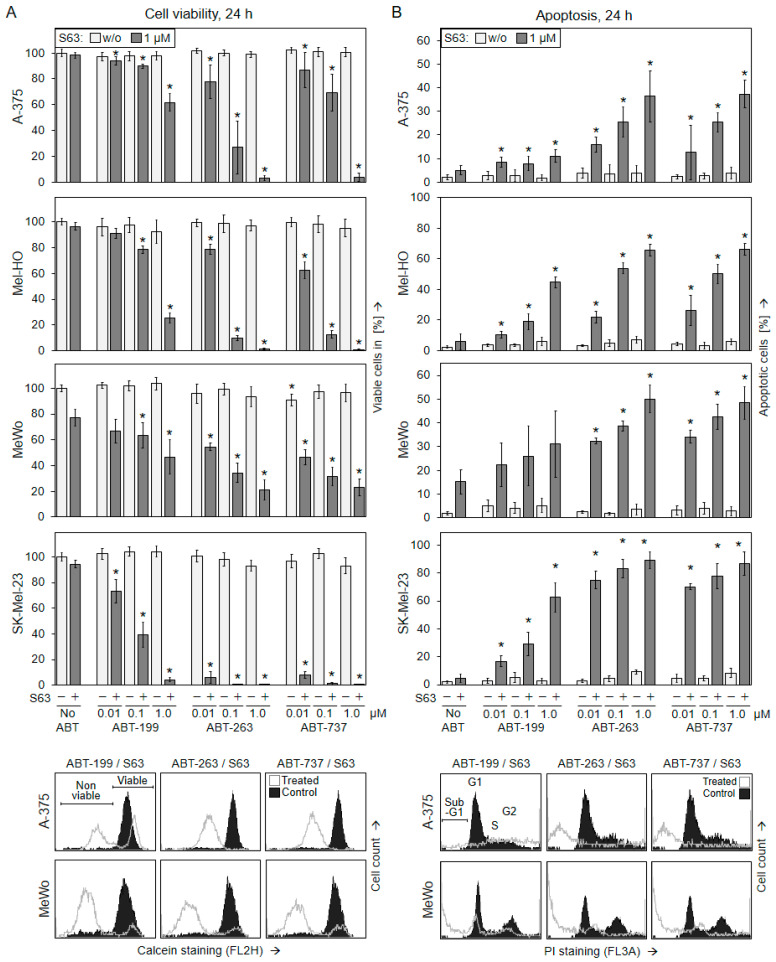
Combinations of BH3 mimetics decrease cell viability and induce apoptosis in melanoma cells at 24 h. Melanoma cell lines A-375, Mel-HO, MeWo, and SK-Mel-23 were seeded in 24-well plates and were treated with S63845 (S63, 1 µM) as well as with ABT-199, ABT-263, or ABT-737 (0.01, 0.1, 1 µM) as indicated. (**A**) After 24 h, cell viability was determined via calcein-AM staining and flow cytometry. Values represent the percentage of cells with high calcein staining (viable cells). Effects on cell viability are displayed as a percentage of non-treated controls (100%). (**B**) After 24 h, apoptotic cells were identified as sub-G1 cells in cell cycle analyses via flow cytometry after propidium-iodide staining. (**A**,**B**) At least two series of experiments were performed, each one consisting of independent triplicate values. Mean values of all individual values (at least 6) are shown here. Statistical significance is indicated by asterisks (*; *p* < 0.05) and was calculated for the single treatments as compared to non-treated control cells. The combination treatments were compared both to the respective single treatments with ABT (light bars) and to S63845 alone. Largely comparable findings were obtained after 48 h treatments (Supplementary Figure S2). Example flow cytometry readings of cells treated for 48 h with combinations of ABTs and S63845 (1 µM concentrations) are shown as overlays vs. controls. Non-viable and viable cell populations as well as cell cycle phases G1 (gap 1), S (synthesis), G2 (gap 2), and sub-G1 cells are indicated.

Only limited responsiveness in terms of reduced cell viability was seen in the four cell lines when these inhibitors were applied alone, as determined at 24 h and 48 h via calcein staining and flow cytometry. ABT-263 and ABT-737 showed some effects in SK-Mel-23, and S63845 slightly reduced cell viability in MeWo (70–80%), while the other single treatments demonstrated almost no effect on cell viability (>90%, Figure 1A; Supplementary Figure S2A).

In clear contrast, cell viability was strongly decreased at 24 h and 48 h when the three ABTs were combined with S63845. At 48 h, the combinations of ABT-263/S63845 and ABT-737/S63845 almost completely abolished cell viability in A-375, Mel-HO, and SK-Mel-23, as well as decreased it to around 20% in MeWo (1 µM). The combination of ABT-199/S63845 was strongly effective in SK-Mel-23 and Mel-HO, and also decreased cell viability to around 45% in A-375 and MeWo (Figure 1A; Supplementary Figure S2A). The three drug combinations showed signs of possible synergism in terms of reduced cell viability, indicated by combination indices of <1 for the tested concentrations (Supplementary Figure S3A).

### 2.2. Decreased Cell Viability Correlates with Induction of Apoptosis

To unravel the causes of loss of cell viability, induction of apoptosis was determined at 24 h and 48 h via cell cycle analysis. When evaluating the sub-G1 cell fraction, which corresponds to apoptotic cells with fragmented DNA, again only limited effects were seen after single treatments. In agreement with the cell viability data, ABT-263 and ABT-737 induced some apoptosis in SK-Mel-23, and S63845 induced some apoptosis in MeWo (10–20% 48 h, 1 µM), while effects of other single treatments were even smaller (Figure 1B, Supplementary Figure S2B).

Clearly supporting the cell viability data, apoptosis was strongly enhanced in the four cell lines after combination treatments. ABT-263/S63845 and ABT-737/S63845 increased apoptosis rates to 50% in A-375 and MeWo and up to 90% in Mel-HO and SK-Mel-23 (1 µM, 48 h). The effects of ABT-199/S63845 were somewhat less pronounced, with 72% apoptosis observed in Mel-HO, 81% in SK-Mel-23, 34% in MeWo, and 15% in A-375 (Figure 1B, Supplementary Figure S2B). The reduced apoptotic response of A-375 to this Bcl-2-selective drug was in agreement with the weak expression of Bcl-2 in A-375, as we had described previously [36]. Also in terms of apoptosis induction, the three drug combinations showed signs of possible synergism, with combination indices of <1 for the used concentrations (Supplementary Figure S3B). Further cell cycle analysis (quantification of G1 and G2 cell cycle phases) performed after PI staining revealed some decrease in G1 mediated by S63845 in all four cell lines, by ABT-199 in MeWo, as well as by ABT-263 and ABT-737 in SK-Mel-23, while other single treatments remained ineffective. Most striking, however, was the strong loss of both G1 and G2 after combination treatment (Supplementary Figure S4), which went hand in hand with strongly induced apoptosis. All cell cycle profiles after treatment with the different BH3 mimetics are shown in Supplementary Figure S5. The results indicated that induction of apoptosis and not cell cycle arrest was the major effect.

To confirm apoptosis induction by a second assay and to distinguish between early and late apoptotic cells, Mel-HO and SK-Mel-23 were investigated using Annexin V-FITC/PI staining (AnnV/PI) at 6 h, 12 h, and 24 h. Early apoptotic cells were identified as AnnV(+)/PI(−), while late apoptotic or necrotic cells were characterized as AnnV(+)/PI(+).

**Figure 2:**
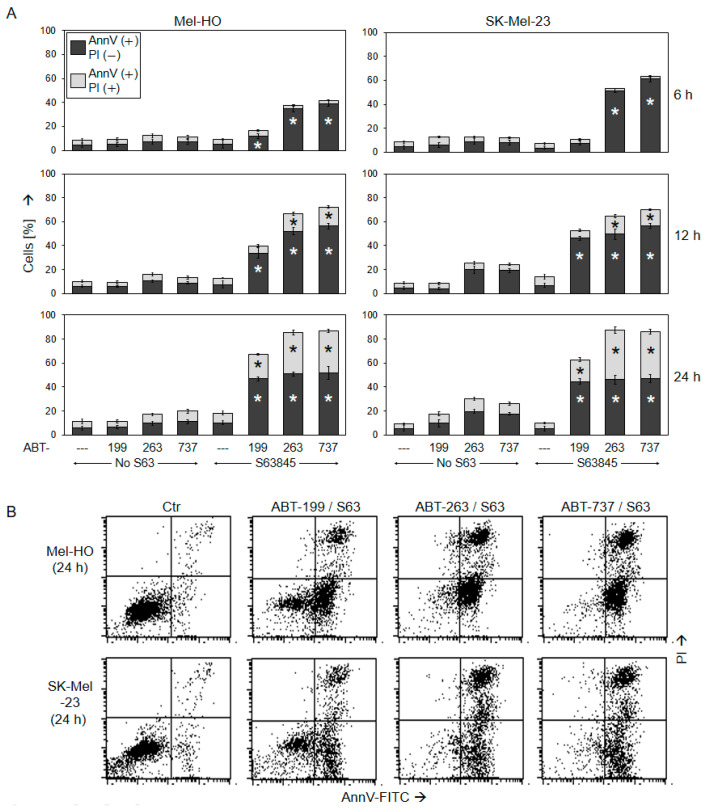
Early induction of apoptosis plays a leading role. Mel-HO and SK-Mel-23 cells were seeded in 24-well plates treated with ABT-199, ABT-263, ABT-737, and/or S63845 (1 µM concentrations; ---, no ABTs were added). (**A**) Cell death analysis by Annexin V/PI staining and flow cytometry was performed at 6 h, 12 h, and 24 h. Early apoptotic cells were determined as AnnV(+)/PI(−), while late apoptotic or necrotic cells corresponded to AnnV(+)/PI(+) staining. Mean values (in %) and SDs were calculated of three series of experiments, each one consisting of double values (six values in a group). Statistically significant changes observed in combination treatments, as compared to the respective single treatment with ABT and to S63845 alone, are indicated for the two cell death fractions (*; *p* < 0.01). (**B**) Representative flow cytometry histograms of combination-treated cells and control cells are shown. Readings are separated into four quadrants (according to PI and AnnV positivity). Thus, the lower left quadrant corresponds to AnnV(−)/PI(−) cells (viable cells), the lower right quadrant corresponds to AnnV(+)/PI(−) cells (early apoptosis), and the upper right quadrant corresponds to AnnV(+)/PI(+) cells (late apoptosis).

For ABT-263/S63845 and ABT-737/S63845, the decisive role of early apoptosis was clearly visible at 6 h, when up to 40% of AnnV(+)/PI(−) cells were identified in Mel-HO and up to 60% were found in SK-Mel-23 (Figure 2). For ABT-199/S63845, the AnnV(+)/PI(−) cell fraction was strongly induced at 24 h to 34% and 46% in Mel-HO and SK-Mel-23, respectively. In contrast, there was no induction of AnnV(+)/PI(+) cells at 6 h, but the number of late apoptotic cells increased with time. When all AnnV(+) cells were considered (early and late apoptosis), cell death at 24 h was at 60–90% for all combinations (Figure 2). The combination effects of BH3 mimetics on cell viability and apoptosis were always far more than additive. Thus, apoptosis induction appeared as the decisive effect for BH3 mimetic combinations in melanoma cells.

### 2.3. Activation of Mitochondrial Apoptosis Pathways

**Figure 3:**
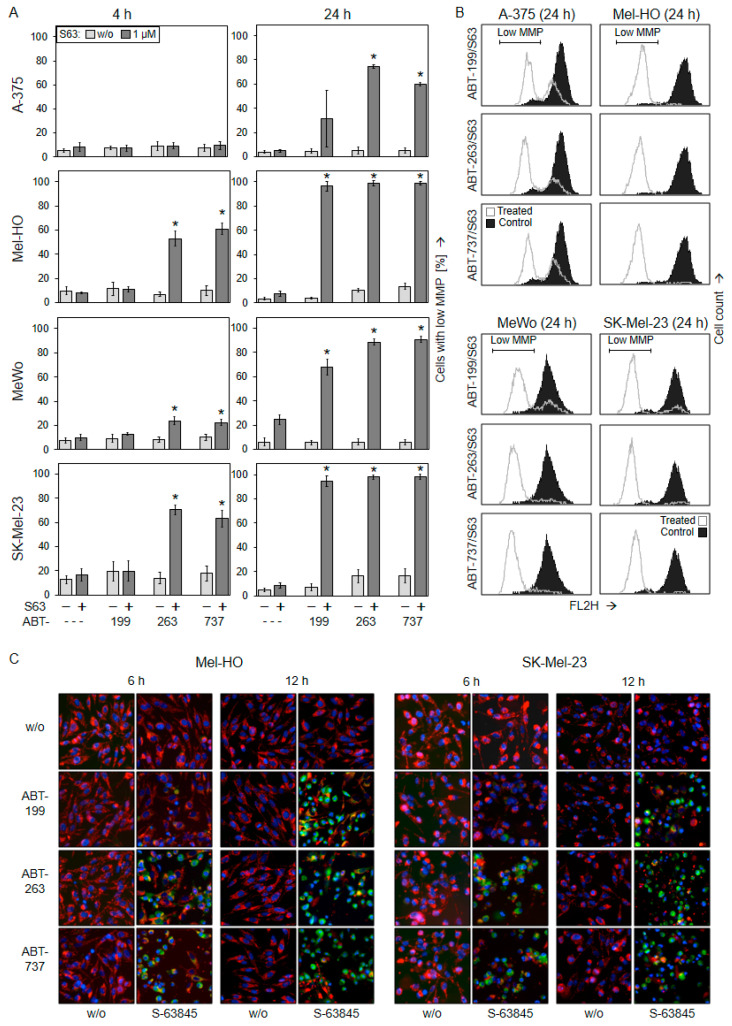
Combinations of BH3 mimetics induce mitochondrial apoptosis in vitro. The four cell lines were treated with S63845 (S63), ABT-199, ABT-263, ABT-737, and combinations (1 µM concentrations; ---, no ABTs were added). (**A**) Mitochondrial membrane potential (MMP) was determined at 4 h and at 24 h via TMRM^+^ staining and flow cytometry. Values represent the percentage of cells with low MMP. Mean values and SDs (in %) were calculated for two series of experiments, each one consisting of independent triplicate values (six values in a group). Statistical significance (* *p* < 0.05) was calculated for combination treatments as compared to the respective single treatments (ABTs alone and S63845 alone). (**B**) Examples of flow cytometry readings are shown as overlays of combination treatments (dark graphs) vs. the controls (open graphs). Cell populations with low MMP are indicated. (**C**) For microscopic visualization of low MMP, Mel-HO and SK-Mel-23 cells were stained with JC-1 and counterstained with Hoechst-33342 at 6 h and at 12 h of treatment. Cell nuclei are stained in blue, while mitochondria with high (normal) MMP are stained in red. Green staining indicates cytosolic JC1 localization upon release of JC1 from mitochondria with low MMP. Two independent experiments revealed highly comparable results.

The loss of mitochondrial membrane potential (MMP) is characteristic for intrinsic/mitochondrial apoptosis pathways. The alterations in MMP were determined through TMRM^+^ staining and flow cytometry at 4 h and at 24 h in response to the BH3 mimetic treatments (1 µM concentrations). Single treatments had only limited effects on MMP, as compared to non-treated controls. Only MeWo showed 25% of cells with low MMP at 24 h in response to S63845 alone. In clear contrast, combination treatments resulted in strong loss of MMP at 24 h, indicating the involvement of mitochondrial apoptotic pathways. In response to combination treatments, there were 30–75% cells with low MMP in A-375, as well as 70–90% in MeWo and >94% in Mel-HO and SK-Mel-23. The stronger responsiveness of Mel-HO and SK-Mel-23 was underlined at 4 h, when ABT-263/S63845 and ABT-737/S63845 induced 50–70% of cells with low MMP (Figure 3A,B).

Loss of MMP caused by combination treatments was further visualized in Mel-HO and SK-Mel-23 via JC-1/Hoechst-33342 double staining at 6 h and 12 h. While cell nuclei are stained blue by Hoechst-33342, the cationic dye JC-1 forms red fluorescent aggregates in mitochondria of viable cells. Upon loss of MMP, JC-1 locates to the cytosol, where fluorescence shifts from red to green. In response to ABT-263/S63845 and ABT-737/S63845 (1 µM concentrations), both cell lines showed clear signs of MMP loss (green cells) at 6 h. This was further enhanced at 12 h, when all three combinations resulted in strong induction of green cells, while single treatments remained without effect (Figure 3C). Thus, the assay indicated early loss of MMP induced by ABT-263/S63845 and ABT-737/S63845, while the effects of ABT-199/S63845 were somewhat delayed (12 h).

**Figure 4:**
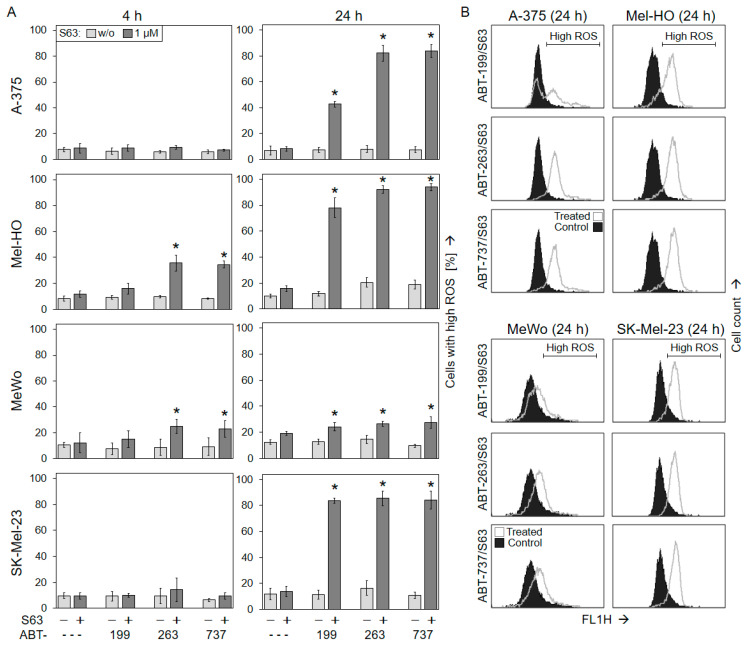
Reactive oxygen species are produced after combination treatment. A-375, Mel-HO, MeWo, and SK-Mel-23 cells were treated with ABT-199, ABT-263, ABT-737, S63845 (S63), and combinations (1 µM concentrations; ---, no ABTs were added). (**A**) Cellular levels of ROS were determined at 4 h and at 24 h via H_2_DCF-DA staining and flow cytometry. Values represent the percentage of cells with high ROS. Mean values and SDs (in %) were calculated for two independent experiments, each one consisting of triplicate values (six values in a group). Statistical significance (* *p* < 0.05) was calculated for combination treatments as compared to the respective single treatments (ABTs alone and S63845 alone). (**B**) Examples of flow cytometry readings are shown on the right side for the combination treatments (dark graphs) vs. the controls (open graphs). Cell populations with high ROS are indicated.

Production of reactive oxygen species (ROS) may accompany and support the activation of intrinsic apoptosis pathways. ROS levels were also determined at 4 h and at 24 h via flow cytometry after H_2_DCF-DA staining. While the single treatments were not very effective (<21%), ROS levels were significantly elevated at 24 h in A-375, Mel-HO, and SK-Mel-23 in response to the combination treatments (1 µM concentrations). ROS levels were increased in up to 84% of cells in A-375, 94% in Mel-HO, and 86% in SK-Mel-23. Only MeWo showed lower ROS production (<28%; Figure 4), which may be related to a *p53* mutation in this cell line [44]. The role of ROS in apoptosis induction could not be clearly shown, because ROS could not be completely abolished by the common antioxidant N-acetylcysteine (1 mM). Thus, NAC did also not prevent loss of cell viability and induction of apoptosis in course of combination treatments with BH3 mimetics (Supplementary Figure S6). In the three responsive cell lines, mitochondrial pathways may be supported by high ROS levels. However, early ROS production (4 h) was not seen, which may exclude the role of ROS as an initiating step in apoptosis induction in these settings.

### 2.4. Crucial Role of Caspase-Mediated Pathways

**Figure 5:**
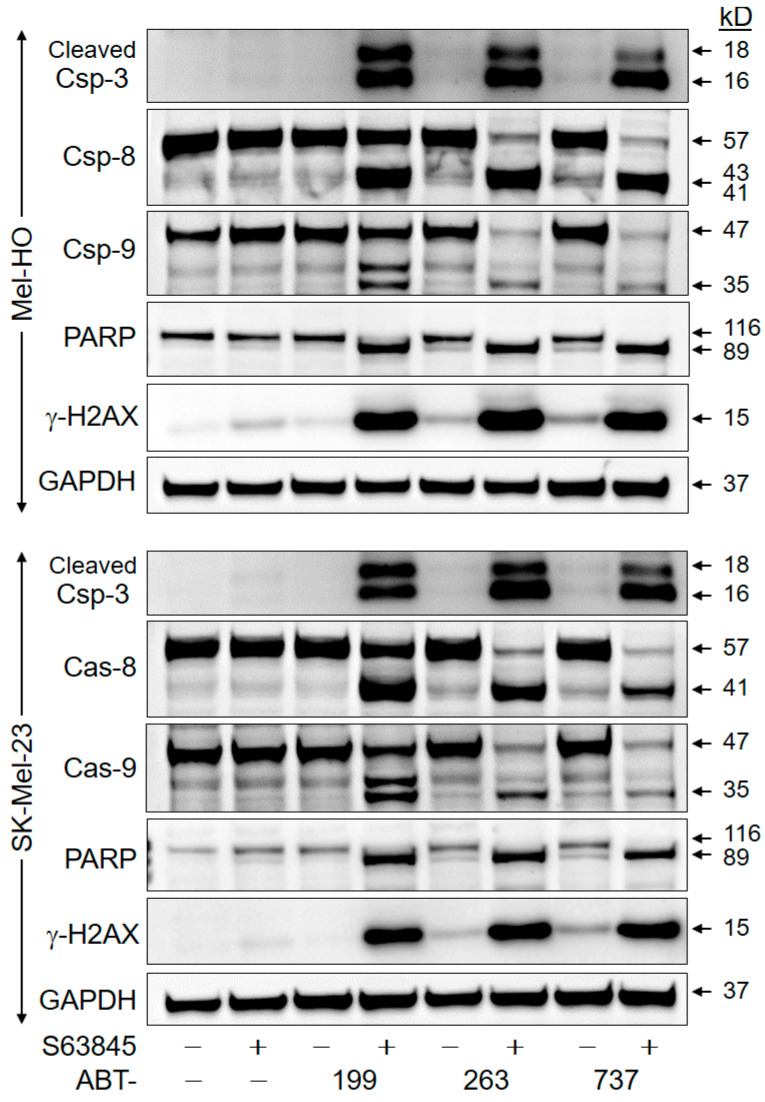
Pro-apoptotic pathways are efficiently activated only after combination treatment. Mel-HO and SK-Mel-23 cells were treated for 8 h with ABT-199, ABT-263, ABT-737, S63845, and combinations (1 µM concentrations). Total protein extracts were analyzed by Western blotting for cleaved caspase-3, total caspase-8, and caspase-9 (Csp). Further, processing of PARP from 116 to 89 kD and phosphorylation of histone H2AX (γ-H2AX) were analyzed. Equal protein amounts (30 µg per lane) were separated by SDS-PAGE, and consistent blotting was proven by Ponceau staining as well as via evaluation of GAPDH expression. Molecular weights are indicated in kD. Each two independent series of protein extracts and Western blots revealed highly comparable results.

Activation of proapoptotic caspases was investigated by Western blotting in Mel-HO and SK-Mel-23 after 8 h of treatment. Indicating extensive caspase activation, particularly high expression of the activated cleavage products of the major effector caspase-3 (16/18 kD) was seen in both cell lines in response to combination treatments. In contrast, almost nothing was seen after single treatments. This correlated with the activation of caspase-8 (cleavage products of 41/43 kD) and the initiator caspase caspase-9 (cleavage product of 35 kD). Caspase activation was further made clear by the complete processing of PARP (poly ADP-ribose polymerase), a characteristic death substrate and target of caspase-3, from 116 kD to 89 kD (Figure 5). Quantitative analyses for all Western blot analyses are displayed in Supplementary Table S1.

Phosphorylation of histone H2AX (γ-H2AX) characteristically coincides with DNA breaks in apoptotic cells and may be required for DNA fragmentation [17,18]. Clearly indicating induction of this pathway, γ-H2AX was strongly induced after combination treatments, while little effect was seen following single treatments (Figure 5).

**Figure 6:**
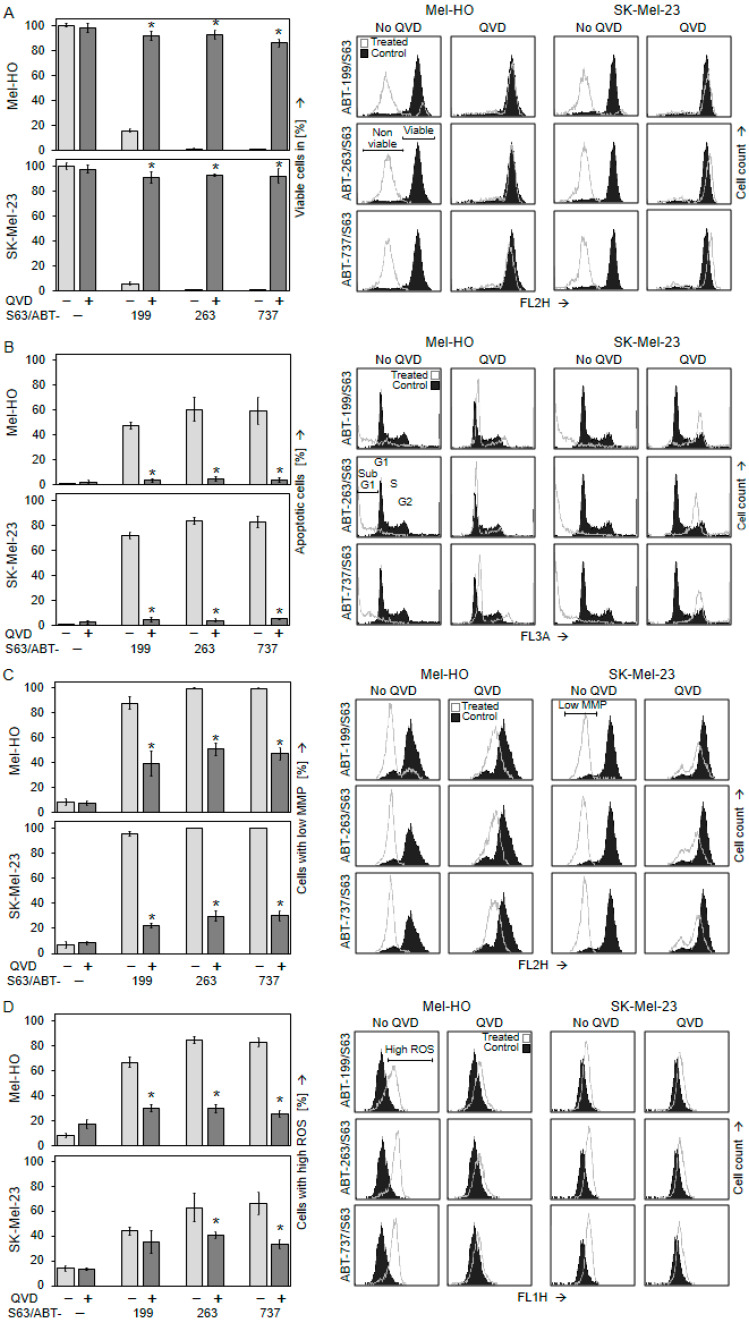
Cell death induced by BH3 mimetic combinations in melanoma cells is caspase-dependent in vitro. Mel-HO and SK-Mel-23 cells were treated with combinations of S63845 with ABT-199, ABT-263, and ABT-737 (1 µM concentrations). When indicated, the pan-caspase inhibitor QVD-Oph (QVD, 5 µM) was applied at 1 h before other treatments started. Cell viability (calcein staining; (**A**)), apoptosis induction (cell cycle analysis; (**B**)), loss of MMP (TMRM^+^ staining; (**C**)) and production of ROS (H_2_DCF-DA staining; (**D**)) were determined via flow cytometry at 24 h. Mean values and SDs (in %) were calculated from two series of experiments, each one consisting of independent triplicate values. Statistical significance (* *p* < 0.05) was calculated for QVD treatments as compared to ABT/S63845 combinations without QVD (at least 6 individual values in a group). On the right side, example flow cytometry readings of ABT/S63845 combination treatments +/− QVD are shown as overlays. Viable and non-viable cell populations (**A**), sub-G1, G1, S and G2 cell populations (**B**), cell populations with low MMP (**C**), and cell populations with high ROS levels (**D**) are indicated.

The significance of caspase activation for anti-tumor effects was proven by the use of the pan-caspase inhibitor QVD-Oph (QVD, 5 µM). The strong loss of cell viability due to the ABT/S63845 combination was almost completely prevented in Mel-HO and SK-Mel-23 by QVD (86–98% cell viability; Figure 6A). Similarly, apoptosis induction in response to the combinations (48–83%) was completely abolished (<5%; Figure 6B).

This was further supported when other steps of apoptosis pathways were highlighted. Loss of MMP caused by ABT-199/S63845, ABT-263/S63845, and ABT-737/S63845 in Mel-HO and SK-Mel-23 was strongly diminished by QVD. In Mel-HO, the number of cells with loss of MMP decreased from 88–99% to 39–51%, and in SK-Mel-23, it decreased from 96–100% to 22–30% (Figure 6C). Similarly, induction of ROS was diminished in Mel-HO from 67–85% to 26–30%. In SK-Mel-23, ROS induced by ABT-263/S63845 was decreased from 63% to 41%, and ROS induced by ABT-737/S63845 was decreased from 66% to 34% (Figure 6D). Further quantification of G1 and G2 cell cycle phases after PI staining in cell cycle analyses also revealed that the strong loss of G1 and G2 due to combination treatment and induced apoptosis was almost completely reverted by QVD-Oph (Supplementary Figure S7). This underlines that caspase activation was the critical step facilitating the effects of combinations of BH3 mimetics in melanoma cells.

### 2.5. Downregulation of Anti-Apoptotic Factors

**Figure 7:**
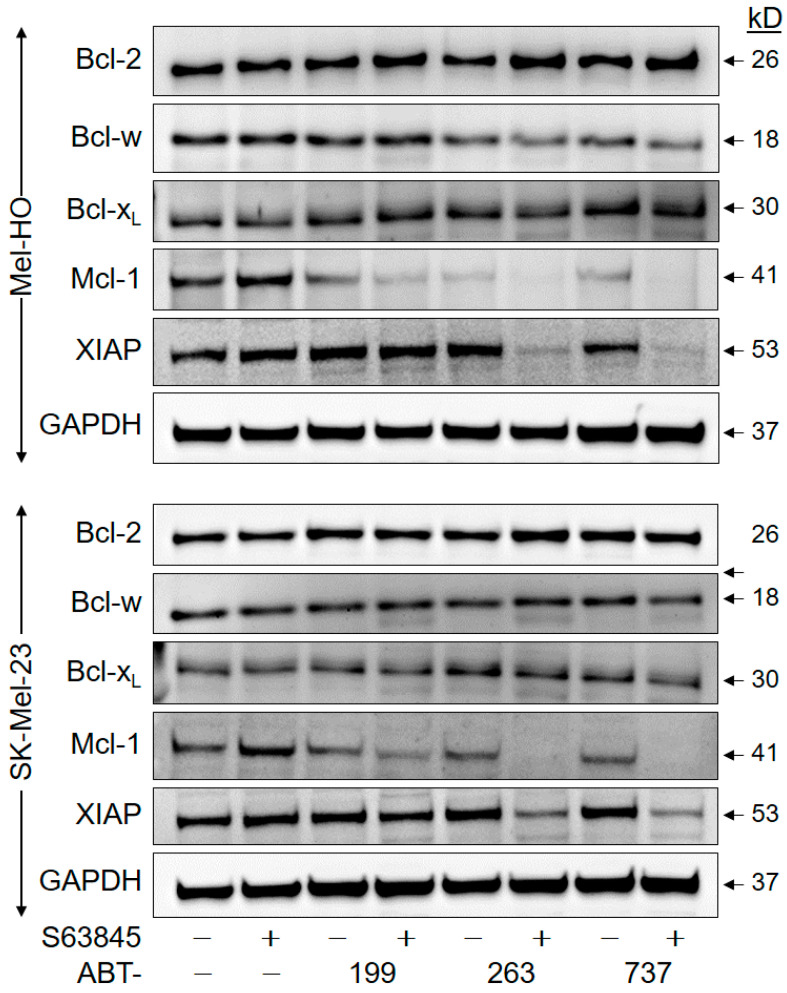
Apoptosis proteins Mcl-1 and XIAP are downregulated by combination treatments. Mel-HO and SK-Mel-23 cells were treated with ABT-199, ABT-263, ABT-737, S63845, and combinations (1 µM concentrations, treatment time: 8 h). Total protein extracts were analyzed by Western blotting for expression of Mcl-1 (41 kD), Bcl-2 (26 kD), Bcl-w (18 kD), Bcl-x_L_ (30 kD), and XIAP (53 kD). Equal protein amounts (30 µg per lane) were separated by SDS-PAGE, and consistent blotting was proven via Ponceau staining as well as through evaluation of GAPDH expression. Molecular weights are indicated in kD. Two independent series of protein extracts and Western blots revealed highly comparable results.

To investigate whether the effects of BH3 mimetics were further related to the expression of anti-apoptotic Bcl-2 proteins or inhibitors of apoptosis proteins, Bcl-2, Bcl-w, Bcl-x_L_, and Mcl-1 as well as XIAP (X-linked inhibitor of apoptosis protein) were investigated via Western blotting in Mel-HO and SK-Mel-23 at 8 h of treatment with ABTs +/− S63845 (1 µM concentrations). As reported previously, Mcl-1 protein was upregulated in response to its inhibition by S63845, which may indicate an inhibition of Mcl-1 degradation and may diminish the inhibitory effect of S63845 [42,45]. Median upregulation of Mcl-1 in Mel-HO and SK-Mel-23 was at 1.4, as determined by densitometric analyses and normalization using the GAPDH signals from two independent series of protein extracts and Western blots. Importantly, upregulation of Mcl-1 was completely abolished by combination treatments with ABTs. Mcl-1 expression was downregulated by single treatments with ABT-199, -263 and -737 to 60%, 29%, and 22%, respectively, in Mel-HO as well as to 86%, 63%, and 62%, respectively, in SK-Mel-23. The downregulation was further strengthened by combination treatments of ABT-199/ABT-263/ABT-737 with S63845, resulting in Mcl-1 expression values of 29%/5%/4%, respectively, in Mel-HO and 38%/13%/5%, respectively, in SK-Mel-13 (as compared to controls; Figure 7). In comparison, no comparable downregulation was seen for Bcl-2, Bcl-w or Bcl-x_L_ (Supplementary Table S1). However, the caspase-3 antagonist XIAP also showed clear downregulation at 8 h in response to ABT-737/S63845 and ABT-263/S63845 in both cell lines (Mel-HO, 16%/20%; SK-Mel-23, 20%/15%. Figure 7; Supplementary Table S1). These data suggest that the pro-apoptotic activity of BH3 mimetics may be supported by downregulation of anti-apoptotic Mcl-1 and XIAP. Mcl-1 downregulation in this setting appeared to be caspase-dependent, as shown for Mel-HO and SK-Mel-23 treated with the ABT-737/S63845 combination. Cells were additionally treated with either the pan-caspase inhibitor QVD-Oph or the proteasome inhibitor MG132. In subsequent WB analysis, we found the 17 kD caspase cleavage product of Mcl-1 [46] appearing together with Mcl-1 degradation, and this was abrogated by QVD-Oph, while the proteasome inhibitor had no effect (Supplementary Figure S8).

## 3. Discussion

In the last decade, BRAF and immune-checkpoint inhibitors have dramatically improved therapy for, and the prognosis of, patients with metastasized melanoma, leading to 5-year survival rates of presently up to 57% [6,7]. Nevertheless, due to the *BRAF*-WT phenotype of about 50% [9] of patients and more than half of patients lacking sufficient response to immunotherapy [6], large therapeutic gaps have remained, which may be closed by new strategies, e.g., BH3 mimetics.

Apoptosis deficiency plays an elementary role in cancer development and drug resistance, and accordingly, induction of apoptosis represents a common and efficient way to eliminate cancer cells [12,25]. Suppression of apoptosis may depend on amplification or overexpression of anti-apoptotic Bcl-2 family proteins such as Bcl-2, Mcl-1, Bcl-x_L_, and Bcl-w, which often correlates with poor prognosis and therapy resistance [19,22,47].

BH3 mimetics represent a direct strategy to inactivate anti-apoptotic Bcl-2 family proteins, and they have already been proven as clinically effective against a number of tumors [29]. ABT-737, which targets Bcl-2, Bcl-x_L_, and Bcl-w, was the first validated BH3 mimetic [39], and its orally bioavailable analogue ABT-263 (Navitoclax) was the first BH3 mimetic to achieve clinical use [40,48]. Its clinical use was, however, ultimately limited due to the inhibition of platelets, whose survival depends on Bcl-x_L_ [48,49], a problem that may be overcome by combinations as shown here. On the other hand, the Bcl-2-selective ABT-199 (Venetoclax) does not affect platelets [41]. It has been approved for the treatment of chronic lymphocytic leukemia and, in combination with a hypomethylating agent, for the treatment of elderly patients with acute myeloid leukemia [50]. In addition, it has shown promising clinical results in solid tumors, e.g., in breast cancer [51], small-cell lung cancer [52], and neuroblastoma [53].

However, while ABT-263 and ABT-737 have demonstrated single-agent efficacy against leukemia and lymphoma, they have remained ineffective in treating solid tumor cells, as observed in breast cancer, rhabdomyosarcoma, and neuroblastoma [54]. Still more experimental data are needed to identify the most suitable strategies for each tumor type. A few in vitro studies have already been performed to address this question in melanoma cells [33,55,56]. In the present manuscript, we clearly demonstrate in four melanoma cell lines (BRAF-mutated and BRAF-WT) that combinations of BH3 mimetics are strongly effective, while single treatments are largely inefficient. The combination of the selective Mcl-1 inhibitor S63845 with three different ABTs (ABT-199, -263, and -737) has revealed particularly high efficiency. Based on our experiments, it appears that this is the most promising strategy for targeting all anti-apoptotic Bcl-2 proteins. The combinations of ABT-263/S63845 and ABT-737/S63845 were most effective. It may be justified to consider these drug combinations as therapy options for both BRAF-mutated and BRAF-WT melanomas.

Special emphasis was placed here on the understanding of the downstream pathways involved, which may allow improved application of these strategies. Thus, we present here a comprehensive analysis of cell death pathways activated by combinations of BH3 mimetics in melanoma cells. Loss of cell viability and apoptosis induction were always correlated, and multiple pro-apoptotic pathways were shown to be activated, as seen in the loss of mitochondrial membrane potential, ROS production, phosphorylation of histone H2, caspase-3, -8, and -9 activation, and PARP cleavage, as well as XIAP and Mcl-1 downregulation. This clearly underlines the comprehensive effects of BH3 mimetics in melanoma cells. In particular, the essential, upstream role of caspase activation was proven, as caspase inhibition almost completely abolished apoptosis, restored cell viability, and blocked cell death pathways.

As for Mcl-1, major roles have been described in the sustained growth and therapy resistance of different tumors, and several studies have indicated its activation in tumor cells, related to chromosomal amplification, increased mRNA, and protein expression [32,57,58]. High levels of Mcl-1 may further correlate with resistance to ABTs, as reported for cells of ALL [59], CLL [60], and lung and breast cancers [61,62].

The development of the Mcl-1 inhibitor S63845 has enabled the selective inhibition of Mcl-1 in cancer cells, and S63845 has shown high efficacy in a panel of hematological cancer cell lines [42,63]. The chemically related inhibitor S64315 (MIK665) reveals comparable activity [64] and is presently being tested in lymphoma, multiple myeloma, and AML patients [63]. Other Mcl-1-specific BH3 mimetics (AMG-176, AMG-397 and AZD-5991) have entered clinical evaluation for patients with hematological malignancies [65].

We show here, as well as in a previous study [43], that S63845 single treatment has minimal effects in melanoma cell lines in terms of induction of apoptosis, loss of cell viability, and activation of cell death pathways. Only one of the four cell lines (MeWo) showed a moderate response. In other melanoma cell line panels, only 14% have shown limited sensitivity to S63845 single treatment [66]. Some melanoma cell lines have revealed resistance to S63845 in concentrations up to 5 μΜ, as shown by cell viability and activity assays [33]. It thus appears quite clear that melanoma cell survival and apoptosis deficiency cannot be overcome by inhibition of just one specific anti-apoptotic Bcl-2 protein; the activity of all of these proteins must be interrupted. Indeed, significant expression of both Bcl-2, Bcl-x_L_, Mcl-1, and Bcl-w is characteristic in melanoma cell lines [36,37].

These findings indicate that the use of combinations of BH3 mimetics in melanoma therapy could be effective. Several studies on human cancer cells and mouse models have highlighted the effectiveness of co-targeting anti-apoptotic Bcl-2 proteins, as in osteosarcoma, neuroblastoma, and lung and colorectal carcinomas [67,68,69]. In melanoma cells, Mcl-1 and Bcl-x_L_ have been suggested as critical for melanoma cell survival, based on reduced cell viability and induced apoptosis when S63845 was combined with ABT-263 or ABT-199 [33,70]. The combination of ABT-199 with a Mcl-1 inhibitor has already advanced into phase I clinical trials for patients with AML [71]. The concentrations we used here agree with the literature (0.1–10 µM). Also from a pharmacological point of view, a concentration of 1 µM should be realistic, although it always remains difficult to take into account half-life and distribution of a drug in the body, as well as inactivation and excretion.

In our study, the combination of S63845 with ABT-263, ABT-737, and ABT-199 showed high efficiency both in *BRAF*-mutated and *BRAF*-WT melanoma cell lines, as evidenced by induced apoptosis in up to 94% of the cells and almost complete loss of cell viability. Although cell culture models for induced melanoma cell drug resistance have not been tested here, there may be a legitimate hope that different kinds of drug resistance in melanoma cells may also be overcome by these combination treatments, as the targets of BH3 mimetics are clearly distinct from those of BRAF inhibitors and immunotherapy. At least the pre-existing resistance to BRAF inhibitors due to *BRAF*-WT phenotype (cell lines A-375 and SK-Mel-23) was overcome by the combination therapies in the present study. Concerning the suitability of animal experiments, we think that the significance would be limited. Firstly, relatively good clinical tolerability of BH3 mimetics has already been shown in clinical trials for other tumors [29]. Secondly, animal models of course differ greatly from humans in many aspects. Particular differences concern the sequence and function of Bcl-2 proteins in mouse cells, which makes it difficult to decide which therapy may be effective. The mechanistic details and the selection of the best combination partners may be better investigated in vitro. Here, we demonstrate the particularly high efficacy of several combinations; the next steps could be clinical studies in melanoma patients.

The activation of cell death pathways by the here used combinations was, up until now, less investigated in melanoma cells. However, activation of caspases and mitochondrial pathways has been reported in neuroblastoma cells in response to the combination of S63845 with the Bcl-x_L_ inhibitor A1331852 [72], as well as in renal cell carcinoma cells in response to the combination of ABT-263 with TW-37 (targeting Mcl-1, Bcl-x_L_, and Bcl-2) [73]. In rhabdomyosarcoma cells, treatment with a combination of S63845 and the Bcl-x_L_ inhibitor A-1331852 resulted in loss of MMP and activation of caspase-3, -8, and -9 [68].

In melanoma cells, cleavage of caspase-3 and PARP were reported when cells were treated with the Mcl-1 inhibitor A-1210477 in combination with ABT-263 [74], and in activity assays, activated caspase-3/7 was reported after combined S63845/ABT-263 treatment [55]. Enhanced caspase-3 activation and mitochondrial activation was also reported in response to combinations of S63845 with MAP kinase inhibitors [43,75,76], as well as in response to combinations of S63845 with bromodomain inhibitors [66]. Loss of mitochondrial membrane potential was found in A-375 melanoma cells in response to the BH3 mimetic gossypol [77], as well as in response to the Mcl-1 antagonist obatoclax in combination with tunicamycin [78].

Loss of mitochondrial membrane potential may be associated with ROS production [79]. While ROS production was discussed as an initial step in apoptosis pathways in melanoma cells in response to other treatments [20,80], here, ROS may play only a contributory role, as they were not detected at early phases of treatment. The question of why MeWo exhibited only minimal ROS production in response to BH3 mimetics may be explained by its *p53* mutation. It has been shown that in MeWo cells, mutant *p53* increases the expression and the enzymatic activity of the key antioxidant detoxifying enzyme manganese superoxide dismutase (MnSOD). This may keep the ROS levels that are typically induced by BH3 mimetics under control [44]. While the effects on ROS in response to the inhibitor combinations used here had not previously been investigated, some increase in ROS levels has been reported in melanoma cells in response to the Mcl-1/Bcl-x_L_/Bcl-2-selective BH3 mimetic TW-37 [81]. Also, increased ROS levels were reported in Jurkat cells in response to high dose ABT-737 [82].

We show here Mcl-1 upregulation in response to S63845 treatment alone, as also reported previously in breast, colon, rhabdomyosarcoma, and T-ALL tumor cell lines [42,83,84,85]. This upregulation was here completely abolished in combination treatments with the ABTs. It is clear that Mcl-1 downregulation can sensitize melanoma cells for apoptosis, as seen also by siRNA strategies [36,37,86]. Mcl-1 downregulation in melanoma cells was also seen in response to some other pro-apoptotic strategies, including treatment with TRAIL (TNF-related apoptosis inducing ligand) in combinations with kinase inhibitors such as vemurafenib, indirubin, or BMS-345541 [20,87,88]. Expression of Mcl-1 is tightly regulated at the transcriptional, post-transcriptional, and post-translational levels. Thus, phosphorylation sites in a Mcl-1 PEST (proline, glutamic acid, serine, and threonine-rich) domain can be targeted by different kinases, resulting in Mcl-1 degradation and induction of apoptosis. Mcl-1 degradation is frequently mediated by the proteasomal pathway, which may be induced by phosphorylation of the PEST domain or through binding by proapoptotic Bcl-2 proteins and BH3 mimetics. Finally, Mcl-1 is a well-described target of caspase activity and is degraded in course of induced apoptosis [45]. In our experiments, Mcl-1 downregulation appeared to be related to induced caspase activity.

The significant role of the caspase-3 antagonist XIAP in apoptosis resistance in melanoma cells has been shown by siRNA knockdown [25]. Our study also shows strong downregulation of XIAP in response to treatments with ABT-263/S63845 and ABT-737/S63845, suggesting its role in caspase-3 activation. Downregulation of XIAP in melanoma cells was also seen in response to TRAIL in combination with kinase or HDAC inhibitors [20,88,89]. Thus, pronounced activation of apoptosis pathways (caspases, PARP, mitochondrial membrane potential, ROS, and histone H2AX, as well as downregulation of XIAP and Bcl-2, prove the decisive role of apoptosis in this setting. Furthermore, apoptosis induction and loss of cell viability were observed largely in parallel.

## 4. Materials and Methods

### 4.1. Cell Culture and Treatment

In the present study, the *BRAF*-mutated melanoma cell lines A-375 [90] and Mel-HO [91], as well as the *BRAF*-WT cell lines MEWO [92] and SK-MEL-23 [93], were used. Cells were cultured at 37 °C, 5% CO_2_, in DMEM growth medium with 4.5 g/L glucose (GIBCO, Invitrogen, Karlsruhe, Germany), supplemented with 10% fetal calf serum and antibiotics.

Cells were treated with ABT-737 (Biozol, Eching, Germany) and ABT-263 (Navitoclax; Biozol), which target Bcl-2, Bcl-x_L_, and Bcl-w, as well as with the Bcl-2-selective inhibitor ABT-199 (Venetoclax; Selleck Chemicals, Houston, TX, USA) and the Mcl-1-selective inhibitor S63845 (HY-100741; MedChemExpress, Cologne, Germany). The chemical structures of the substances are given in the Supplementary Figure S1. Concentrations were raised up to 1 µM, and control cells received the solvent DMSO. To investigate the roles of caspases, the pan-caspase inhibitor QVD-Oph (Abcam, Cambridge, UK; 5 μM) was applied 1 h before BH3 mimetics were given. For the experiment in Supplementary Figure S8, the proteasome inhibitor MG132 (Merck, Darmstadt, Germany, 2 μM) was added 1 h before treatment. For most analyses, 5 × 10^4^ cells per well were seeded in 24-well plates.

### 4.2. Quantification of Cell Viability and Apoptosis

Cell viability was quantified using flow cytometry after staining cells with calcein-AM (BD Biosciences, Heidelberg, Germany). While in viable cells calcein-AM is converted by intracellular esterases to the green-fluorescent calcein; non-viable cells are not stained. Cells grown in 24-well plates were harvested by trypsinization at 24 h or 48 h after treatment and were stained with 0.5 µM calcein-AM at 37 °C. After 1 h, cells were washed with PBS and were analyzed via flow cytometry (FACS Calibur; BD Bioscience, Heidelberg, Germany; FL2H).

Induction of apoptosis was determined by cell cycle analyses. Cells were harvested by trypsinization and were lysed in a hypotonic PI solution (0.1% sodium citrate, 0.1% triton-x100, 40 µg/mL propidium iodide, in PBS). In this way, isolated nuclei were stained for at least 1 h at 4 °C. Cells in G1 (gap 1), G2 (gap 2), and the S-phase (synthesis), as well as sub-G1 cells, were then quantified using flow cytometry (FL3A). Due to the washing out of small DNA fragments after DNA fragmentation, nuclei with less DNA than G1 (sub-G1) corresponded to apoptotic cells.

Cell death was further quantified by staining cells with Annexin V-fluorescein isothiocyanate (AnnV-FITC) and propidium iodide (PI). After harvesting cells with trypsin/EDTA, they were washed in cold PBS and were resuspended at a concentration of 10^6^ cells/mL in a solution of 10 mM Hepes buffer (pH 7.4), 140 mM NaCl, and 2.5 mM CaCl_2_. To 100 µL of cell suspension, 5 µL of AnnV-FITC solution (BD Biosciences) and 10 µL of PI stock solution (50 μg/mL, Sigma-Aldrich, St. Louis, MO, USA) were added. After 15 min of incubation at room temperature, samples were analyzed by flow cytometry (FACSCalibur, CELLQuest software, version 5.2; Becton Dickinson, Heidelberg, Germany).

### 4.3. Assays for Mitochondrial Membrane Potential and Reactive Oxygen Species (ROS)

Mitochondrial membrane potential (MMP) was quantified by staining cells with the fluorescent dye TMRM^+^ (Tetramethylrhodamin-methylester; Sigma-Aldrich Chemie). Cells grown and treated in 24-well plates were harvested by trypsinization after 4 h or 24 h and were stained at 37 °C for 20 min with 1 µM TMRM^+^. After washing twice with PBS, cells were analyzed via flow cytometry (FL2H).

For microscopic visualization of low MMP, as well as of cell morphological changes over the course of apoptosis, cells were seeded in six-well plates (2 × 10^5^ cells/2 mL) and treated for 6 h or 12 h. Then, cells were stained for 30 min with 2 µg/mL JC-1 (5,5′,6,6′-tetrachloro-1,1′,3,3′-tetraethyl-benzimidazolylcarbocyanin iodide; Life Technologies, Carlsbad, CA, USA) and 0.2 µg/mL Hoechst-33342 (Sigma-Aldrich Chemie). Microscopic images were taken with an Axiovert 200 inverse fluorescence microscope (Carl Zeiss, Jena, Germany) equipped with appropriate fluorescence filter sets and a Hamamatsu ORCA-ER digital camera.

For determination of intracellular ROS levels, cells grown in 24-well plates were pre-incubated for 1 h with the fluorescent dye H_2_DCF-DA (D-399, Thermo Fisher Scientific, Hennigsdorf, Germany, 10 µM) before agonists were applied. At 4 h and 24 h of treatment, cells were trypsinized, washed with PBS, and were analyzed via flow cytometry (FL1H). Treatment with H_2_O_2_ (1 mM, 1 h) was used as a positive control.

### 4.4. Western Blotting

For Western blotting, cells were lysed in a buffer containing 150 mM NaCl, 1 mM EDTA, 1% NP-40, and 50 mM Tris-HCl (pH 8.0), as well as phosphatase and protease inhibitors. Following SDS polyacrylamide gel electrophoresis, proteins were transferred to nitrocellulose membranes (A15033264, GE Healthcare Life Science, Chicago, IL, USA).

The following primary antibodies were used: Cleaved caspase-3 (Cell Signaling, Danvers, MA, USA, 9661, rabbit, 1:1000); Caspase-8 (Cell Signaling, 9746, mouse, 1:1000); Caspase-9 (Cell Signaling, 9502, rabbit, 1:1000); pH2AX (γ-H2AX, Cell Signaling, 80312, mouse, 1:1000); PARP (Cell Signaling, 9542, rabbit, 1:1000); Mcl-1 (Cell Signaling, 4572 and 9429, rabbit, 1:1000); Bcl-2 (Cell Signaling, 15071, mouse, 1:1000); Bcl-w (Cell Signaling, 2724, rabbit, 1:1000); Bcl-x_L_ (Santa Cruz Biotech, Dallas, TX, USA, sc-1690, rabbit, 1:1000); XIAP (Cell Signaling, 2042, rabbit, 1:1000); and GAPDH (Santa Cruz Biotech, sc-32233, mouse, 1:200). Secondary antibodies: peroxidase-labelled goat anti-rabbit and goat anti-mouse (Dako, Hamburg, Germany; 1:5000), as well as rabbit anti-goat (SouthernBiotech, Birmingham, AL, USA, 1:5000).

### 4.5. Statistical Analyses

All statistical analyses were based on at least six values, which were generated either in two series of experiments, each with three independent values, or in three series of experiments, each with two independent values. The two/three values generated in parallel in a single series of experiments trace back to separate cell culture wells that were individually seeded, treated, harvested, and analyzed. For calculation of statistical significance, the Student’s *t*-test was applied after including all individual values, and statistical significance is indicated by asterisks in the figures (* *p* < 0.05).

To recognize possible signs of synergistic effects of drug combinations, the respective combination indices (CI) were calculated using the Compusyn Software 1.0 (ComboSyn, Inc., Paramus, NJ, USA) [94]. Median effect diagrams were plotted as a function of the cell fractions affected by the combinatorial inhibitor treatment. CI values of 1 are indicative of additive effects, while indices of >1 indicate antagonistic effects, and indices of <1 may indicate possible synergistic effects.

Western blot experiments were repeated at least once using two independent series of protein extracts. Signals of Mcl-1 and XIAP were quantified via densitometric analysis and were normalized using the respective GAPDH values. Thus, median induction factors or the percent of downregulation were calculated from two independent experiments.

## 5. Conclusions

BH3 mimetics have shown promising effects in clinical trials for hematological cancers, while solid tumors have appeared to be less responsive. By applying in vitro experiments, we aimed to obtain an overview of the possible suitability of BH3 mimetics for future melanoma therapy. The present study, in agreement with previous studies, clearly showed that the insufficient response of melanoma cells to BH3 mimetics only applies to single treatments, while combination treatments are highly effective. This is underlined here by induction of apoptosis, loss of cell viability, and activation of multiple cell death pathways. The combined inhibition of Mcl-1 and Bcl-2 by ABT-199/S63845 was already effective in most cell lines, but the best response was seen following treatment with ABT-263/S63845, indicating that all four antiapoptotic Bcl-2 proteins (Bcl-2, Bcl-x_L_, Bcl-w, and Mcl-1) should be better targeted. As the combinations were so much more effective, the reported restrictions for the clinical use of BH3 mimetics due to side effects (e.g., platelet inhibition) may also be overcome, because the dose of the single inhibitors may be strongly reduced. Thus, melanoma may be efficiently targeted by BH3 mimetics; the right combinations just have to be selected. These strategies may help to close the presently existing therapeutic gaps in melanoma treatment.

## Data Availability

The data presented in this study are available on request from the corresponding author.

## Supplementary Materials

The supporting information can be downloaded at: https://www.mdpi.com/article/10.3390/ijms25063453/s1.
