# Supplementary material for: Effective Targeting of Melanoma Cells by Combination of Mcl-1 and Bcl-2/Bcl-xL/Bcl-w Inhibitors
Source: Int J Mol Sci. 2024 Mar 19;25(6):3453. doi: 10.3390/ijms25063453 (PMC10970841; doi:10.3390/ijms25063453)
Supplement: Supplementary file 1 [file ijms-25-03453-s001.zip › ijms-2874274-supplementary figures and tables.pdf]

Supplementary figures

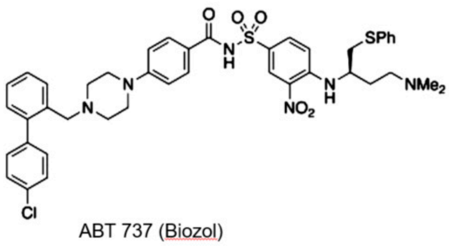

ABT 737 (Biozol)

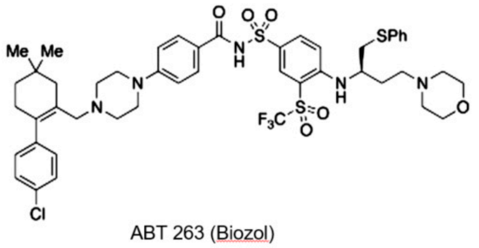

ABT 263 (Biozol)

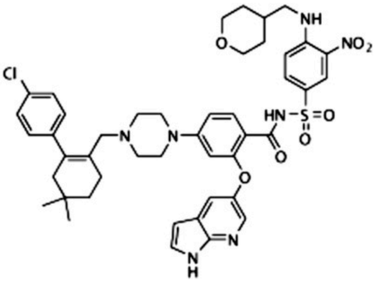

ABT 199 (Selleck Chemicals)

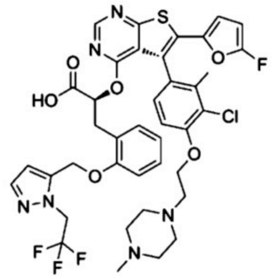

S63845 (MedChemExpress)

Supplementary Figure S1. Chemical structures of the BH3 mimetics used. The Chemical structures of ABT-737, ABT-263, ABT-199 and S63845 are shown. Graphs were taken from the homepages of the companies (Biozol, Selleck Chemicals and MedChemExpress), where the chemicals were purchased.

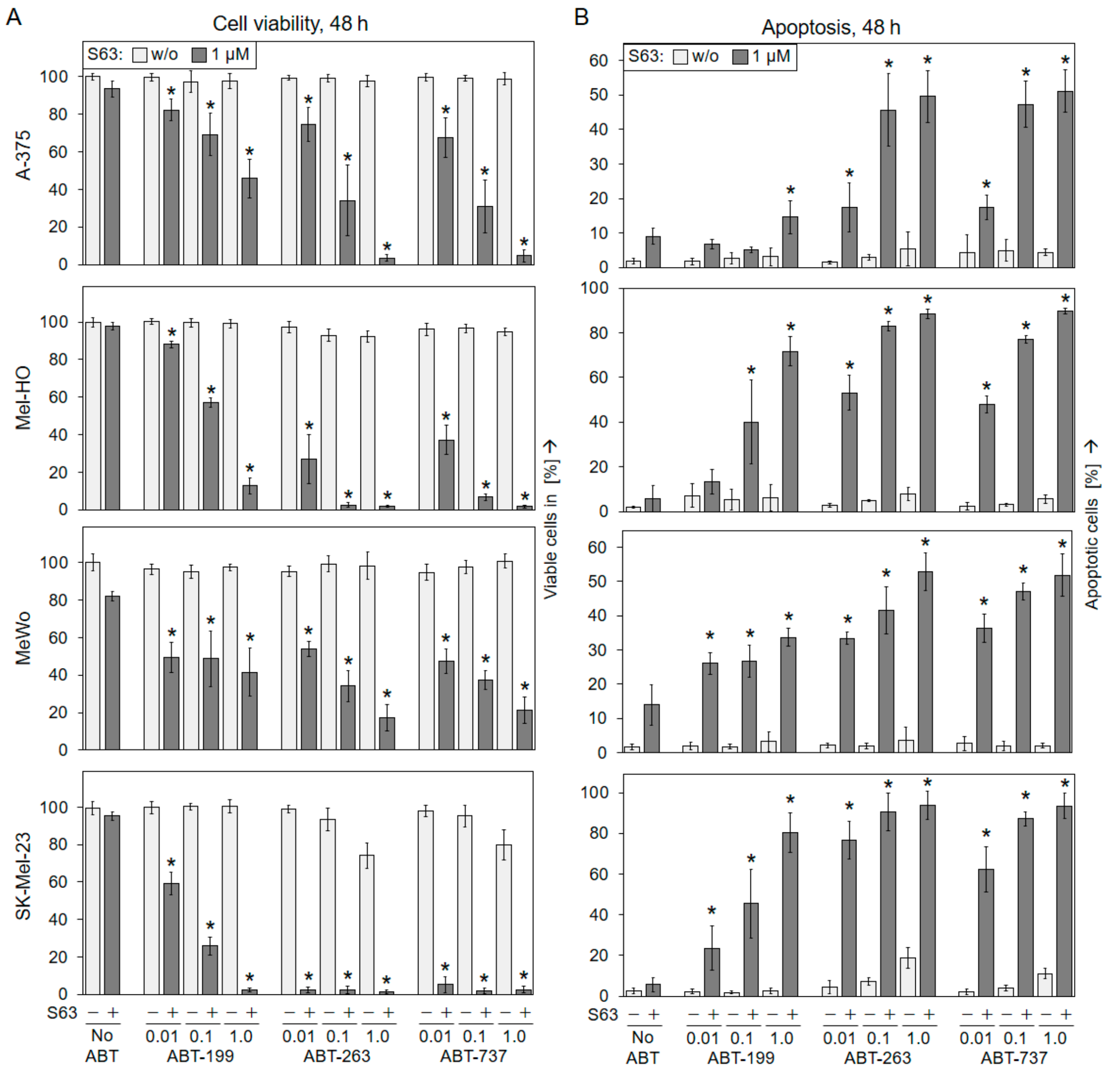

Supplementary Figure S2. Combinations of BH3 mimetics decrease cell viability and induce apoptosis in melanoma cells at 48 h. Melanoma cell lines A-375, Mel-HO, MeWo and SK-Mel-23 were seeded in 24-well plates and were treated with S63845 (S63, 1 µM) as well as with ABT-199, ABT-263 or ABT-737 (0.01, 0.1, 1 µM) as indicated. (**A**) After 48 h, cell viability was determined by calcein-AM staining and flow cytometry. Values represent the percentage of cells with high calcein staining (viable cells). Effects on cell viability are displayed as percentage of non-treated controls (100%). (**B**) Also after 48 h, apoptotic cells were identified as sub-G1 cells in cell cycle analyses by flow cytometry after propidiumiodide staining. (**A**,**B**) At least two series of experiments were performed, each one consisting of independent triplicate values. Mean values of all individual values (at least 6) are shown here. Statistical significance is indicated by asterisks (* $p < 0.05$) and was calculated for the single treatments as compared to non-treated control cells. The combination treatments were compared both to the respective single treatments with ABT (light bars) and to S63845 alone. Largely comparable findings were obtained after 24 h treatments (see Figure 1).

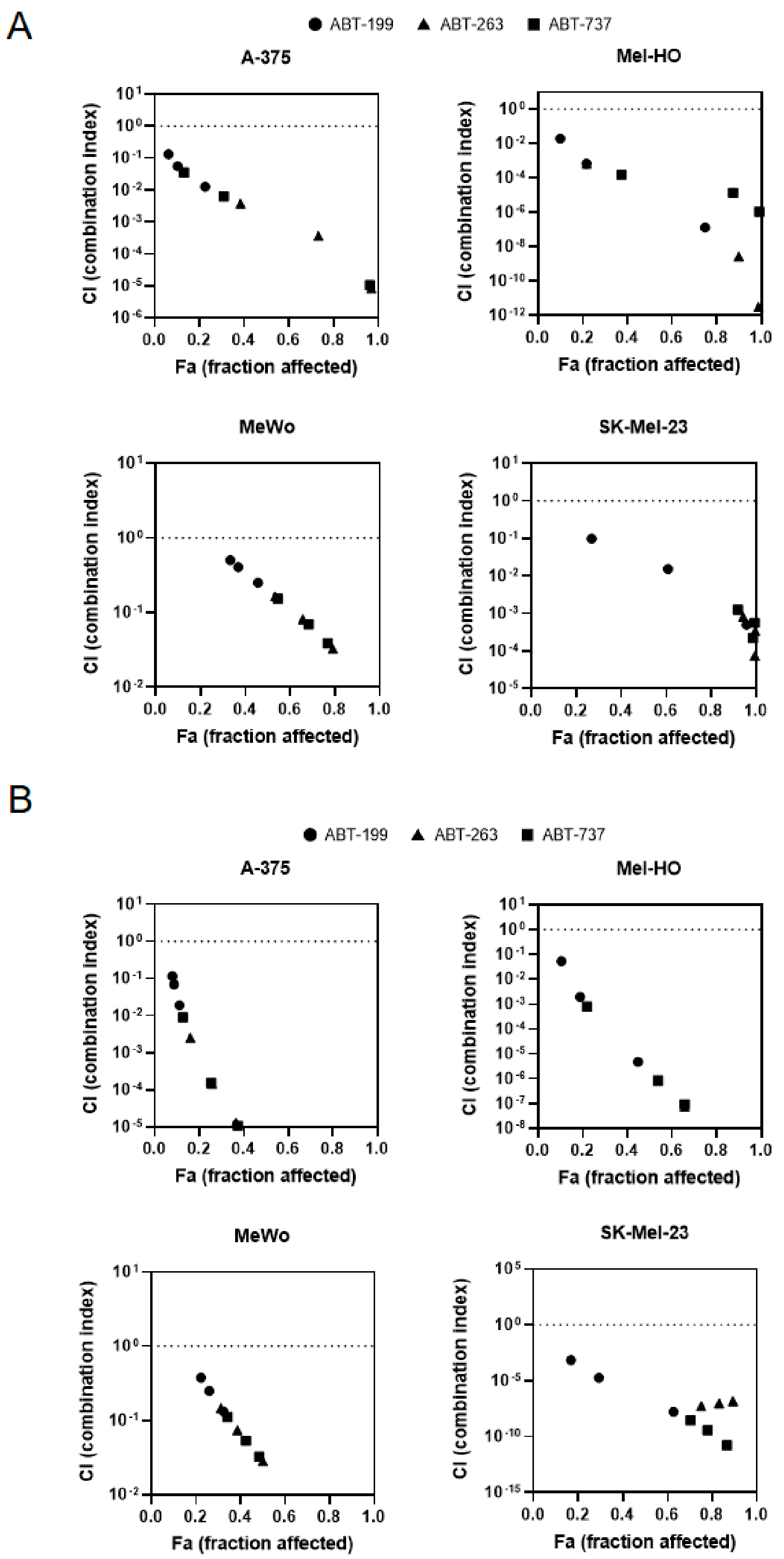

Supplementary Figure S3. Indications of possible synergistic effects by combination of BH3 mimetics. Combintions of S63845 (1 μM) with ABT-199 (circles), ABT-263 (triangles) or ABT-737 (squares) at 0.01, 0.1 and 1 μM were tested for putative synergistic effects on the melanoma cell lines in terms of viability reduction (A) or apoptosis induction (B) at 24 hours. Combination indices (CI) were calculated using CompuSyn software and plotted against the fractions affected (Fa) by the corresponding combination. CI values < 1 possibly

synergistic behavior, CI values > 1 antagonistic behavor; dotted lines at CI = 1 (additive behavior).

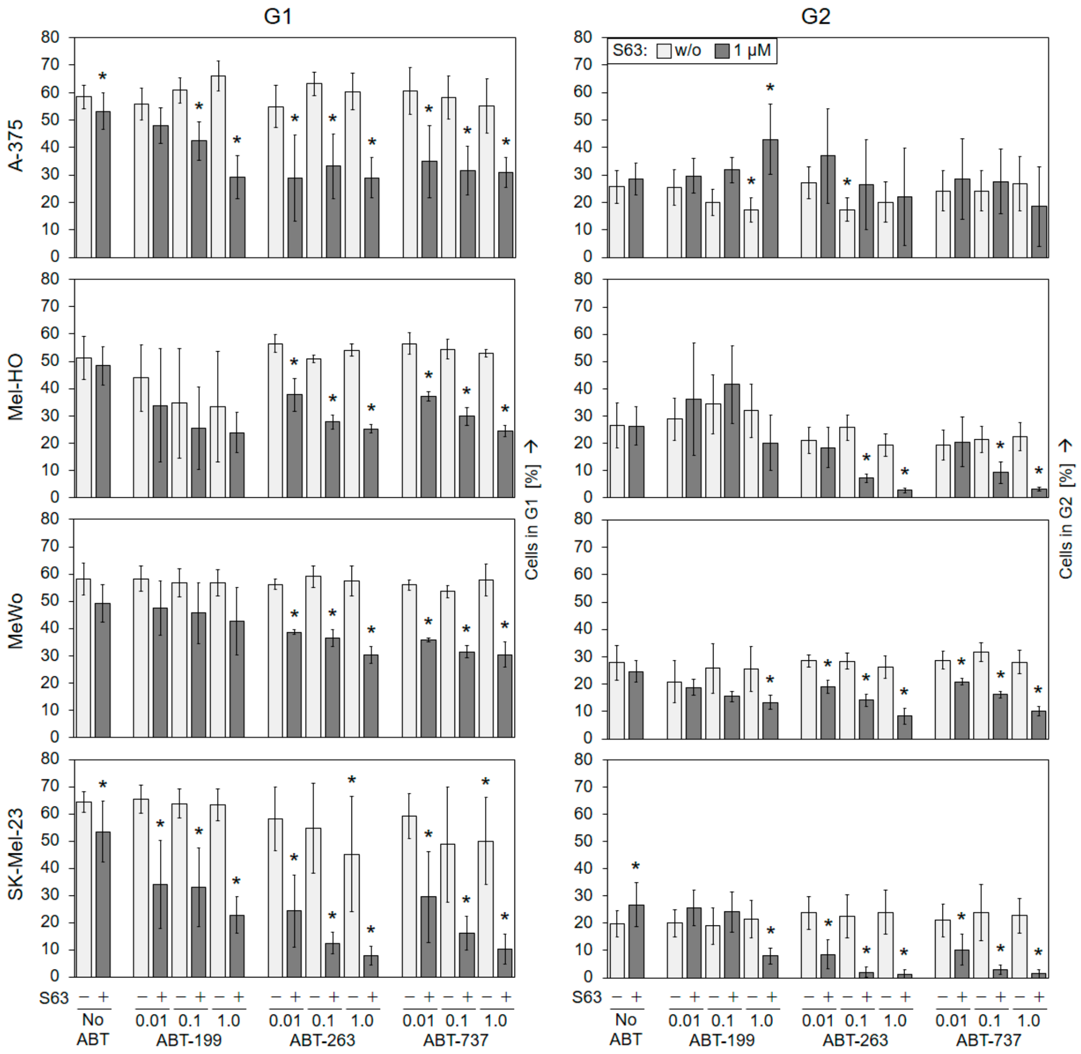

Supplementary Figure S4. Cell cycle analysis after treatment with BH3 mimetics. Quantification of G1 and G2 cell cycle phases was performed after PI staining at 24 h in parallel with quantification of apoptosis (Sub-G1 analysis) shown in Fig 1B. Further information about the protocol can be found there. At least two series of experiments were performed, each one consisting of independent triplicate values. Mean values of all individual values (at least 6) are shown here. Statistical significance is indicated by asterisks (*, $p < 0.05$) and was calculated for the single treatments as compared to non-treated control cells. The combination treatments were compared both to the respective single treatments with ABT (light bars) and to S63845 alone.

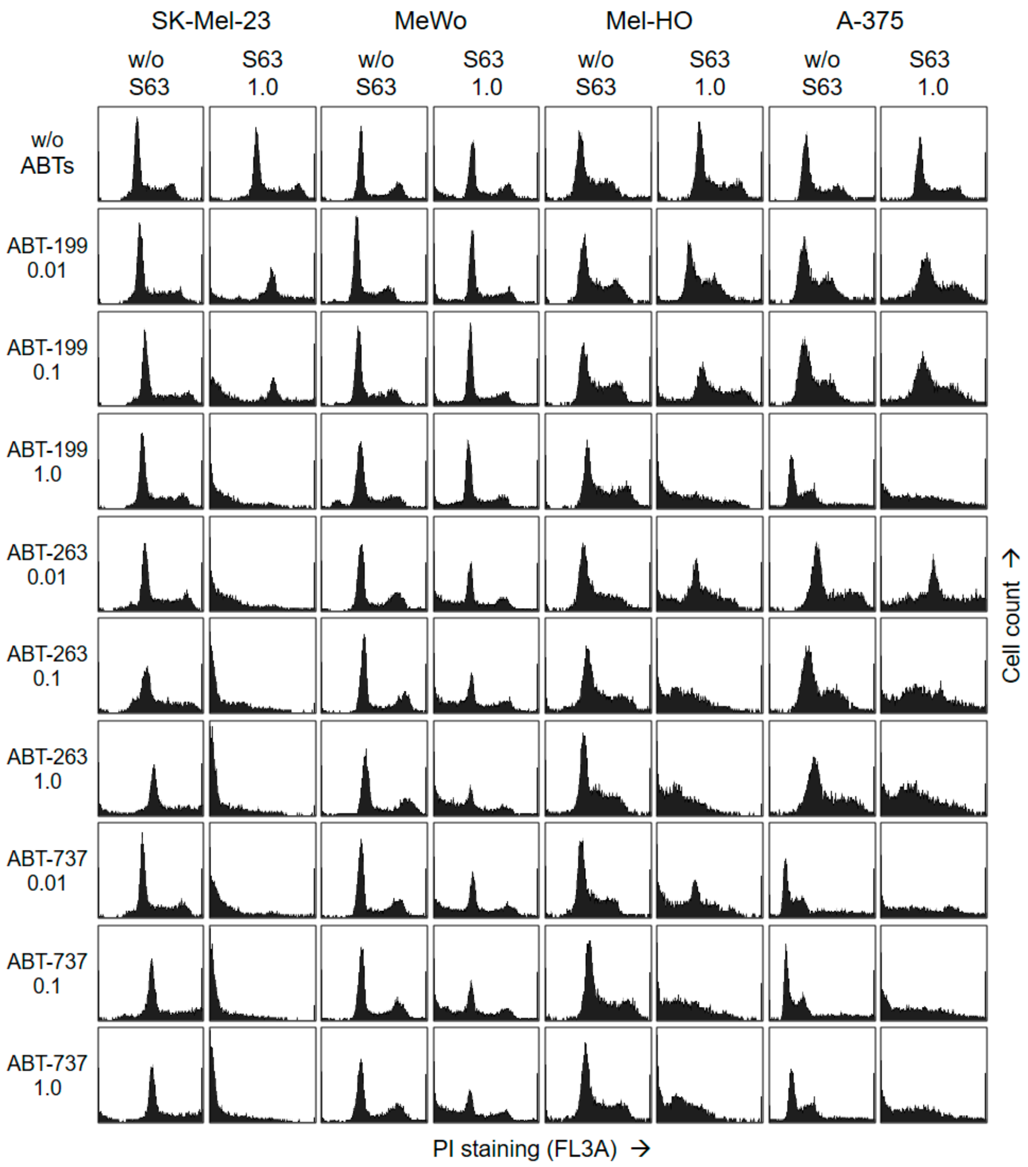

Supplementary Figure S5. Cell cycle profiles after treatment with BH3 mimetics (24 h). In order to complete the information shown in Fig 1B, all cell cycle profiles of four cell lines after single and combination treatments are shown.

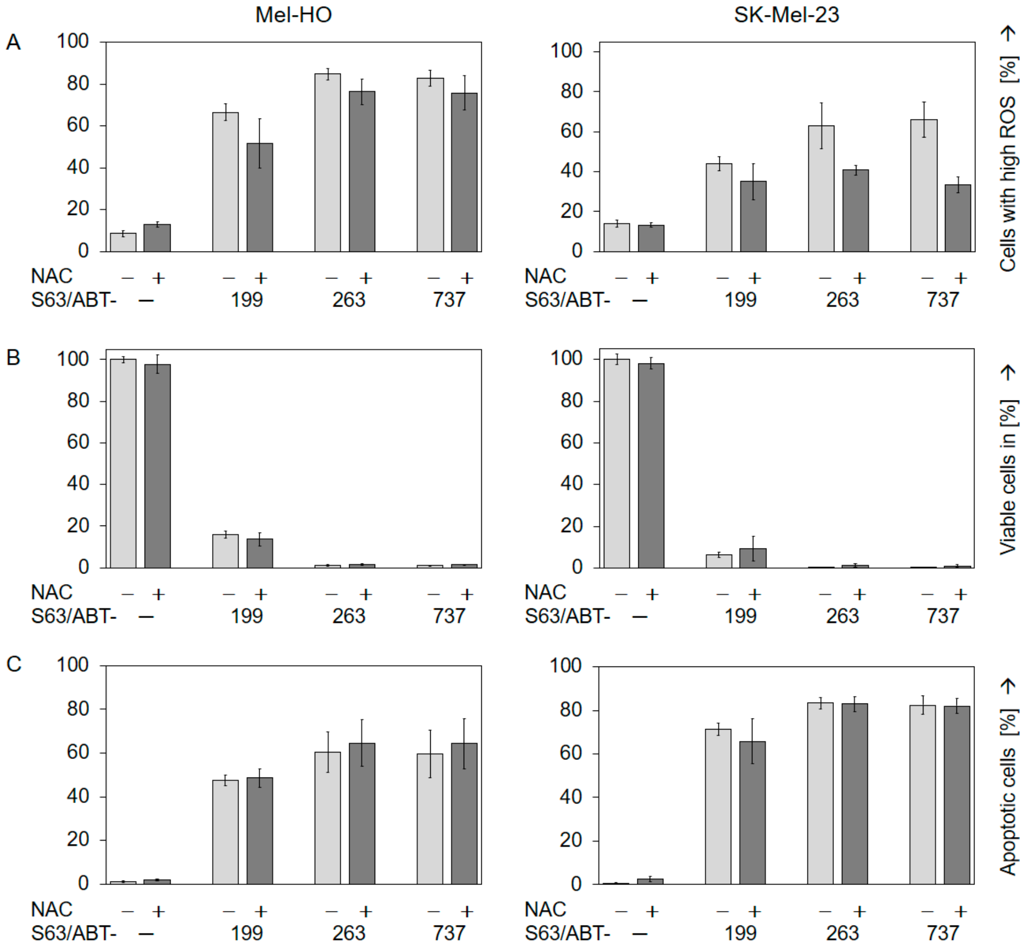

Supplementary Figure S6. No complete reduction of ROS by the antioxidant N-acetylcysteine. Melanoma cell lines Mel-HO and SK-Mel-23 were treated with combinations of BH3 mimetics, as indicated (1 µM concentrations). In addition, cells were pretreated with the antioxidant N-acetylcysteine (NAC, dark grey bars). At 24 h, quantitative assays for ROS (A), cell viability (B) and apoptosis (C) were performed.

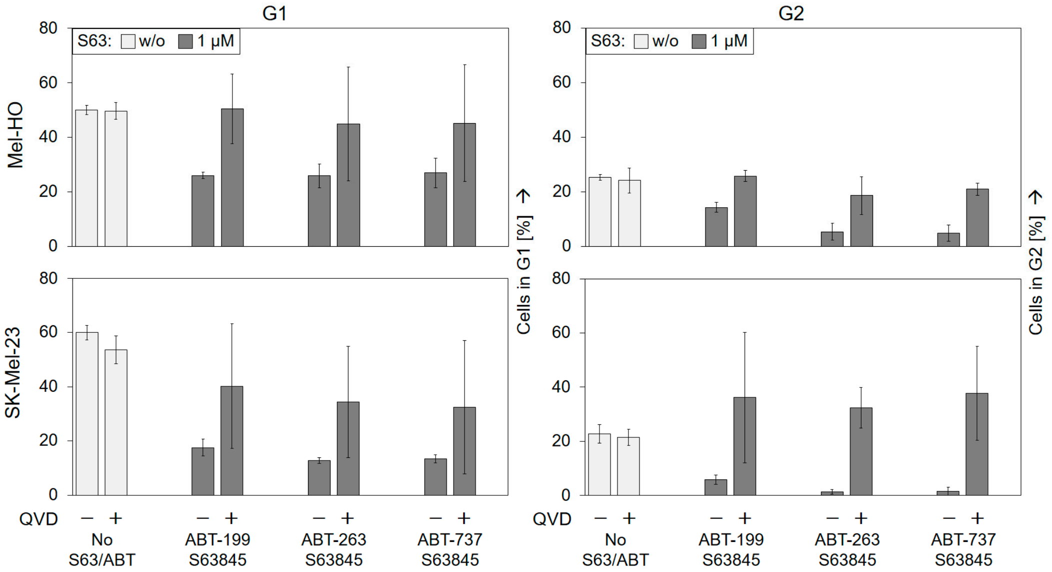

Supplementary Figure S7. G1 and G2 are restored after QVD treatment. Quantification of G1 and G2 cell cycle phases was performed after PI staining in parallel with quantification of apoptosis (Sub-G1 analysis) shown in Fig 6B. Further information about the protocol can be found there.

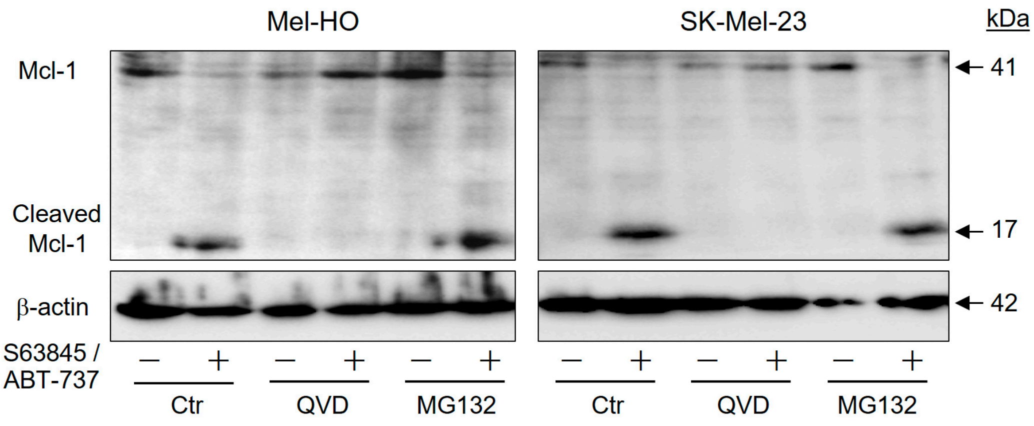

Supplementary Figure S8. Caspase-dependency of Mcl-1 degradation. Mel-HO and SK-Mel-23 cells were treated with the combination of ABT-737 and S63845 (1 μM concentrations, treatment time: 8 h). In addition, cells were treated with the caspase inhibitor QVD-Oph and the proteasome inhibitor MG132, as indicated. Total protein extracts were analyzed by Western blotting for expression of Mcl-1 (41 kD) and its caspase-dependent cleavage product of 17 kD. Beta-actin (42 kD) was analysed as control.

Supplementary Table S1 - All Western blots (8 h) - Quantification - Median values *

		GAPDH	Mcl-1	XIAP	Csp-3	Csp-8	Csp-8	Csp-9	Csp-9	Bcl-2	Bcl-W	Bcl-XL	PARP	H2AX
	kDa	37	41	53	16	57	43/41	47	35	26	18	30	79	15
Mel-HO	Ctr	100	100	100	1,0	100	1,0	100	1,0	100	100	100	1,0	1
	S63	100	144	128	1,3	104	1,1	103	0,9	123	113	93	1,7	5
	ABT-199	100	59	145	2,2	99	1,0	92	1,1	133	133	96	3,1	3
	ABT-199/S63	100	29	116	34,1	77	4,6	72	3,5	148	157	115	16,5	60
	ABT-263	100	29	122	2,3	98	0,9	91	1,1	112	130	118	3,5	8
	ABT-263/S63	100	5	16	40,4	21	3,9	29	2,6	168	139	101	17,2	69
	ABT-737	100	22	100	1,6	79	1,0	76	0,6	97	106	104	2,8	7
	ABT-737/S63	100	4	20	41,8	12	2,5	21	1,3	117	103	76	13,2	52
		GAPDH	Mcl-1	XIAP	Csp-3	Csp-8	Csp-8	Csp-9	Csp-9	Bcl-2	Bcl-W	Bcl-XL	PARP	H2AX
	kDa	37	41	53	16	57	43/41	47	35	26	18	30	79	15
SK-Mel-23	Ctr	100	100	100	1	100	1,0	100	1,0	100	100	100	1,0	1,0
	S63	100	139	88	3	101	1,0	83	1,1	103	106	86	1,1	3,4
	ABT-199	100	86	78	1	106	0,6	77	0,8	121	117	96	0,9	1,0
	ABT-199/S63	100	38	62	141	66	10,3	53	6,1	120	133	101	5,8	64,3
	ABT-263	100	63	87	5	120	1,0	83	1,4	129	138	122	0,6	6,0
	ABT-263/S63	100	10	20	195	17	8,4	21	3,7	141	139	112	6,4	70,3
	ABT-737	100	62	91	5	110	1,1	80	1,4	116	123	107	0,7	11,3
	ABT-737/S63	100	5	15	165	10	4,9	17	2,5	106	89	95	4,2	56,1

* In Supplementary Table S1, the quantification of all Western blot data is summarized. The relative expression values were generated as detailed in the following: 1.) Western blots were quantified by desitometry (each two blots for each protein and cell line, generated from two independent series of protein

extracts). 2.) All values were normalized by their respective GAPDH signals, used as control. 3.) Values of each blot were normalized to the respective non-treated controls, which were set to 100 in case of downregulation or were set to 1.0 in case of an upregulation. 4.) Median values were generated from the two associated blots (two independent series of experiments).
