# Supplementary material for: Effective Targeting of Melanoma Cells by Combination of Mcl-1 and Bcl-2/Bcl-xL/Bcl-w Inhibitors
Source: Int J Mol Sci. 2024 Mar 19;25(6):3453. doi: 10.3390/ijms25063453 (PMC10970841; doi:10.3390/ijms25063453)
Supplement: Supplementary file 1 [file ijms-25-03453-s001.zip › MM Combi BH3 - WB quantified 8h.pptx]

## Slide 1
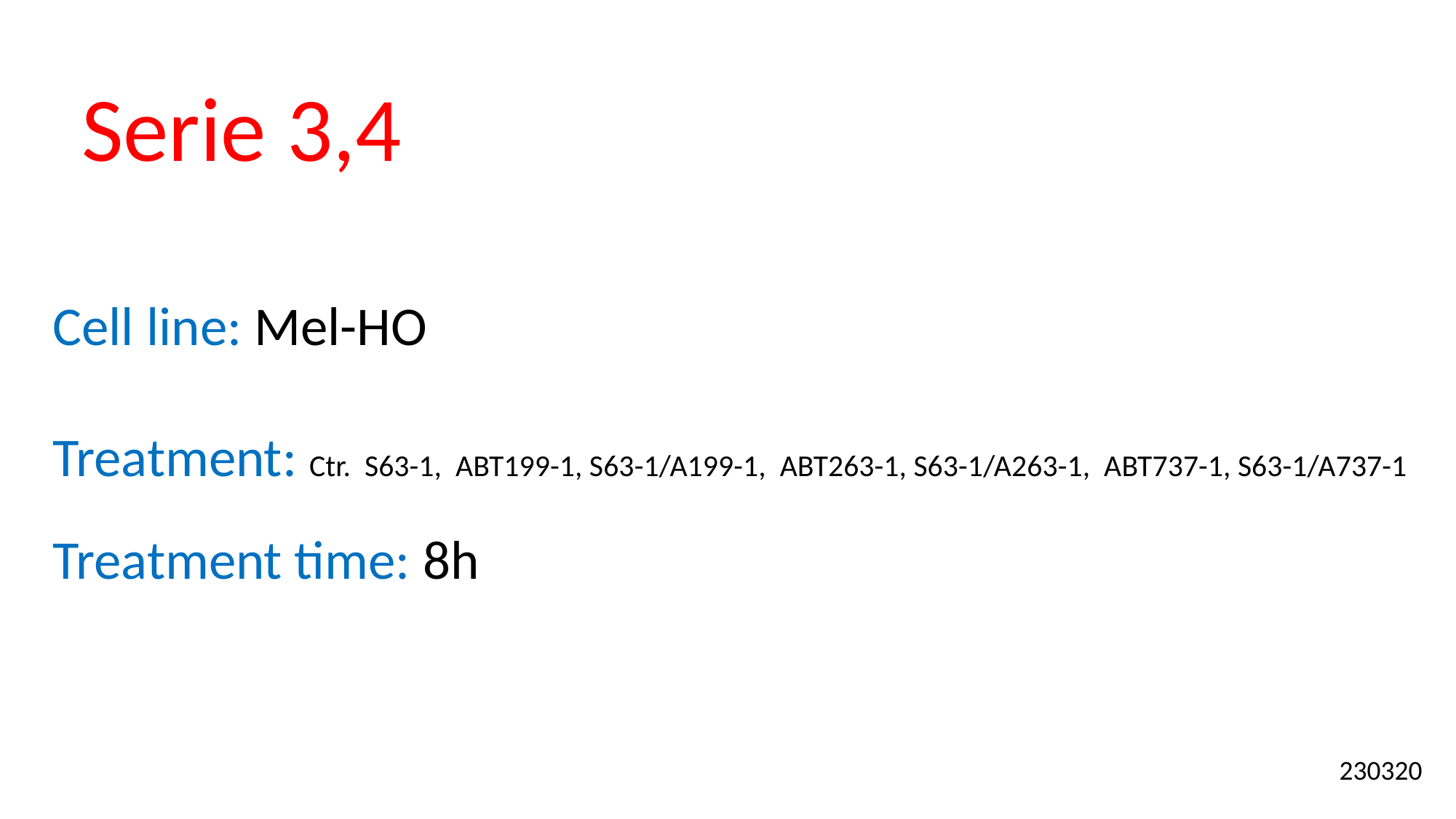

Serie 3,4
Cell line: Mel-HO
Treatment: Ctr. S63-1, ABT199-1, S63-1/A199-1, ABT263-1, S63-1/A263-1, ABT737-1, S63-1/A737-1
Treatment time: 8h
230320

## Slide 2
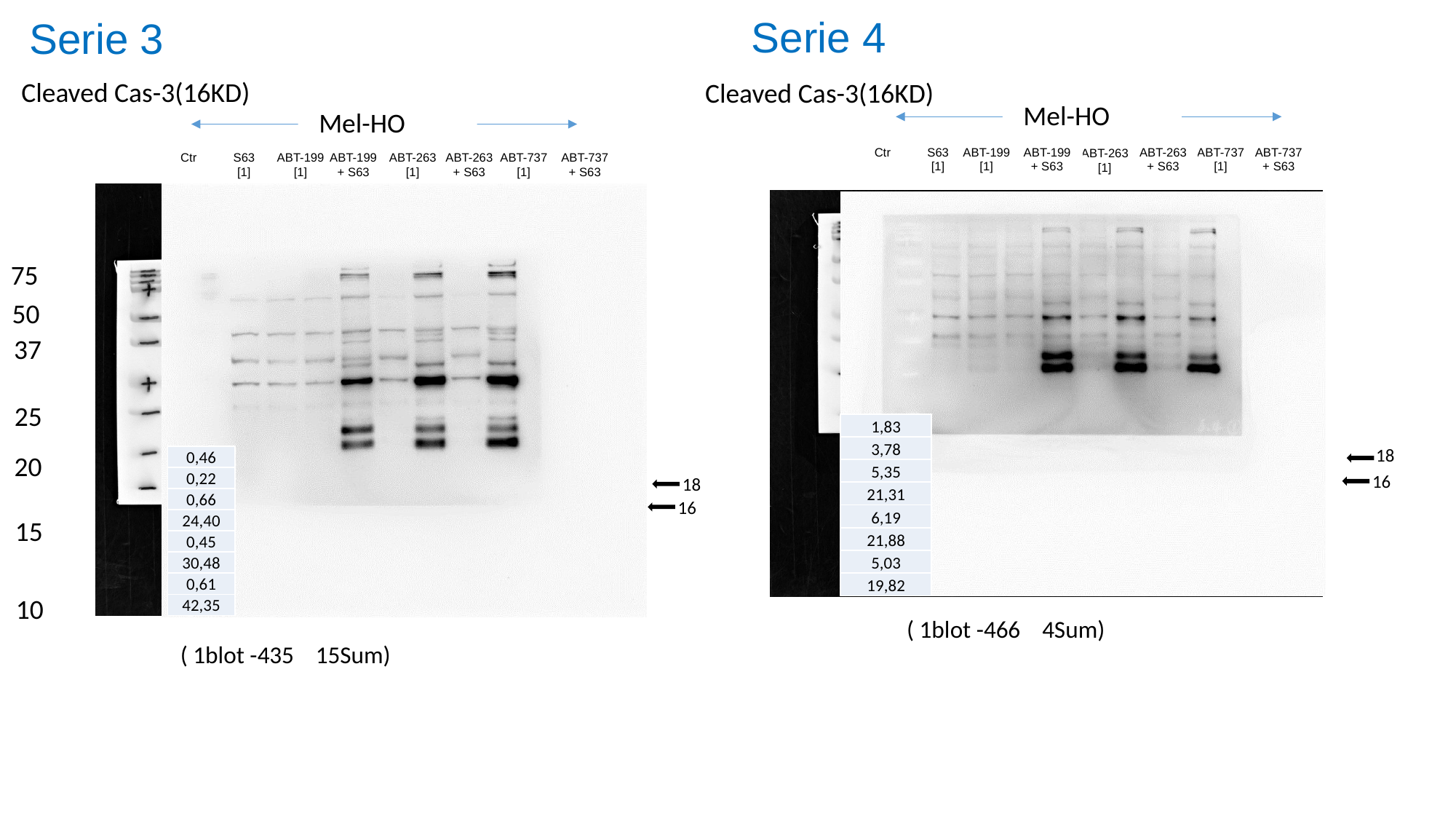

Serie 4
Serie 3
Cleaved Cas-3(16KD)
Cleaved Cas-3(16KD)
Mel-HO
Mel-HO
Ctr
S63
[1]
ABT-199
[1]
ABT-199
+ S63
ABT-263
+ S63
ABT-737
[1]
ABT-737
+ S63
ABT-263
[1]
Ctr
S63
[1]
ABT-199
[1]
ABT-199
+ S63
ABT-263
[1]
ABT-263
+ S63
ABT-737
[1]
ABT-737
+ S63
75
50
37
25
| 1,83 |
| --- |
| 3,78 |
| 5,35 |
| 21,31 |
| 6,19 |
| 21,88 |
| 5,03 |
| 19,82 |
18
20
| 0,46 |
| --- |
| 0,22 |
| 0,66 |
| 24,40 |
| 0,45 |
| 30,48 |
| 0,61 |
| 42,35 |
16
18
16
15
10
 ( 1blot -466 4Sum)
 ( 1blot -435 15Sum)

## Slide 3
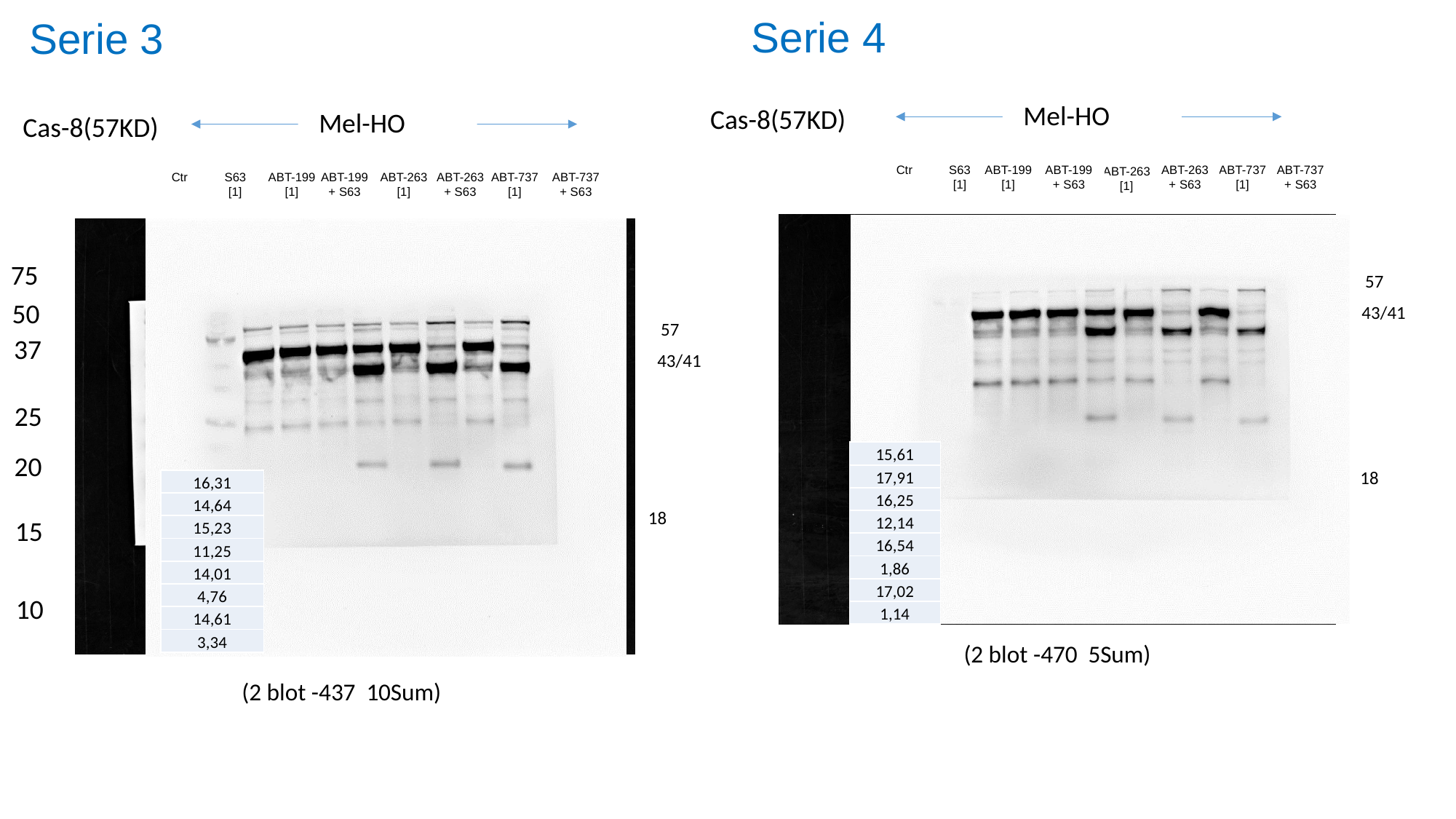

Serie 4
Serie 3
Mel-HO
Cas-8(57KD)
Mel-HO
Cas-8(57KD)
Ctr
S63
[1]
ABT-199
[1]
ABT-199
+ S63
ABT-263
+ S63
ABT-737
[1]
ABT-737
+ S63
ABT-263
[1]
Ctr
S63
[1]
ABT-199
[1]
ABT-199
+ S63
ABT-263
[1]
ABT-263
+ S63
ABT-737
[1]
ABT-737
+ S63
75
57
50
43/41
57
37
43/41
25
| 15,61 |
| --- |
| 17,91 |
| 16,25 |
| 12,14 |
| 16,54 |
| 1,86 |
| 17,02 |
| 1,14 |
20
18
| 16,31 |
| --- |
| 14,64 |
| 15,23 |
| 11,25 |
| 14,01 |
| 4,76 |
| 14,61 |
| 3,34 |
18
15
10
 (2 blot -470 5Sum)
 (2 blot -437 10Sum)

## Slide 4
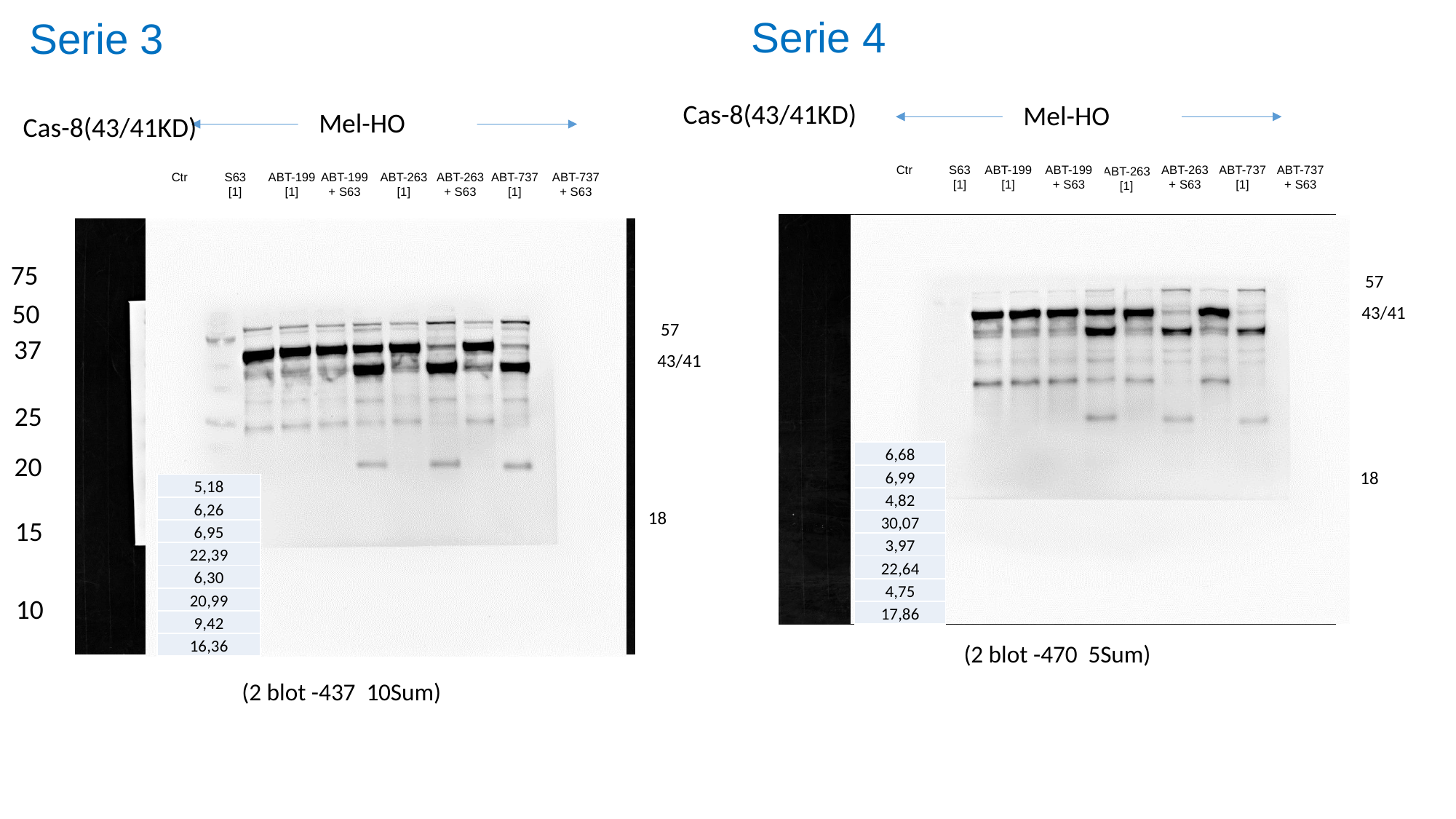

Serie 4
Serie 3
Cas-8(43/41KD)
Mel-HO
Mel-HO
Cas-8(43/41KD)
Ctr
S63
[1]
ABT-199
[1]
ABT-199
+ S63
ABT-263
+ S63
ABT-737
[1]
ABT-737
+ S63
ABT-263
[1]
Ctr
S63
[1]
ABT-199
[1]
ABT-199
+ S63
ABT-263
[1]
ABT-263
+ S63
ABT-737
[1]
ABT-737
+ S63
75
57
50
43/41
57
37
43/41
25
| 6,68 |
| --- |
| 6,99 |
| 4,82 |
| 30,07 |
| 3,97 |
| 22,64 |
| 4,75 |
| 17,86 |
20
18
| 5,18 |
| --- |
| 6,26 |
| 6,95 |
| 22,39 |
| 6,30 |
| 20,99 |
| 9,42 |
| 16,36 |
18
15
10
 (2 blot -470 5Sum)
 (2 blot -437 10Sum)

## Slide 5
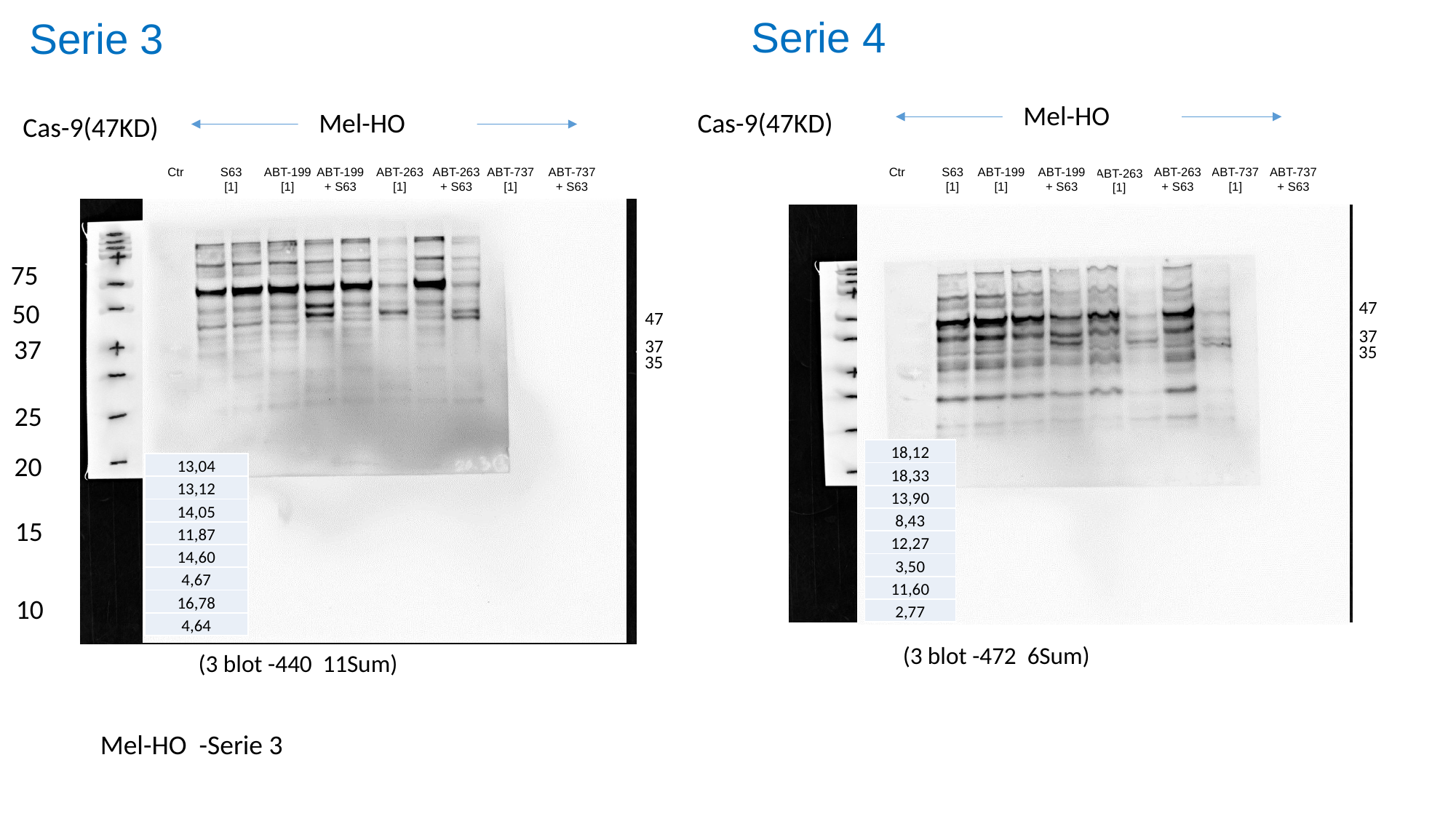

Serie 4
Serie 3
Mel-HO
Mel-HO
Cas-9(47KD)
Cas-9(47KD)
Ctr
S63
[1]
ABT-199
[1]
ABT-199
+ S63
ABT-263
+ S63
ABT-737
[1]
ABT-737
+ S63
ABT-263
[1]
Ctr
S63
[1]
ABT-199
[1]
ABT-199
+ S63
ABT-263
[1]
ABT-263
+ S63
ABT-737
[1]
ABT-737
+ S63
75
47
50
47
37
37
37
35
35
25
| 18,12 |
| --- |
| 18,33 |
| 13,90 |
| 8,43 |
| 12,27 |
| 3,50 |
| 11,60 |
| 2,77 |
20
| 13,04 |
| --- |
| 13,12 |
| 14,05 |
| 11,87 |
| 14,60 |
| 4,67 |
| 16,78 |
| 4,64 |
15
10
 (3 blot -472 6Sum)
 (3 blot -440 11Sum)
Mel-HO -Serie 3

## Slide 6
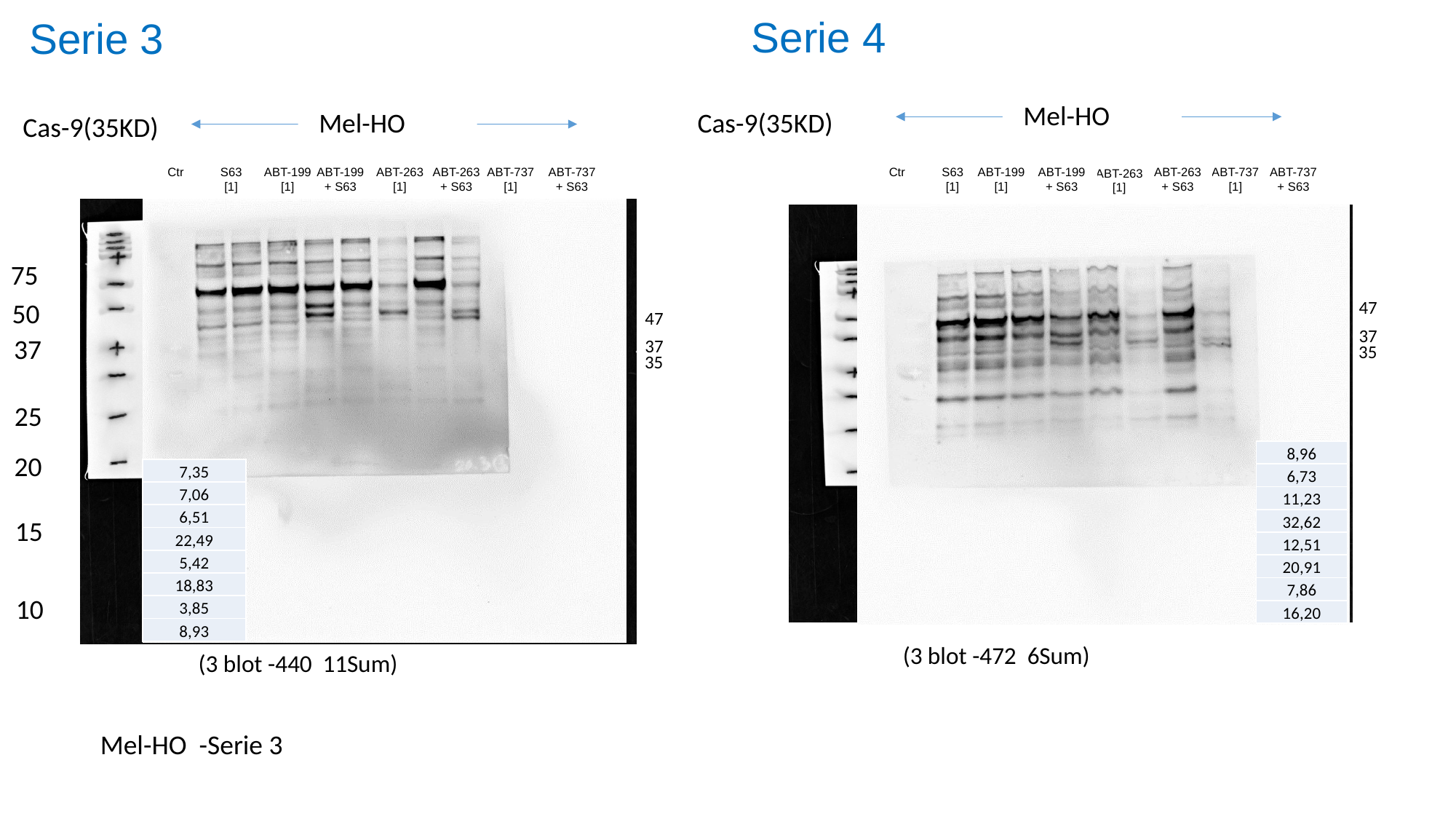

Serie 4
Serie 3
Mel-HO
Mel-HO
Cas-9(35KD)
Cas-9(35KD)
Ctr
S63
[1]
ABT-199
[1]
ABT-199
+ S63
ABT-263
+ S63
ABT-737
[1]
ABT-737
+ S63
ABT-263
[1]
Ctr
S63
[1]
ABT-199
[1]
ABT-199
+ S63
ABT-263
[1]
ABT-263
+ S63
ABT-737
[1]
ABT-737
+ S63
75
47
50
47
37
37
37
35
35
25
| 8,96 |
| --- |
| 6,73 |
| 11,23 |
| 32,62 |
| 12,51 |
| 20,91 |
| 7,86 |
| 16,20 |
20
| 7,35 |
| --- |
| 7,06 |
| 6,51 |
| 22,49 |
| 5,42 |
| 18,83 |
| 3,85 |
| 8,93 |
15
10
 (3 blot -472 6Sum)
 (3 blot -440 11Sum)
Mel-HO -Serie 3

## Slide 7
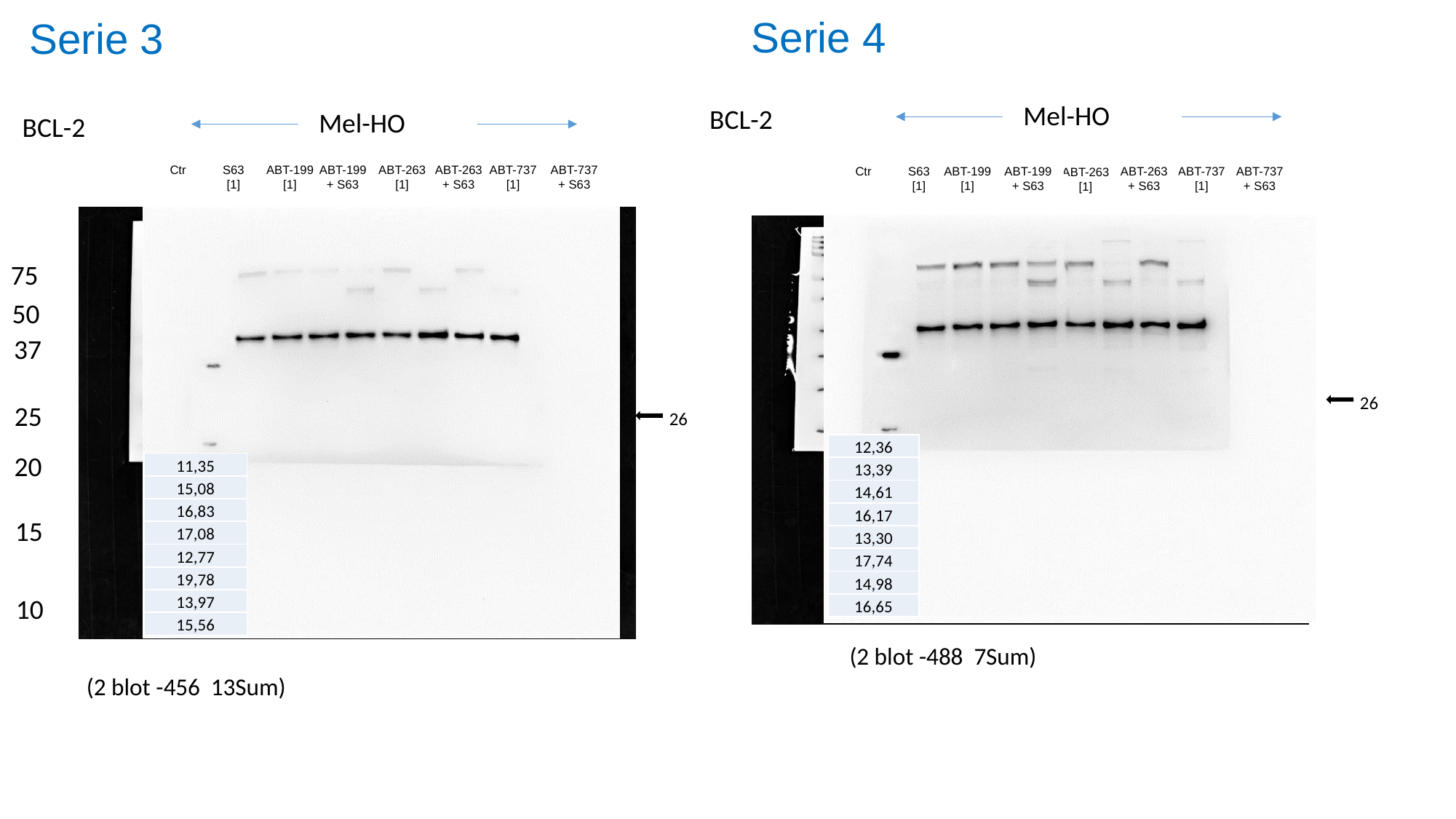

Serie 4
Serie 3
Mel-HO
BCL-2
Mel-HO
BCL-2
Ctr
S63
[1]
ABT-199
[1]
ABT-199
+ S63
ABT-263
[1]
ABT-263
+ S63
ABT-737
[1]
ABT-737
+ S63
Ctr
S63
[1]
ABT-199
[1]
ABT-199
+ S63
ABT-263
+ S63
ABT-737
[1]
ABT-737
+ S63
ABT-263
[1]
75
50
37
26
25
26
| 12,36 |
| --- |
| 13,39 |
| 14,61 |
| 16,17 |
| 13,30 |
| 17,74 |
| 14,98 |
| 16,65 |
20
| 11,35 |
| --- |
| 15,08 |
| 16,83 |
| 17,08 |
| 12,77 |
| 19,78 |
| 13,97 |
| 15,56 |
15
10
 (2 blot -488 7Sum)
 (2 blot -456 13Sum)

## Slide 8
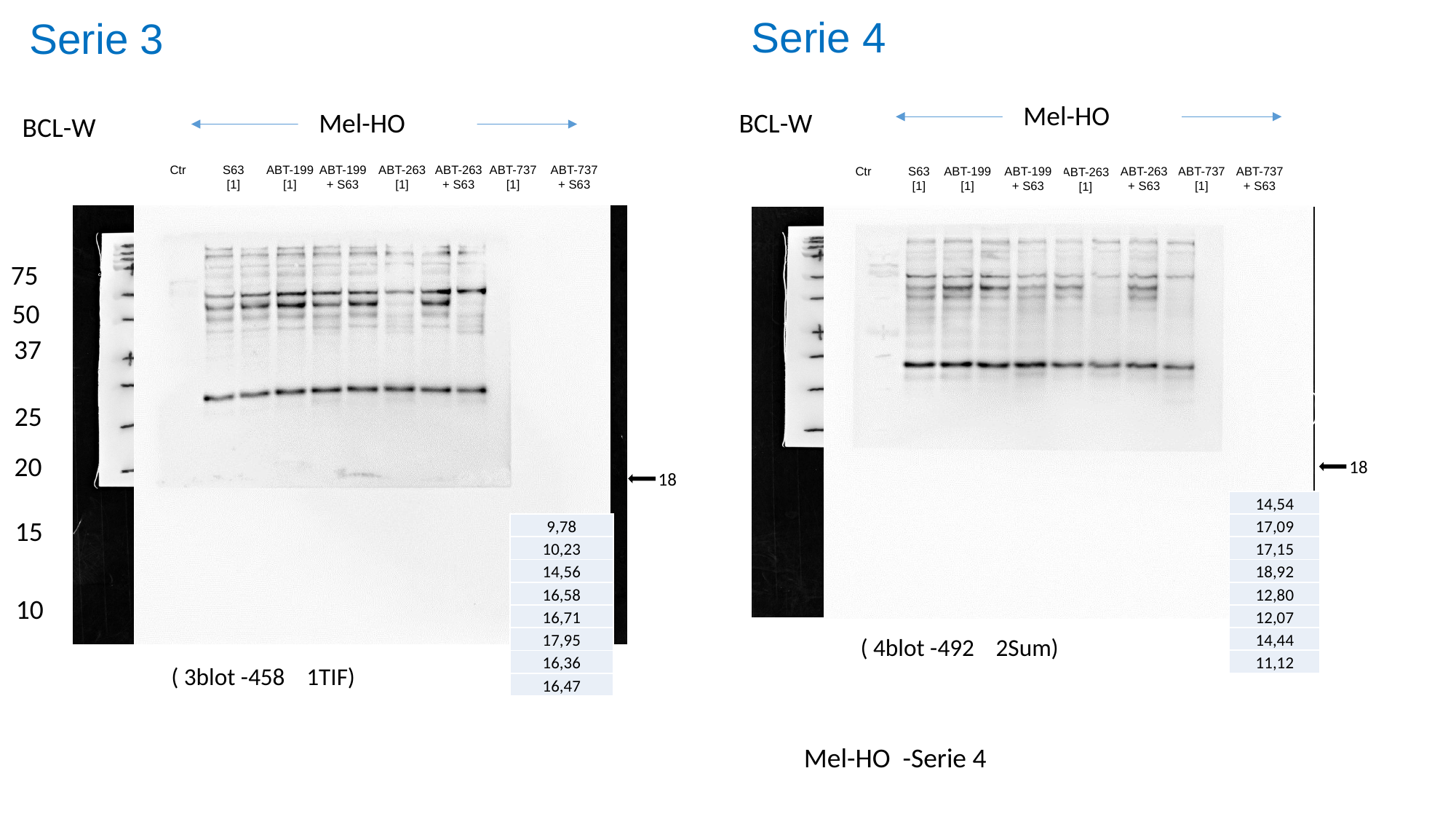

Serie 4
Serie 3
Mel-HO
Mel-HO
BCL-W
BCL-W
Ctr
S63
[1]
ABT-199
[1]
ABT-199
+ S63
ABT-263
[1]
ABT-263
+ S63
ABT-737
[1]
ABT-737
+ S63
Ctr
S63
[1]
ABT-199
[1]
ABT-199
+ S63
ABT-263
+ S63
ABT-737
[1]
ABT-737
+ S63
ABT-263
[1]
75
50
37
25
20
18
18
| 14,54 |
| --- |
| 17,09 |
| 17,15 |
| 18,92 |
| 12,80 |
| 12,07 |
| 14,44 |
| 11,12 |
15
| 9,78 |
| --- |
| 10,23 |
| 14,56 |
| 16,58 |
| 16,71 |
| 17,95 |
| 16,36 |
| 16,47 |
10
 ( 4blot -492 2Sum)
 ( 3blot -458 1TIF)
Mel-HO -Serie 4

## Slide 9
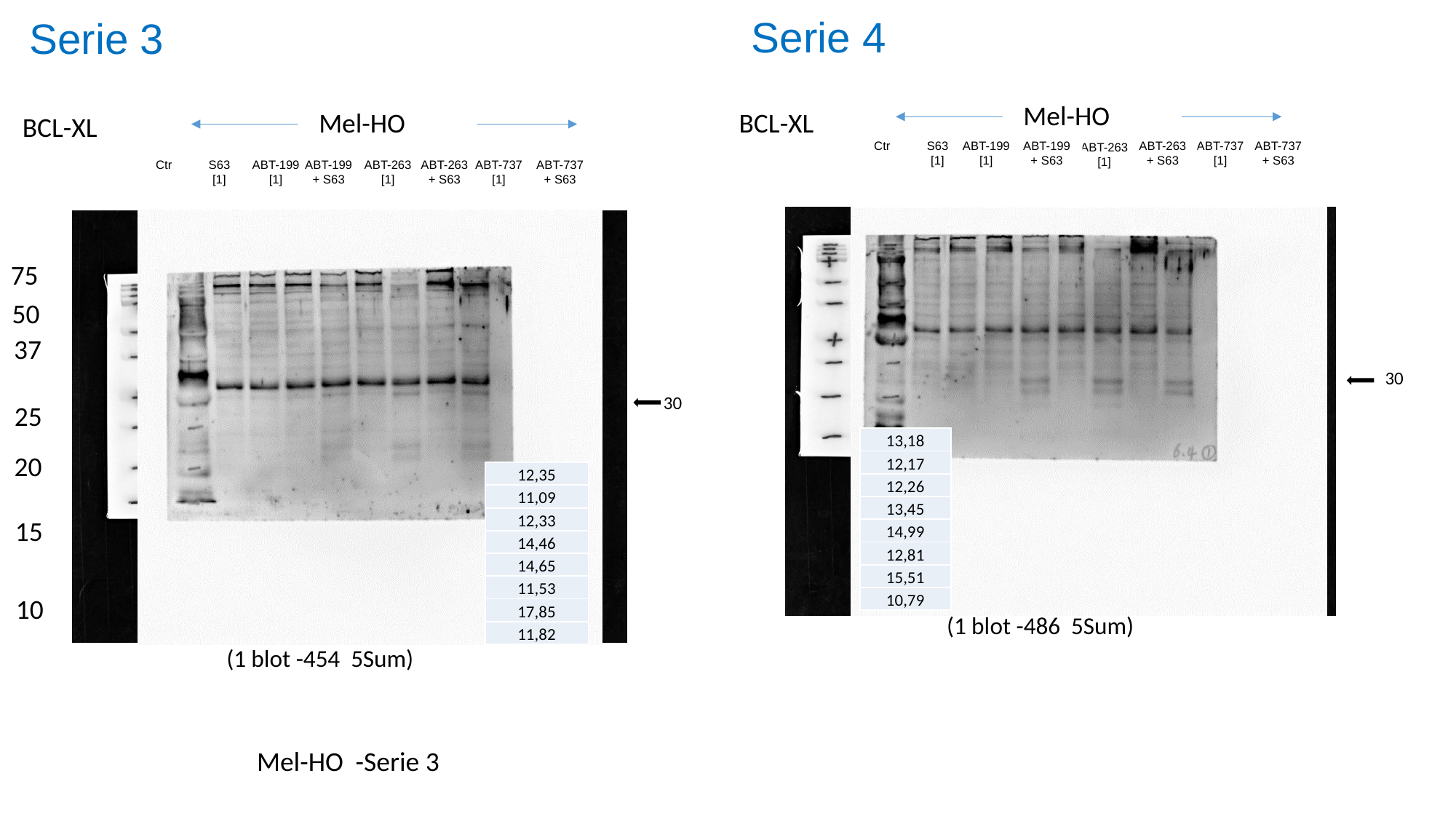

Serie 4
Serie 3
Mel-HO
Mel-HO
BCL-XL
BCL-XL
Ctr
S63
[1]
ABT-199
[1]
ABT-199
+ S63
ABT-263
+ S63
ABT-737
[1]
ABT-737
+ S63
ABT-263
[1]
Ctr
S63
[1]
ABT-199
[1]
ABT-199
+ S63
ABT-263
[1]
ABT-263
+ S63
ABT-737
[1]
ABT-737
+ S63
75
50
37
30
30
25
| 13,18 |
| --- |
| 12,17 |
| 12,26 |
| 13,45 |
| 14,99 |
| 12,81 |
| 15,51 |
| 10,79 |
20
| 12,35 |
| --- |
| 11,09 |
| 12,33 |
| 14,46 |
| 14,65 |
| 11,53 |
| 17,85 |
| 11,82 |
15
10
 (1 blot -486 5Sum)
 (1 blot -454 5Sum)
Mel-HO -Serie 3

## Slide 10
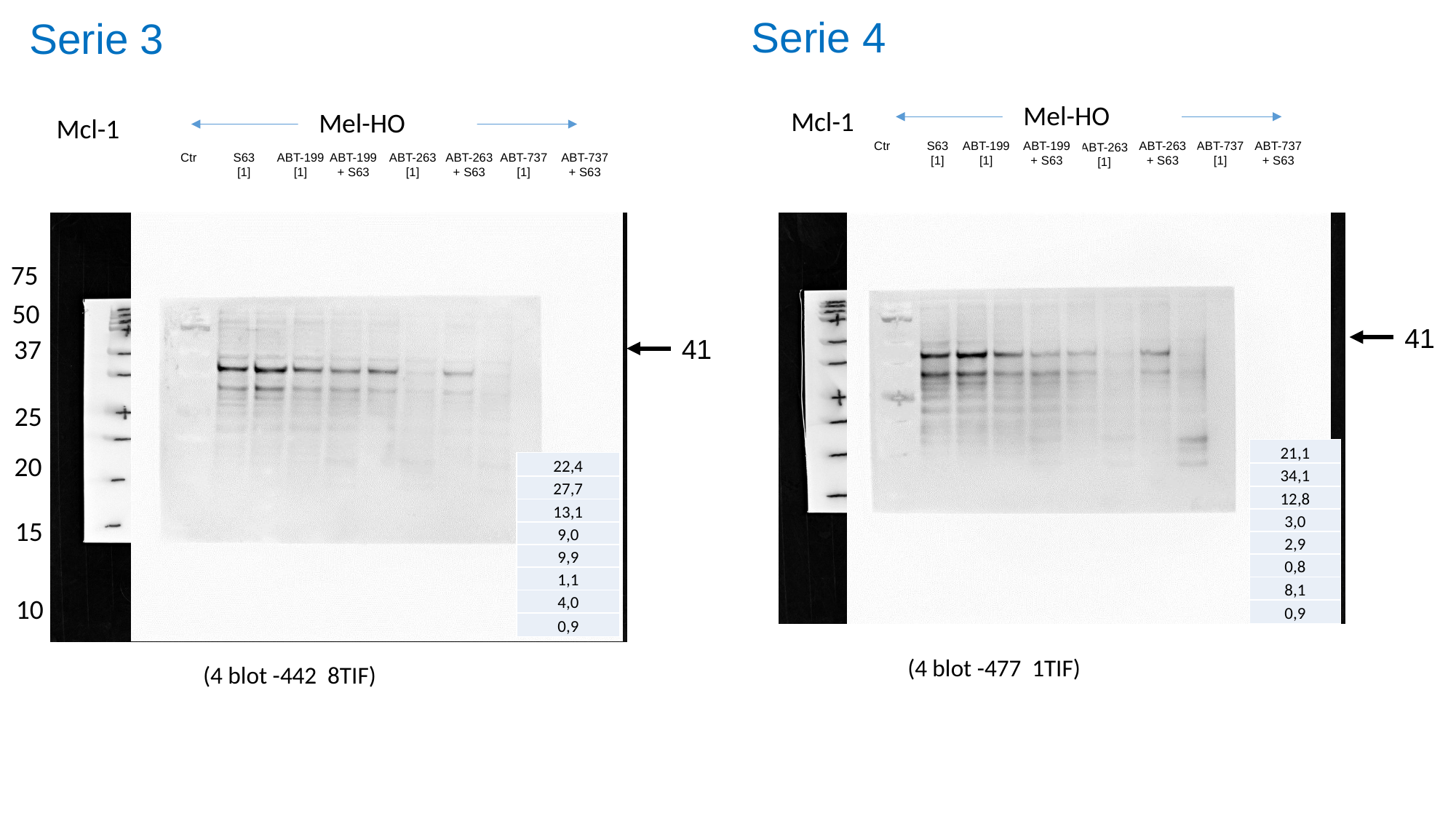

Serie 4
Serie 3
Mel-HO
Mcl-1
Mel-HO
Mcl-1
Ctr
S63
[1]
ABT-199
[1]
ABT-199
+ S63
ABT-263
+ S63
ABT-737
[1]
ABT-737
+ S63
ABT-263
[1]
Ctr
S63
[1]
ABT-199
[1]
ABT-199
+ S63
ABT-263
[1]
ABT-263
+ S63
ABT-737
[1]
ABT-737
+ S63
75
50
41
37
41
25
| 21,1 |
| --- |
| 34,1 |
| 12,8 |
| 3,0 |
| 2,9 |
| 0,8 |
| 8,1 |
| 0,9 |
20
| 22,4 |
| --- |
| 27,7 |
| 13,1 |
| 9,0 |
| 9,9 |
| 1,1 |
| 4,0 |
| 0,9 |
15
10
 (4 blot -477 1TIF)
 (4 blot -442 8TIF)

## Slide 11
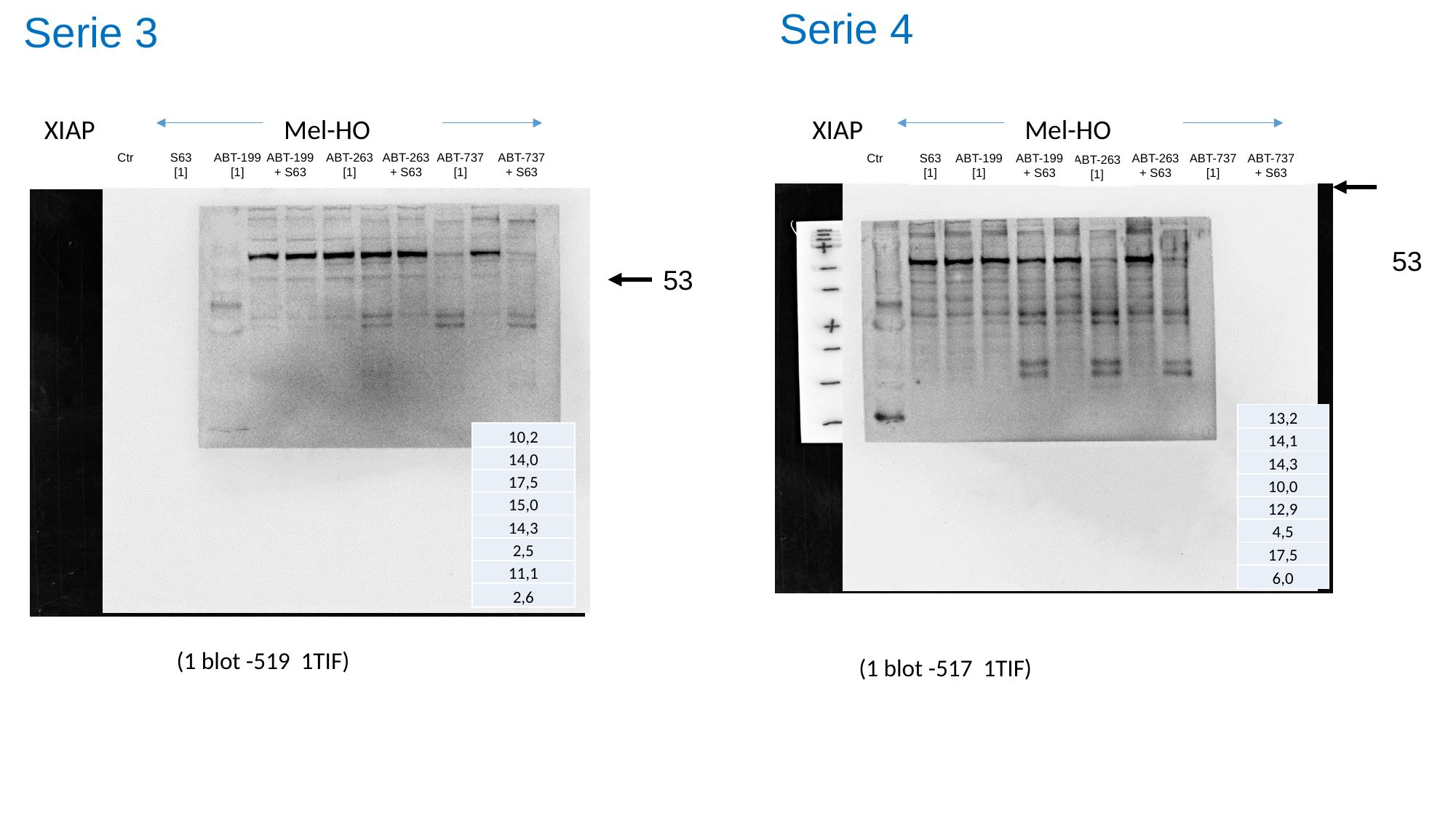

Serie 3
18
16
Serie 4
XIAP
Mel-HO
 XIAP
Mel-HO
Ctr
S63
[1]
ABT-199
[1]
ABT-199
+ S63
ABT-263
[1]
ABT-263
+ S63
ABT-737
[1]
ABT-737
+ S63
Ctr
S63
[1]
ABT-199
[1]
ABT-199
+ S63
ABT-263
+ S63
ABT-737
[1]
ABT-737
+ S63
ABT-263
[1]
53
53
| 13,2 |
| --- |
| 14,1 |
| 14,3 |
| 10,0 |
| 12,9 |
| 4,5 |
| 17,5 |
| 6,0 |
| 10,2 |
| --- |
| 14,0 |
| 17,5 |
| 15,0 |
| 14,3 |
| 2,5 |
| 11,1 |
| 2,6 |
 (1 blot -519 1TIF)
 (1 blot -517 1TIF)

## Slide 12
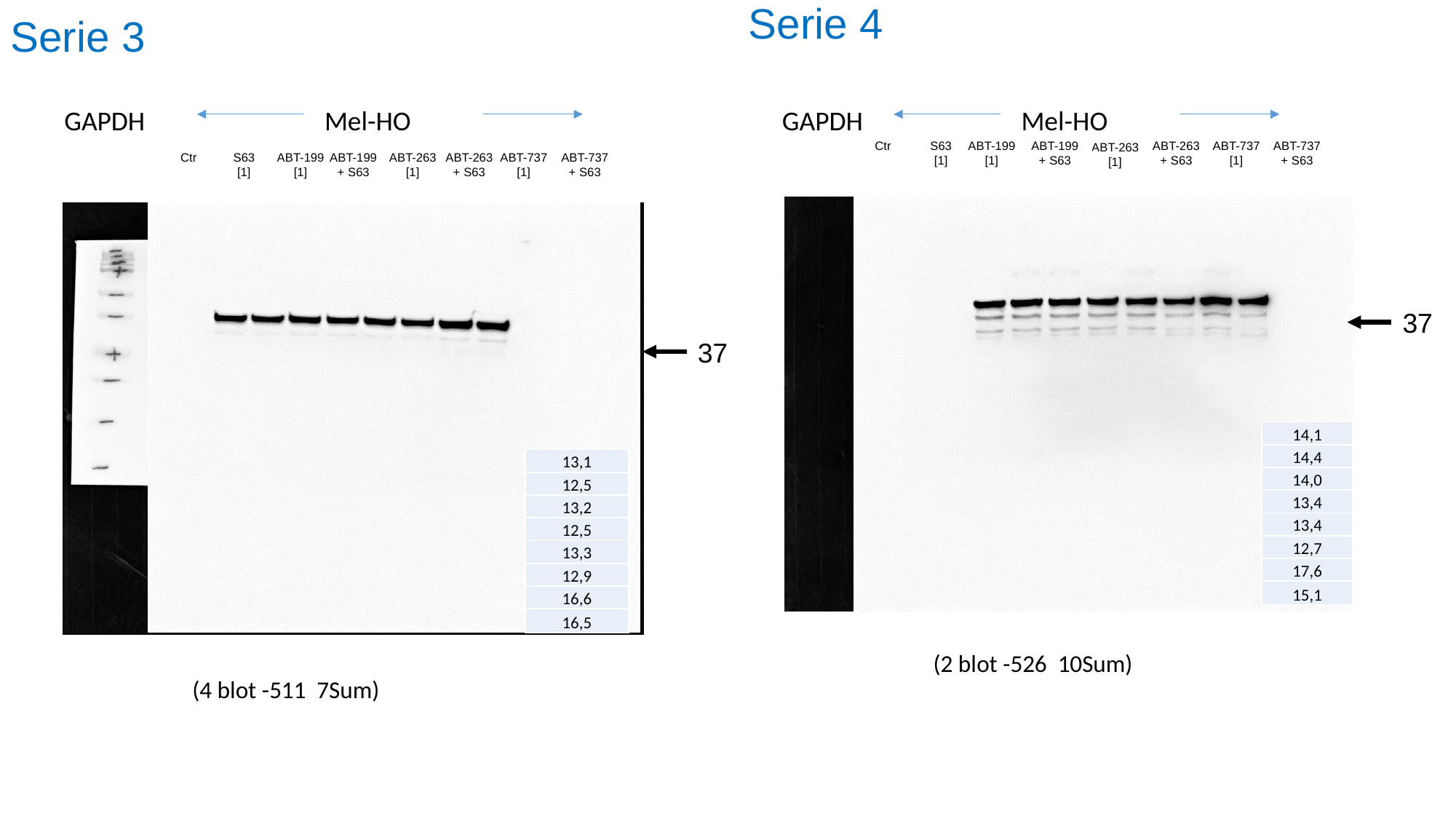

18
16
Serie 4
Serie 3
 GAPDH
Mel-HO
 GAPDH
Mel-HO
Ctr
S63
[1]
ABT-199
[1]
ABT-199
+ S63
ABT-263
+ S63
ABT-737
[1]
ABT-737
+ S63
ABT-263
[1]
Ctr
S63
[1]
ABT-199
[1]
ABT-199
+ S63
ABT-263
[1]
ABT-263
+ S63
ABT-737
[1]
ABT-737
+ S63
37
37
| 14,1 |
| --- |
| 14,4 |
| 14,0 |
| 13,4 |
| 13,4 |
| 12,7 |
| 17,6 |
| 15,1 |
| 13,1 |
| --- |
| 12,5 |
| 13,2 |
| 12,5 |
| 13,3 |
| 12,9 |
| 16,6 |
| 16,5 |
 (2 blot -526 10Sum)
 (4 blot -511 7Sum)

## Slide 13
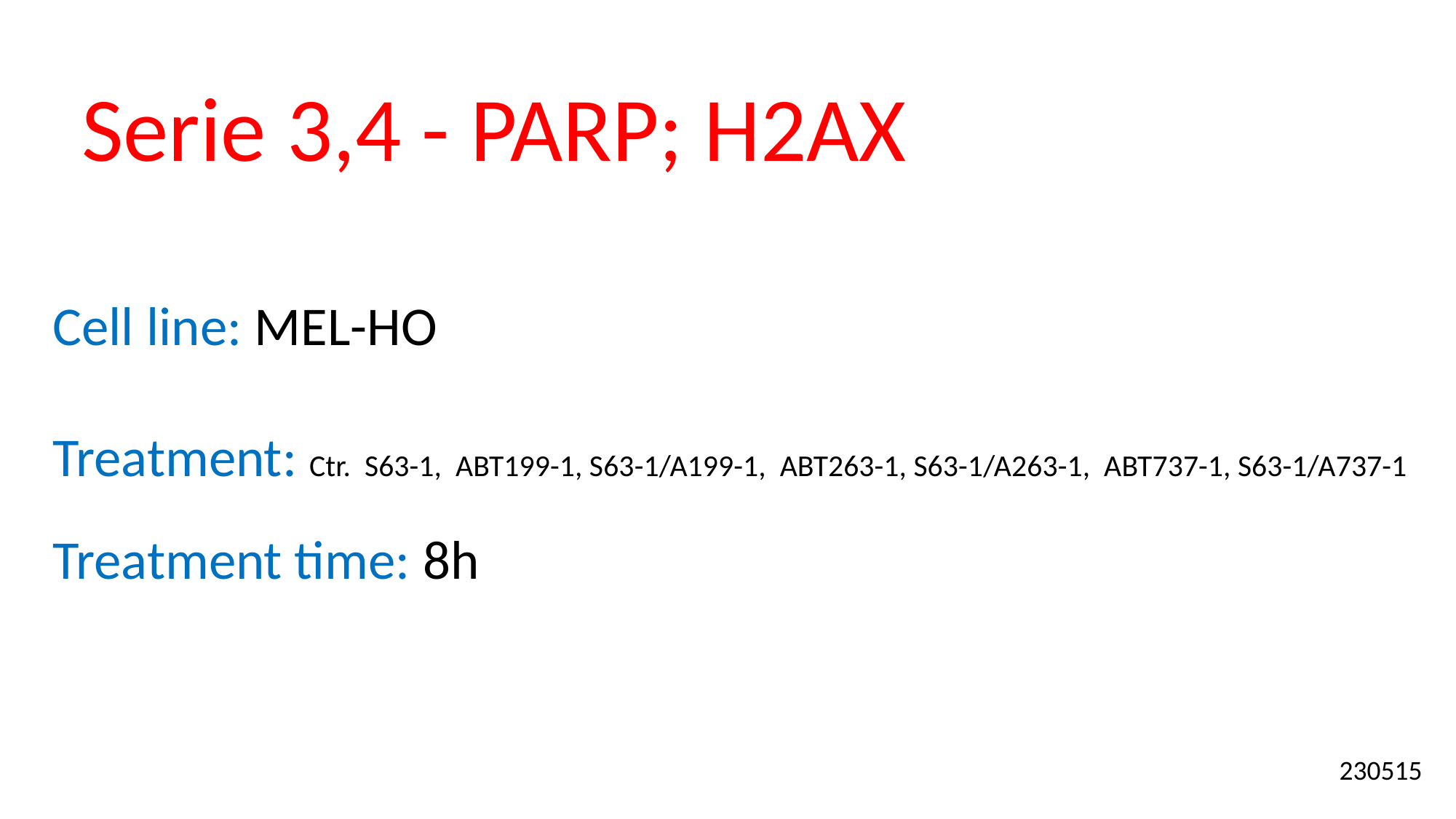

Serie 3,4 - PARP; H2AX
Cell line: MEL-HO
Treatment: Ctr. S63-1, ABT199-1, S63-1/A199-1, ABT263-1, S63-1/A263-1, ABT737-1, S63-1/A737-1
Treatment time: 8h
230515

## Slide 14
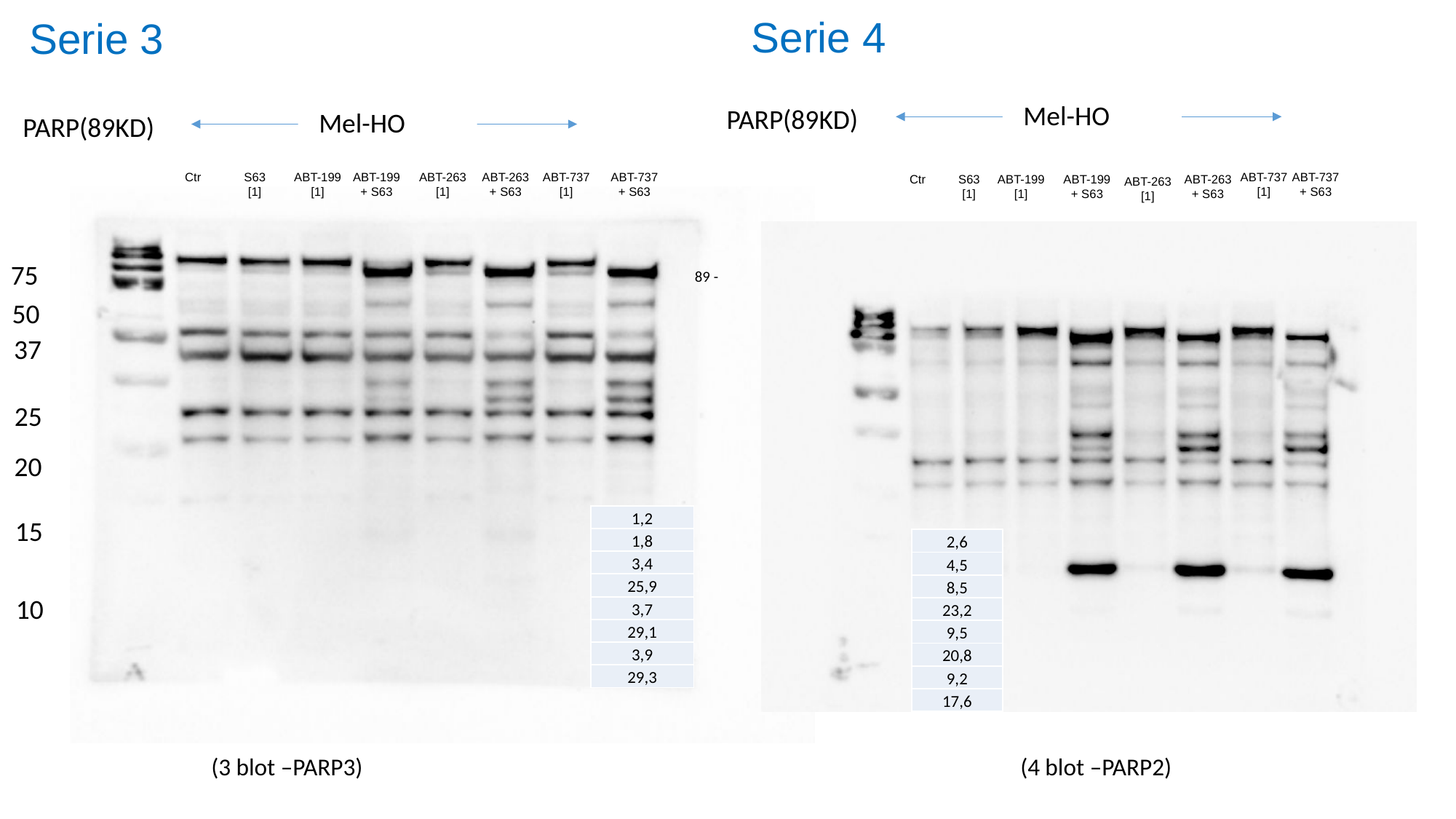

Serie 4
Serie 3
Mel-HO
PARP(89KD)
Mel-HO
PARP(89KD)
ABT-737
[1]
ABT-737
+ S63
Ctr
S63
[1]
ABT-199
[1]
ABT-199
+ S63
ABT-263
+ S63
ABT-263
[1]
Ctr
S63
[1]
ABT-199
[1]
ABT-199
+ S63
ABT-263
[1]
ABT-263
+ S63
ABT-737
[1]
ABT-737
+ S63
75
89 -
50
37
25
20
| 1,2 |
| --- |
| 1,8 |
| 3,4 |
| 25,9 |
| 3,7 |
| 29,1 |
| 3,9 |
| 29,3 |
15
| 2,6 |
| --- |
| 4,5 |
| 8,5 |
| 23,2 |
| 9,5 |
| 20,8 |
| 9,2 |
| 17,6 |
10
 (3 blot –PARP3)
 (4 blot –PARP2)

## Slide 15
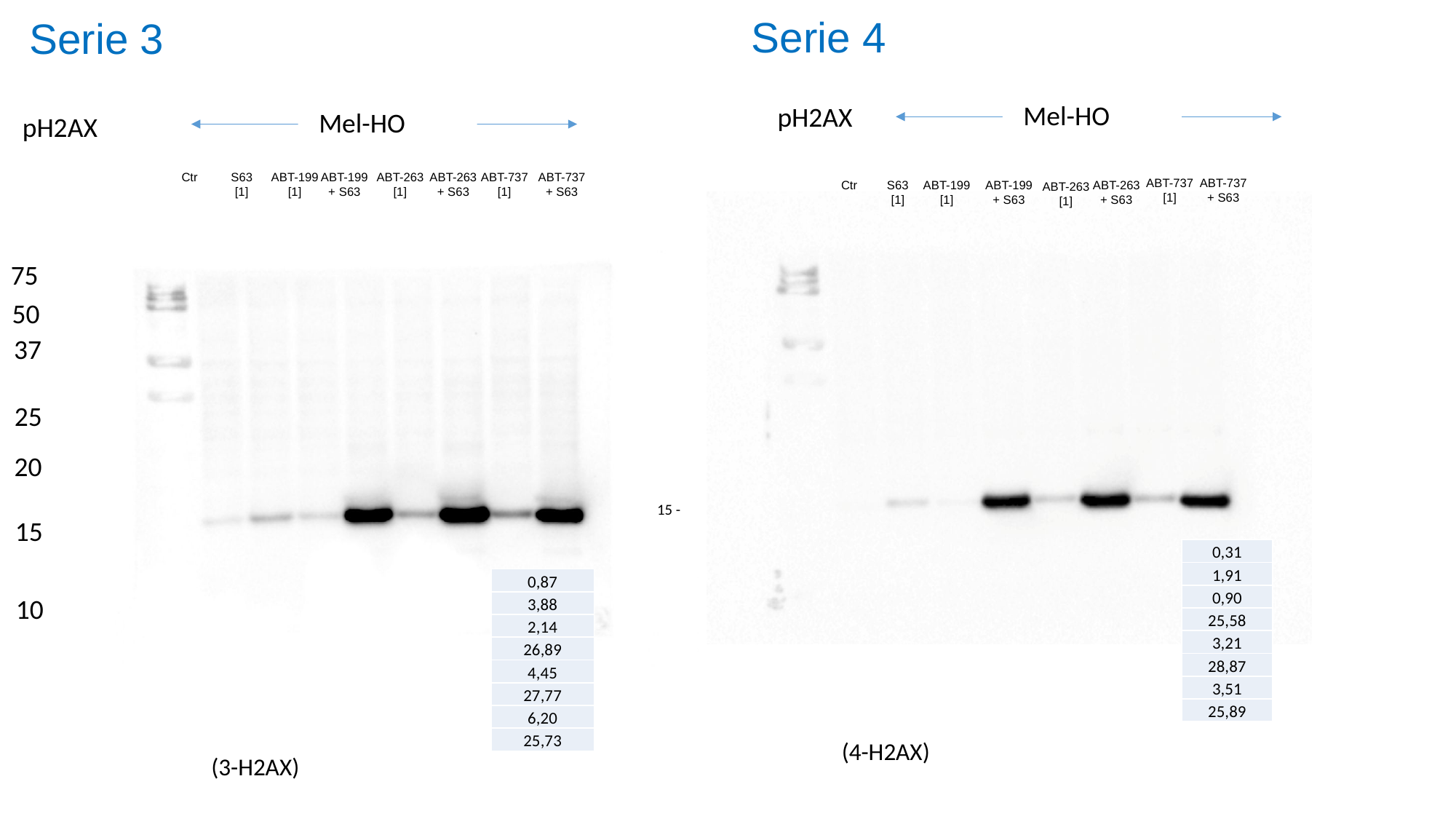

Serie 4
Serie 3
Mel-HO
pH2AX
Mel-HO
pH2AX
Ctr
S63
[1]
ABT-199
[1]
ABT-199
+ S63
ABT-263
[1]
ABT-263
+ S63
ABT-737
[1]
ABT-737
+ S63
ABT-737
[1]
ABT-737
+ S63
Ctr
S63
[1]
ABT-199
[1]
ABT-199
+ S63
ABT-263
+ S63
ABT-263
[1]
75
50
37
25
20
15 -
15
| 0,31 |
| --- |
| 1,91 |
| 0,90 |
| 25,58 |
| 3,21 |
| 28,87 |
| 3,51 |
| 25,89 |
| 0,87 |
| --- |
| 3,88 |
| 2,14 |
| 26,89 |
| 4,45 |
| 27,77 |
| 6,20 |
| 25,73 |
10
 (4-H2AX)
 (3-H2AX)

## Slide 16
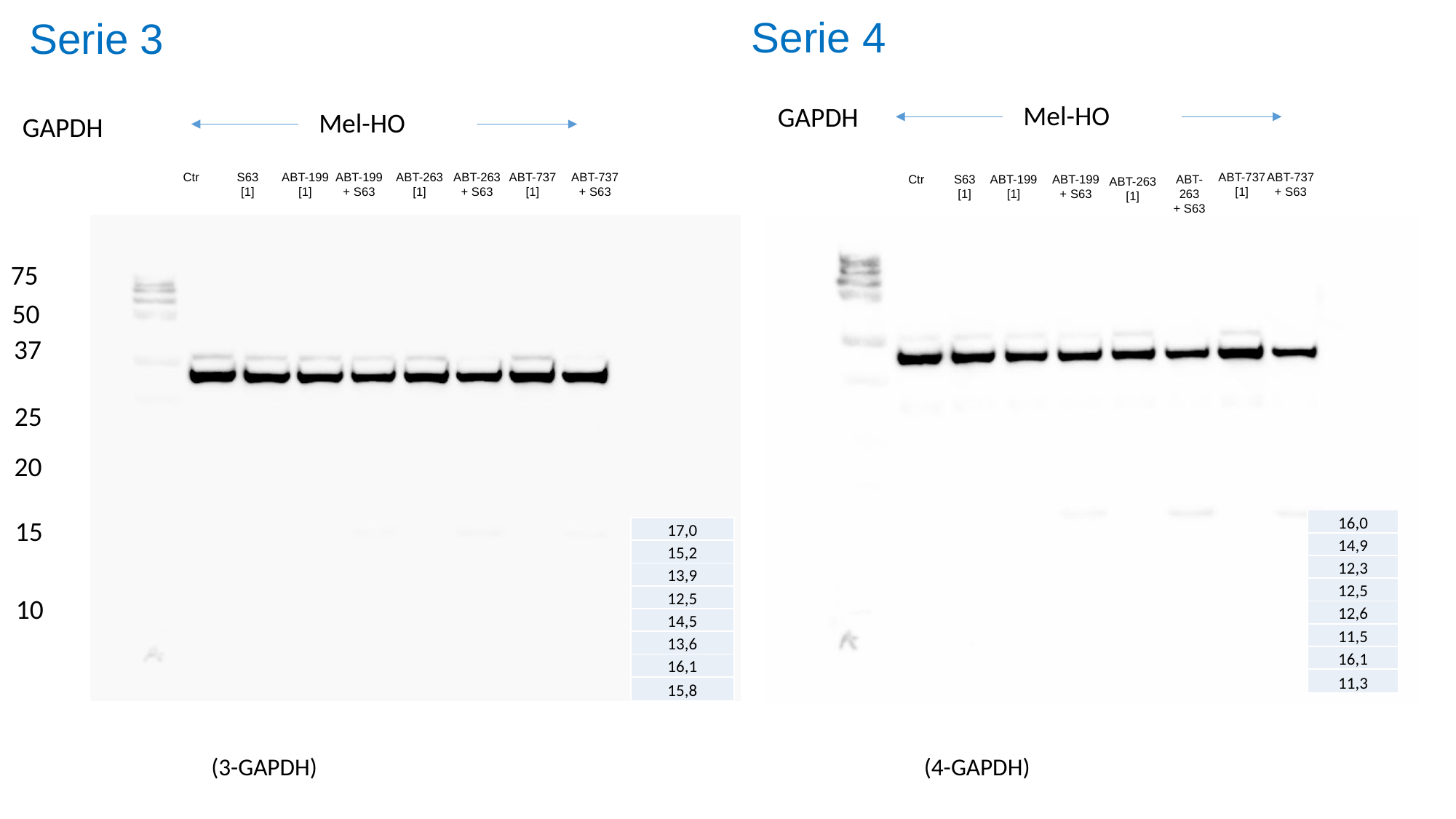

Serie 4
Serie 3
Mel-HO
GAPDH
Mel-HO
GAPDH
Ctr
S63
[1]
ABT-199
[1]
ABT-199
+ S63
ABT-263
[1]
ABT-263
+ S63
ABT-737
[1]
ABT-737
+ S63
ABT-737
[1]
ABT-737
+ S63
Ctr
S63
[1]
ABT-199
[1]
ABT-199
+ S63
ABT-263
+ S63
ABT-263
[1]
75
50
37
25
20
15
| 16,0 |
| --- |
| 14,9 |
| 12,3 |
| 12,5 |
| 12,6 |
| 11,5 |
| 16,1 |
| 11,3 |
| 17,0 |
| --- |
| 15,2 |
| 13,9 |
| 12,5 |
| 14,5 |
| 13,6 |
| 16,1 |
| 15,8 |
10
 (3-GAPDH)
 (4-GAPDH)

## Slide 17
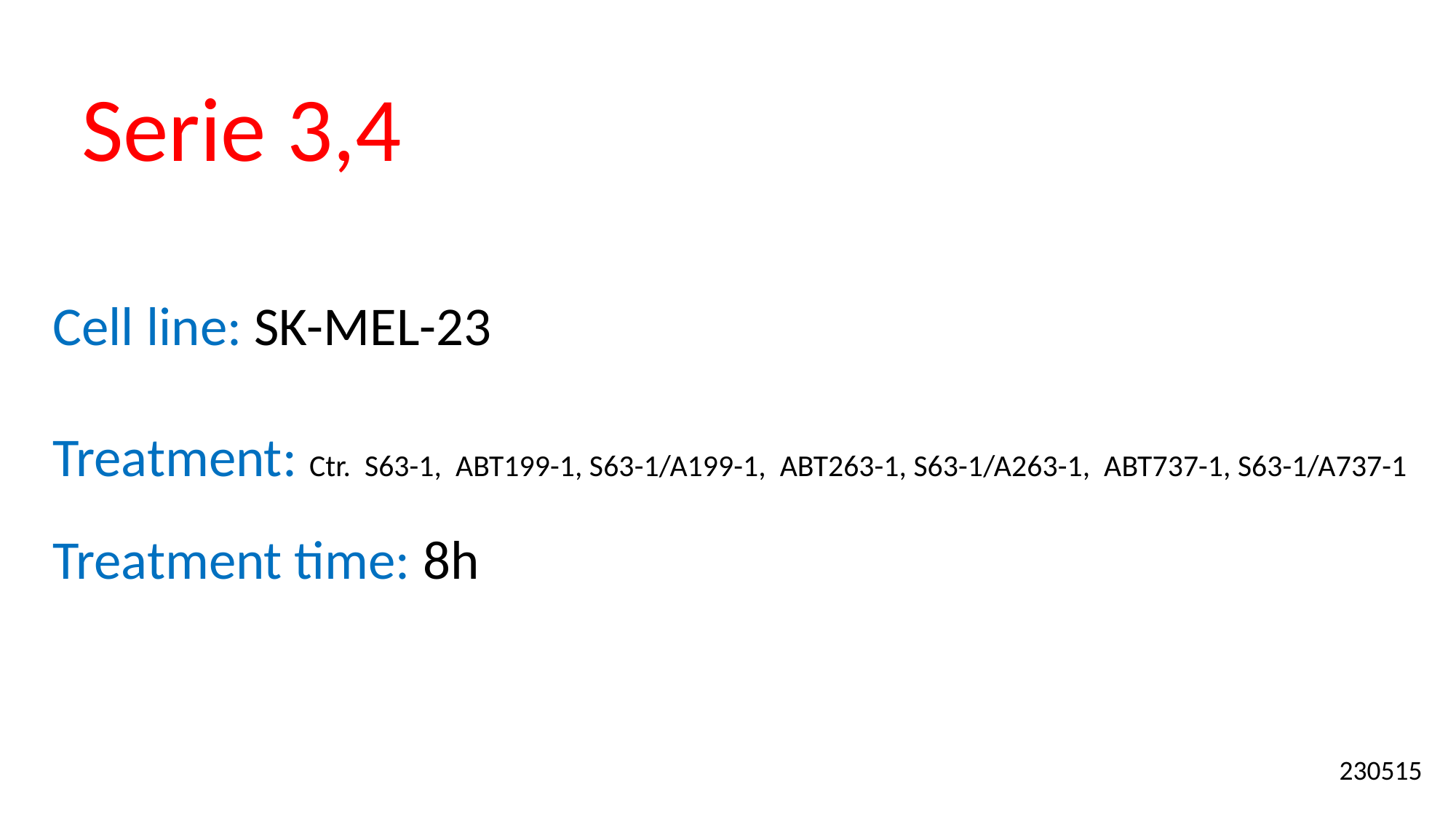

Serie 3,4
Cell line: SK-MEL-23
Treatment: Ctr. S63-1, ABT199-1, S63-1/A199-1, ABT263-1, S63-1/A263-1, ABT737-1, S63-1/A737-1
Treatment time: 8h
230515

## Slide 18
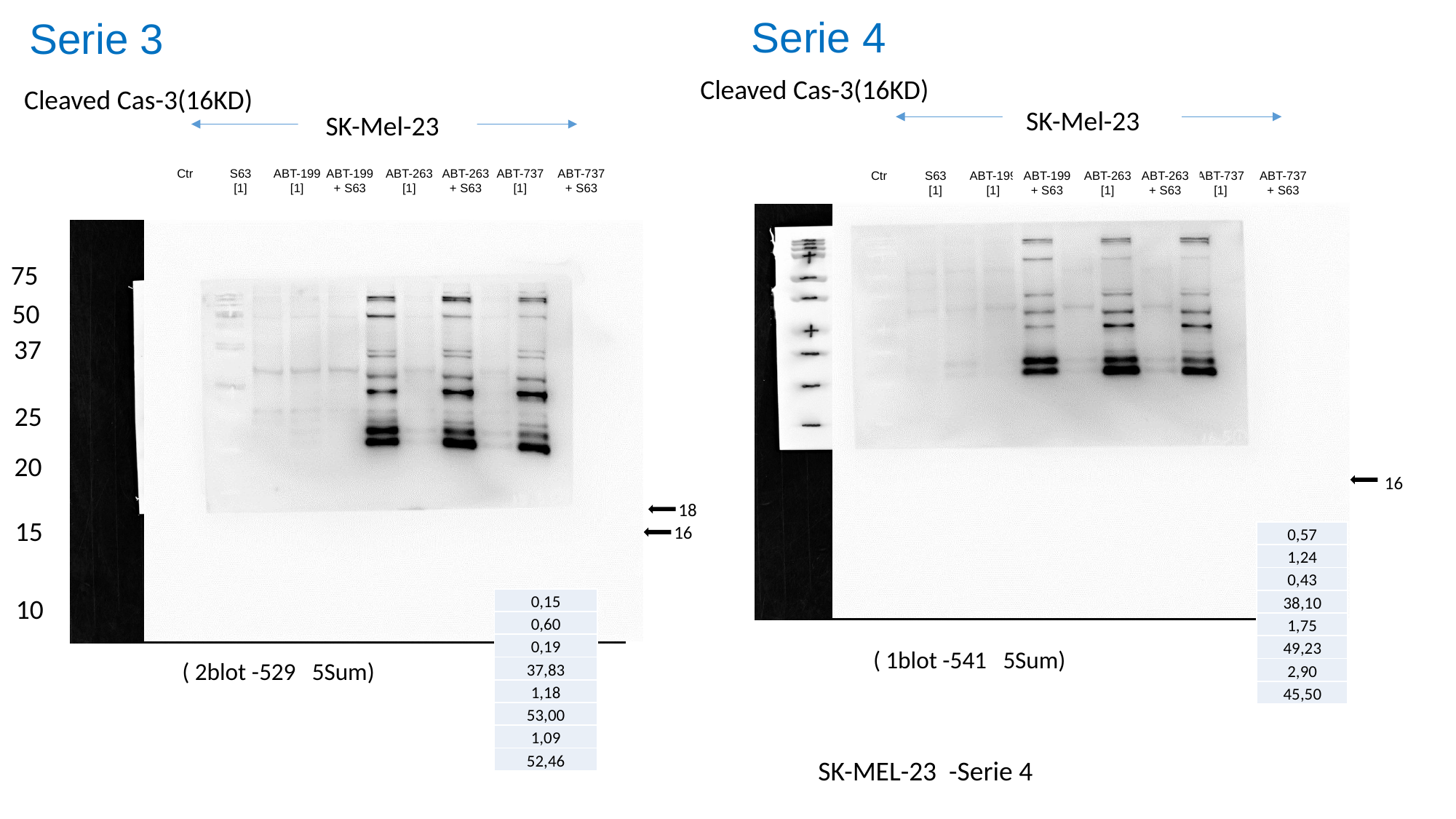

Serie 4
Serie 3
Cleaved Cas-3(16KD)
Cleaved Cas-3(16KD)
SK-Mel-23
SK-Mel-23
Ctr
S63
[1]
ABT-199
[1]
ABT-199
+ S63
ABT-263
[1]
ABT-263
+ S63
ABT-737
[1]
ABT-737
+ S63
Ctr
S63
[1]
ABT-199
[1]
ABT-199
+ S63
ABT-263
[1]
ABT-263
+ S63
ABT-737
[1]
ABT-737
+ S63
75
50
37
25
20
16
18
15
16
| 0,57 |
| --- |
| 1,24 |
| 0,43 |
| 38,10 |
| 1,75 |
| 49,23 |
| 2,90 |
| 45,50 |
10
| 0,15 |
| --- |
| 0,60 |
| 0,19 |
| 37,83 |
| 1,18 |
| 53,00 |
| 1,09 |
| 52,46 |
 ( 1blot -541 5Sum)
 ( 2blot -529 5Sum)
SK-MEL-23 -Serie 4

## Slide 19
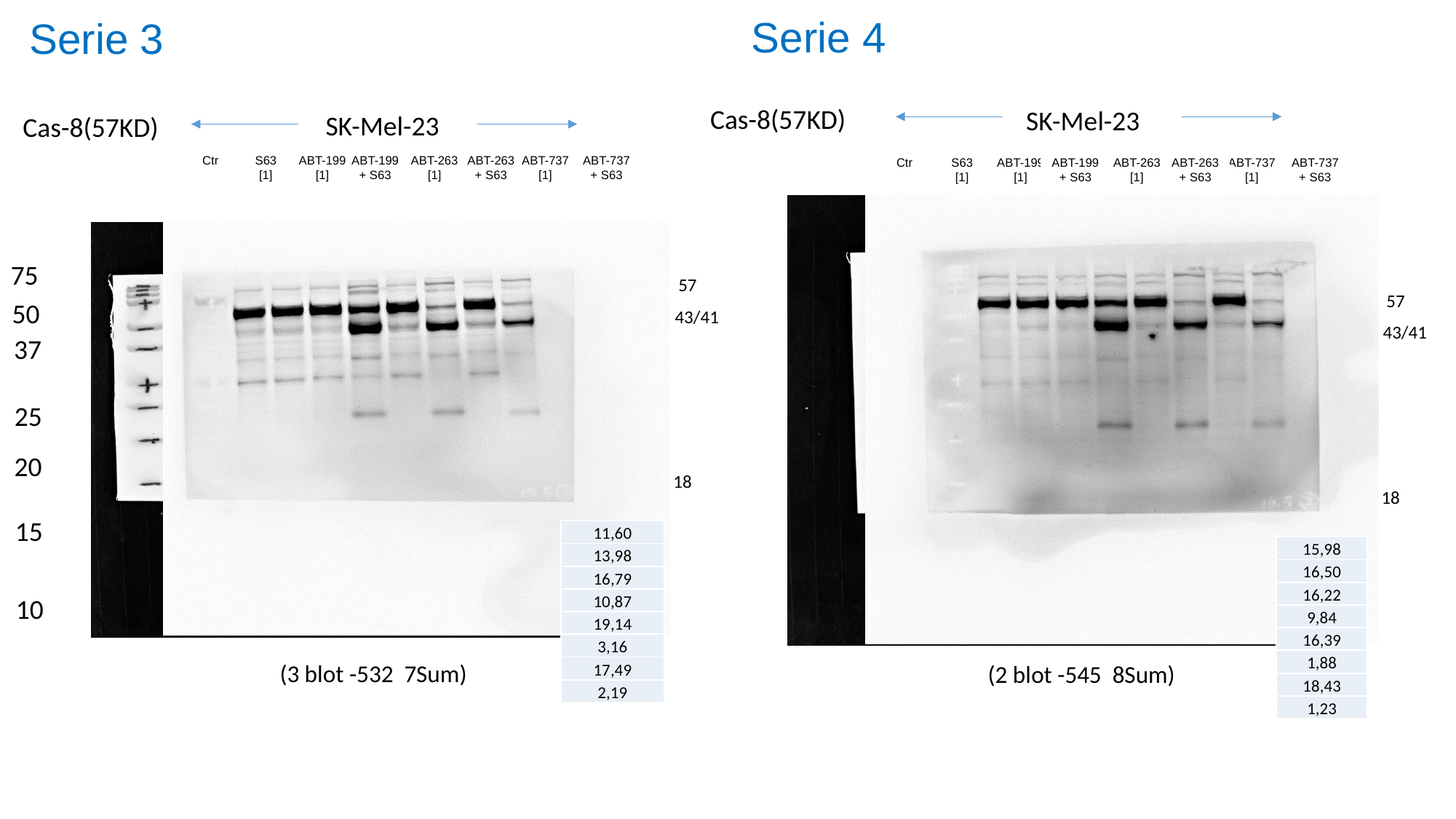

Serie 4
Serie 3
Cas-8(57KD)
SK-Mel-23
SK-Mel-23
Cas-8(57KD)
Ctr
S63
[1]
ABT-199
[1]
ABT-199
+ S63
ABT-263
[1]
ABT-263
+ S63
ABT-737
[1]
ABT-737
+ S63
Ctr
S63
[1]
ABT-199
[1]
ABT-199
+ S63
ABT-263
[1]
ABT-263
+ S63
ABT-737
[1]
ABT-737
+ S63
75
57
57
50
43/41
43/41
37
25
20
18
18
15
| 11,60 |
| --- |
| 13,98 |
| 16,79 |
| 10,87 |
| 19,14 |
| 3,16 |
| 17,49 |
| 2,19 |
| 15,98 |
| --- |
| 16,50 |
| 16,22 |
| 9,84 |
| 16,39 |
| 1,88 |
| 18,43 |
| 1,23 |
10
 (3 blot -532 7Sum)
 (2 blot -545 8Sum)

## Slide 20
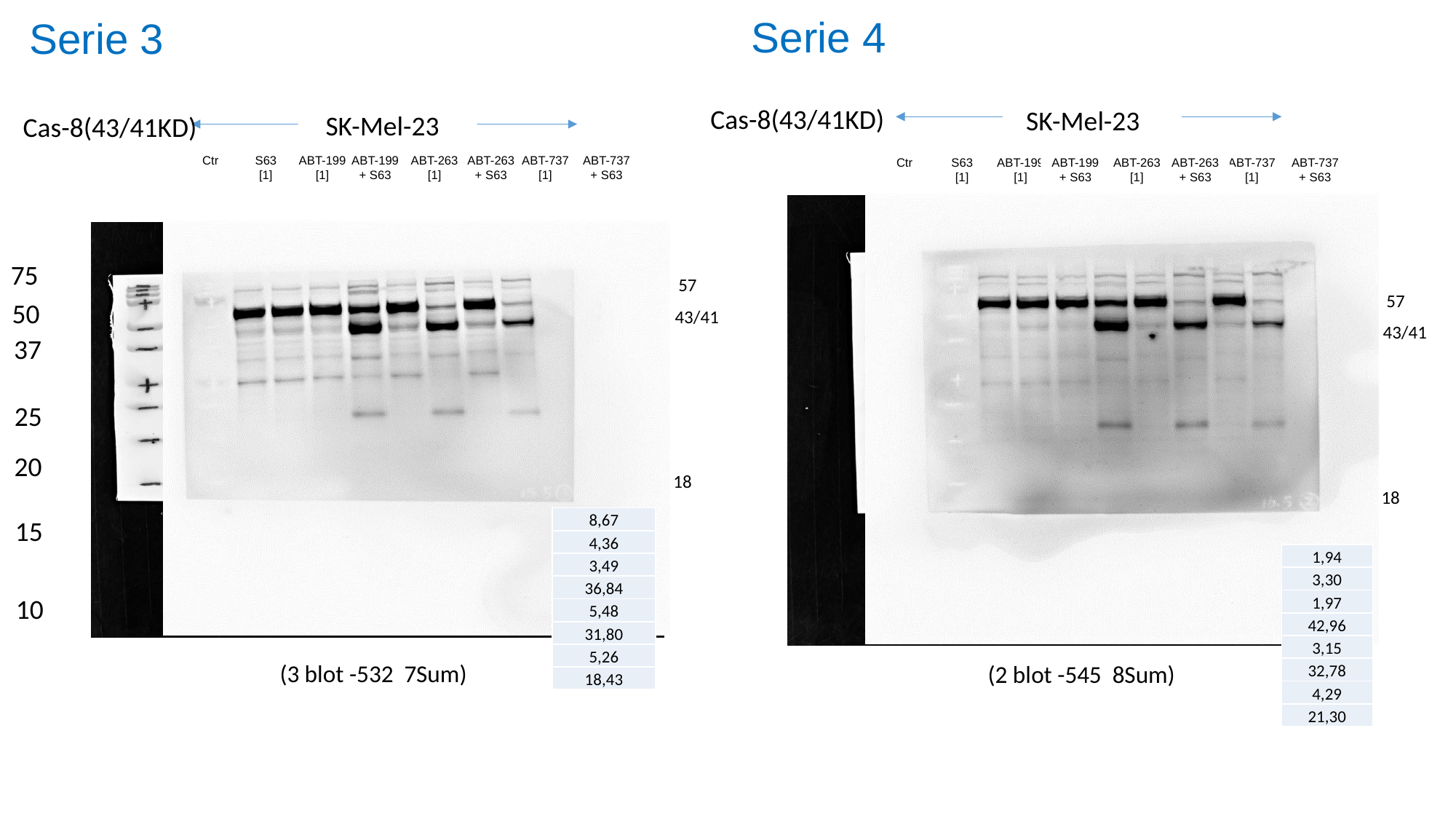

Serie 4
Serie 3
Cas-8(43/41KD)
SK-Mel-23
SK-Mel-23
Cas-8(43/41KD)
Ctr
S63
[1]
ABT-199
[1]
ABT-199
+ S63
ABT-263
[1]
ABT-263
+ S63
ABT-737
[1]
ABT-737
+ S63
Ctr
S63
[1]
ABT-199
[1]
ABT-199
+ S63
ABT-263
[1]
ABT-263
+ S63
ABT-737
[1]
ABT-737
+ S63
75
57
57
50
43/41
43/41
37
25
20
18
18
| 8,67 |
| --- |
| 4,36 |
| 3,49 |
| 36,84 |
| 5,48 |
| 31,80 |
| 5,26 |
| 18,43 |
15
| 1,94 |
| --- |
| 3,30 |
| 1,97 |
| 42,96 |
| 3,15 |
| 32,78 |
| 4,29 |
| 21,30 |
10
 (3 blot -532 7Sum)
 (2 blot -545 8Sum)

## Slide 21
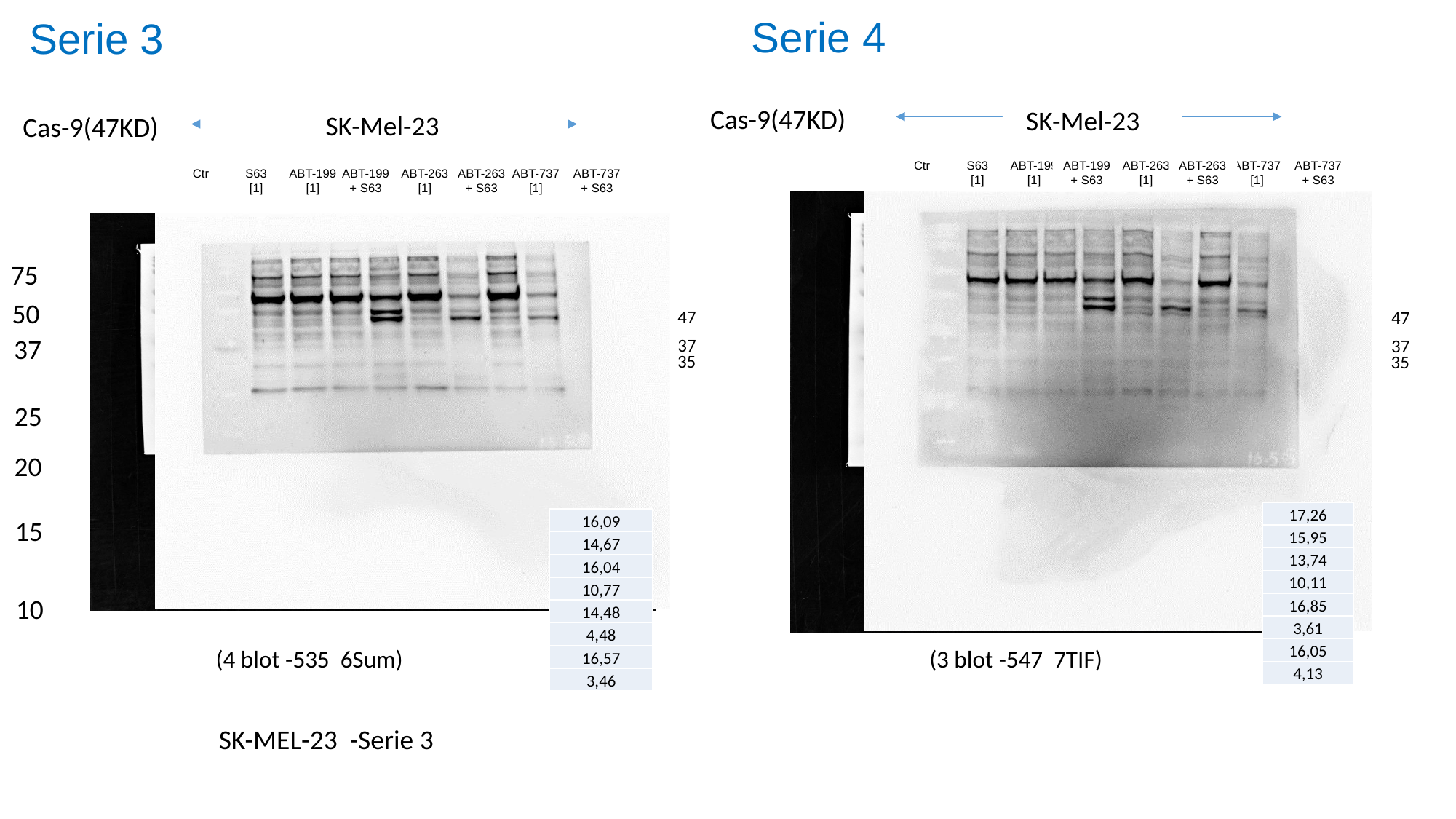

Serie 4
Serie 3
Cas-9(47KD)
SK-Mel-23
SK-Mel-23
Cas-9(47KD)
Ctr
S63
[1]
ABT-199
[1]
ABT-199
+ S63
ABT-263
[1]
ABT-263
+ S63
ABT-737
[1]
ABT-737
+ S63
Ctr
S63
[1]
ABT-199
[1]
ABT-199
+ S63
ABT-263
[1]
ABT-263
+ S63
ABT-737
[1]
ABT-737
+ S63
75
50
47
47
37
37
37
35
35
25
20
| 17,26 |
| --- |
| 15,95 |
| 13,74 |
| 10,11 |
| 16,85 |
| 3,61 |
| 16,05 |
| 4,13 |
15
| 16,09 |
| --- |
| 14,67 |
| 16,04 |
| 10,77 |
| 14,48 |
| 4,48 |
| 16,57 |
| 3,46 |
10
 (4 blot -535 6Sum)
 (3 blot -547 7TIF)
SK-MEL-23 -Serie 3

## Slide 22
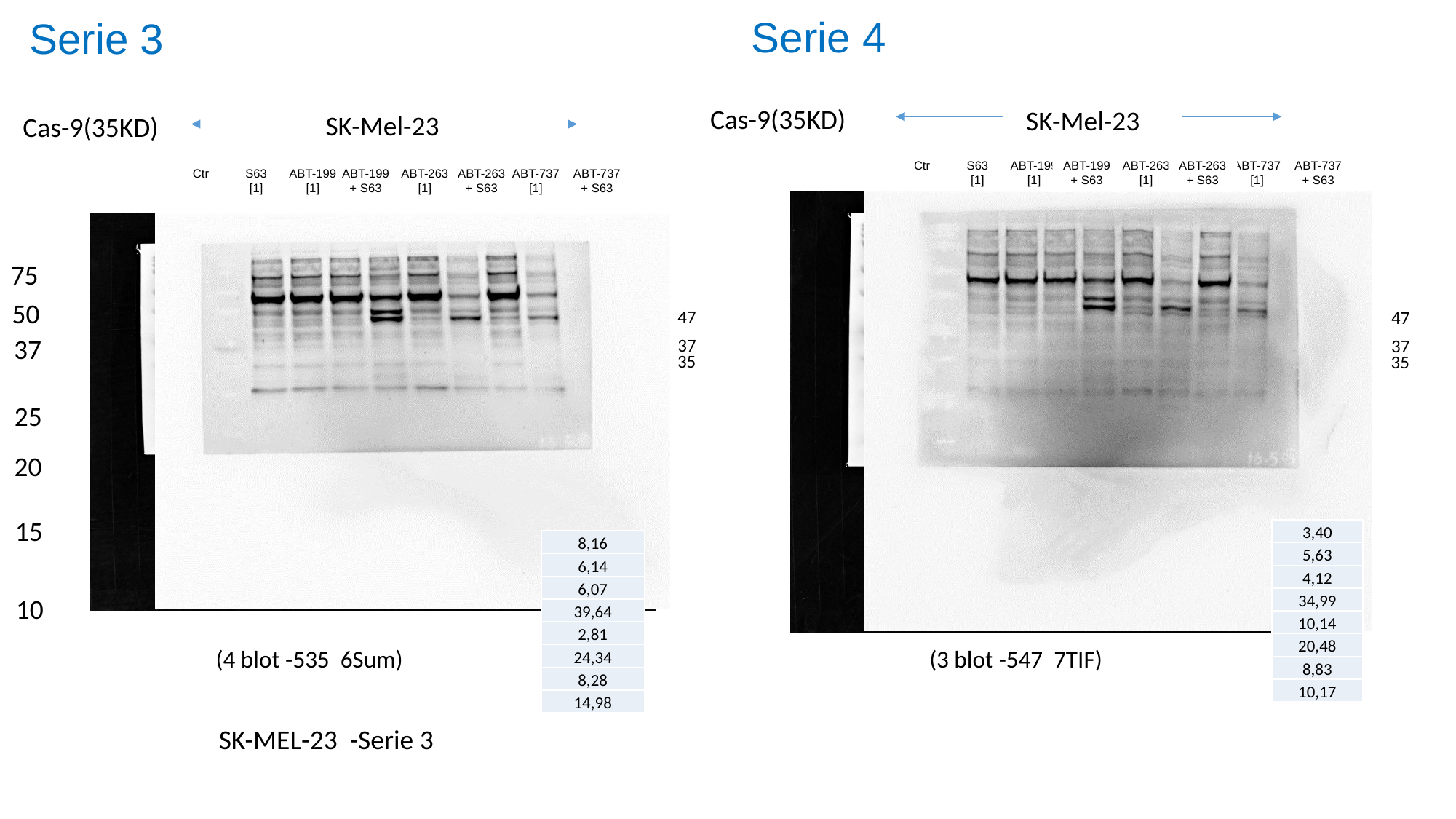

Serie 4
Serie 3
Cas-9(35KD)
SK-Mel-23
SK-Mel-23
Cas-9(35KD)
Ctr
S63
[1]
ABT-199
[1]
ABT-199
+ S63
ABT-263
[1]
ABT-263
+ S63
ABT-737
[1]
ABT-737
+ S63
Ctr
S63
[1]
ABT-199
[1]
ABT-199
+ S63
ABT-263
[1]
ABT-263
+ S63
ABT-737
[1]
ABT-737
+ S63
75
50
47
47
37
37
37
35
35
25
20
15
| 3,40 |
| --- |
| 5,63 |
| 4,12 |
| 34,99 |
| 10,14 |
| 20,48 |
| 8,83 |
| 10,17 |
| 8,16 |
| --- |
| 6,14 |
| 6,07 |
| 39,64 |
| 2,81 |
| 24,34 |
| 8,28 |
| 14,98 |
10
 (4 blot -535 6Sum)
 (3 blot -547 7TIF)
SK-MEL-23 -Serie 3

## Slide 23
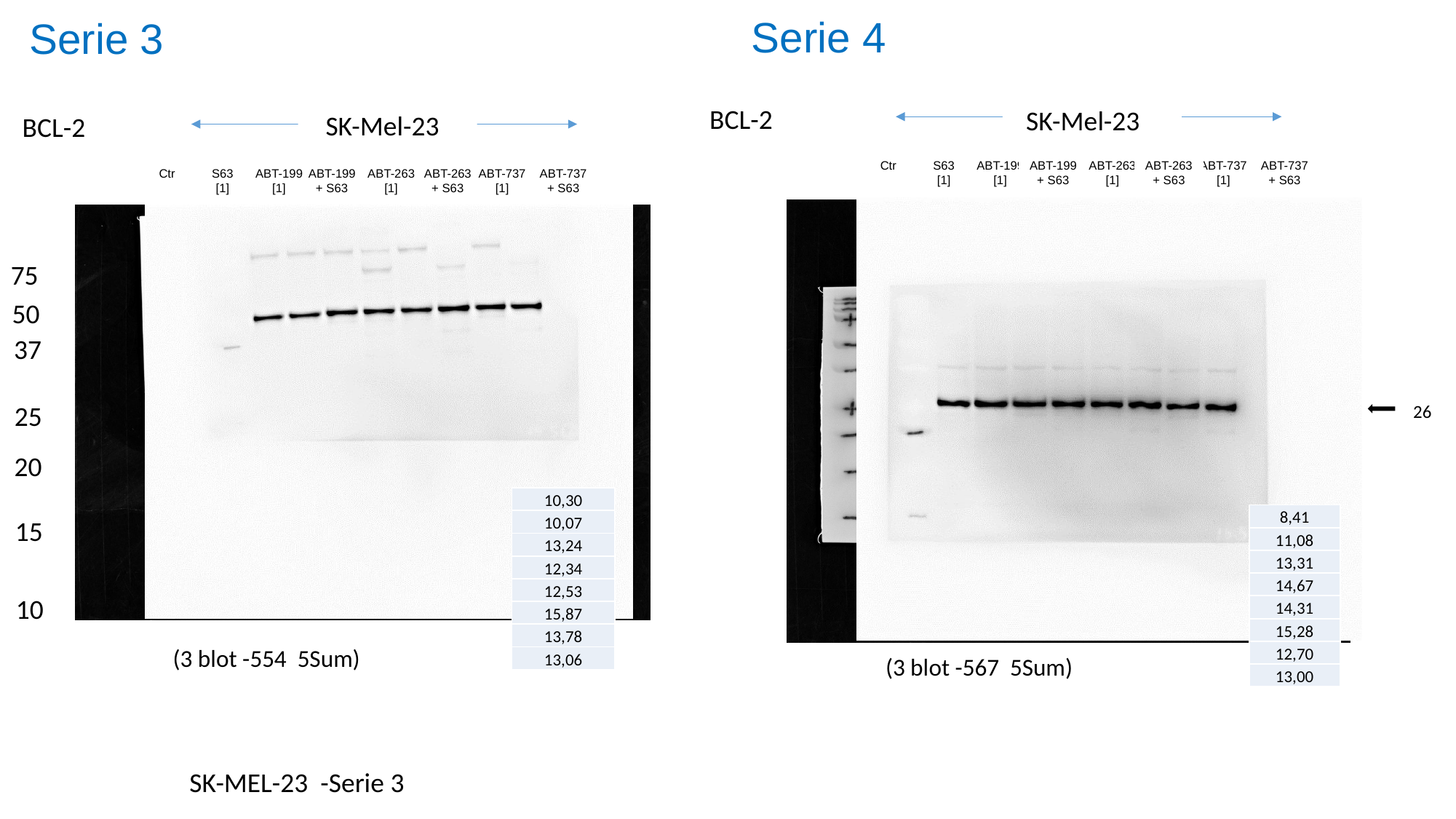

Serie 4
Serie 3
BCL-2
SK-Mel-23
SK-Mel-23
BCL-2
Ctr
S63
[1]
ABT-199
[1]
ABT-199
+ S63
ABT-263
[1]
ABT-263
+ S63
ABT-737
[1]
ABT-737
+ S63
Ctr
S63
[1]
ABT-199
[1]
ABT-199
+ S63
ABT-263
[1]
ABT-263
+ S63
ABT-737
[1]
ABT-737
+ S63
75
50
37
25
26
20
| 10,30 |
| --- |
| 10,07 |
| 13,24 |
| 12,34 |
| 12,53 |
| 15,87 |
| 13,78 |
| 13,06 |
| 8,41 |
| --- |
| 11,08 |
| 13,31 |
| 14,67 |
| 14,31 |
| 15,28 |
| 12,70 |
| 13,00 |
15
10
 (3 blot -554 5Sum)
 (3 blot -567 5Sum)
SK-MEL-23 -Serie 3

## Slide 24
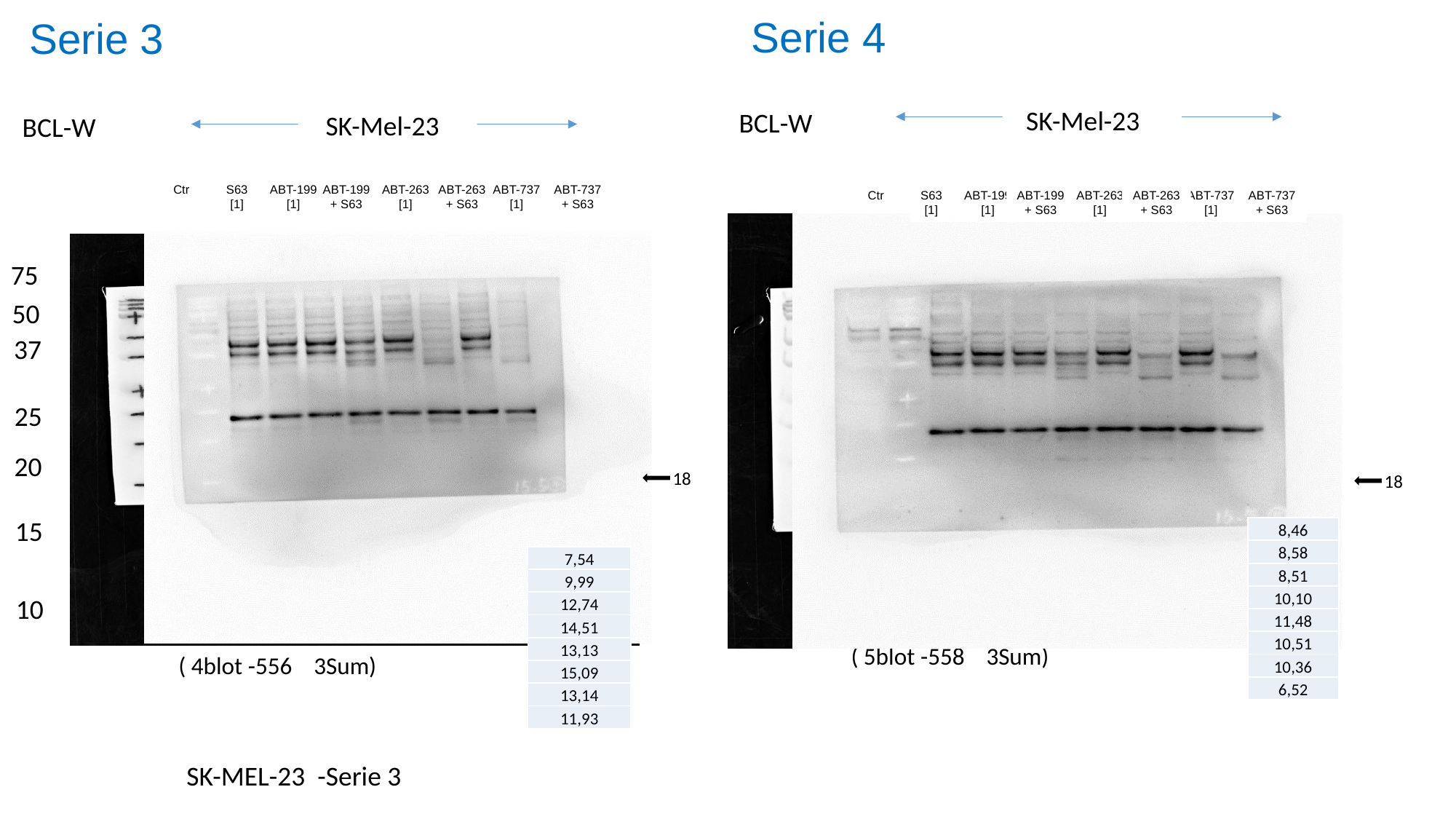

Serie 4
Serie 3
SK-Mel-23
BCL-W
SK-Mel-23
BCL-W
Ctr
S63
[1]
ABT-199
[1]
ABT-199
+ S63
ABT-263
[1]
ABT-263
+ S63
ABT-737
[1]
ABT-737
+ S63
Ctr
S63
[1]
ABT-199
[1]
ABT-199
+ S63
ABT-263
[1]
ABT-263
+ S63
ABT-737
[1]
ABT-737
+ S63
75
50
37
25
20
18
18
15
| 8,46 |
| --- |
| 8,58 |
| 8,51 |
| 10,10 |
| 11,48 |
| 10,51 |
| 10,36 |
| 6,52 |
| 7,54 |
| --- |
| 9,99 |
| 12,74 |
| 14,51 |
| 13,13 |
| 15,09 |
| 13,14 |
| 11,93 |
10
 ( 5blot -558 3Sum)
 ( 4blot -556 3Sum)
SK-MEL-23 -Serie 3

## Slide 25
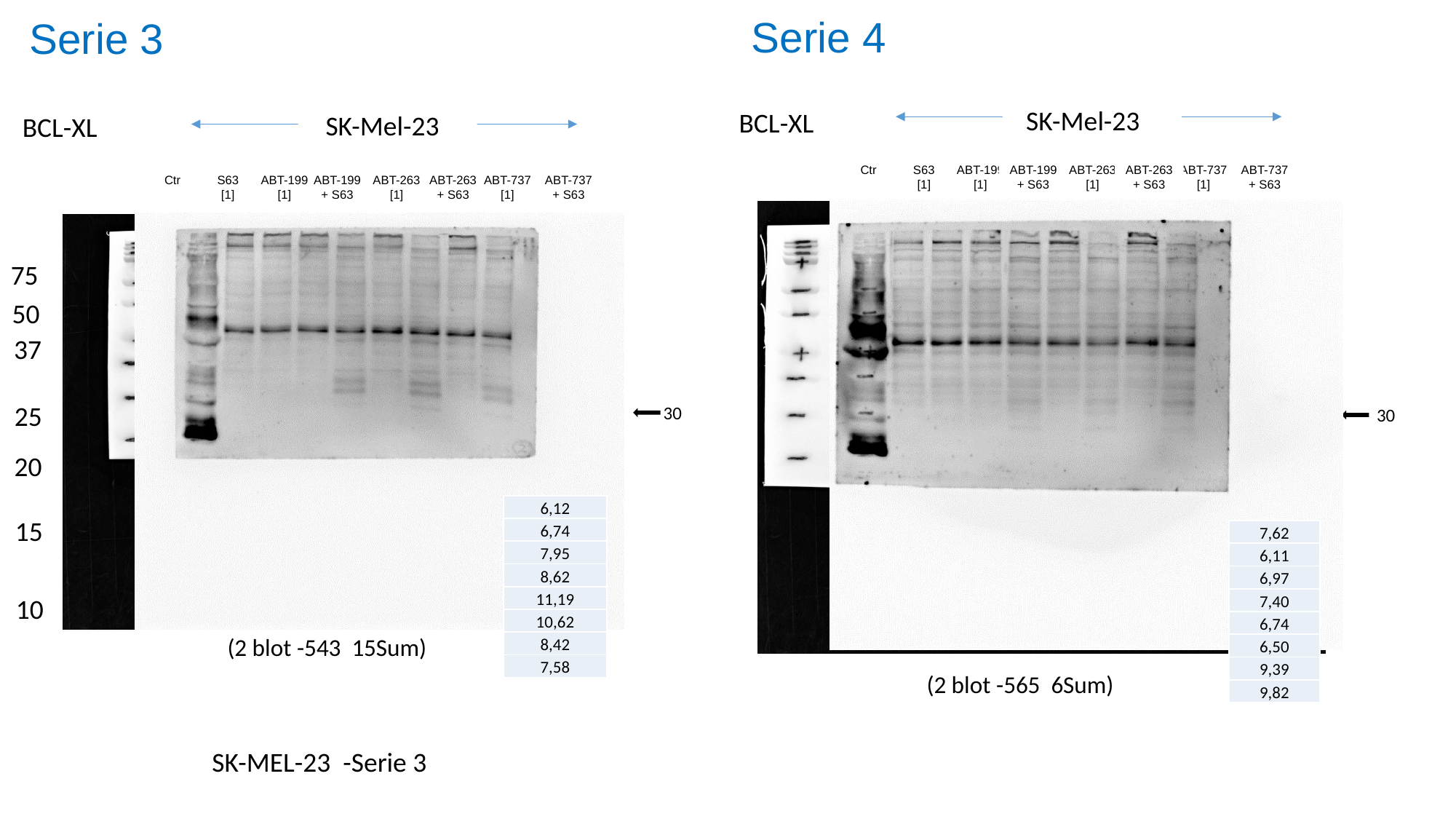

Serie 4
Serie 3
SK-Mel-23
BCL-XL
SK-Mel-23
BCL-XL
Ctr
S63
[1]
ABT-199
[1]
ABT-199
+ S63
ABT-263
[1]
ABT-263
+ S63
ABT-737
[1]
ABT-737
+ S63
Ctr
S63
[1]
ABT-199
[1]
ABT-199
+ S63
ABT-263
[1]
ABT-263
+ S63
ABT-737
[1]
ABT-737
+ S63
75
50
37
25
30
30
20
| 6,12 |
| --- |
| 6,74 |
| 7,95 |
| 8,62 |
| 11,19 |
| 10,62 |
| 8,42 |
| 7,58 |
15
| 7,62 |
| --- |
| 6,11 |
| 6,97 |
| 7,40 |
| 6,74 |
| 6,50 |
| 9,39 |
| 9,82 |
10
 (2 blot -543 15Sum)
 (2 blot -565 6Sum)
SK-MEL-23 -Serie 3

## Slide 26
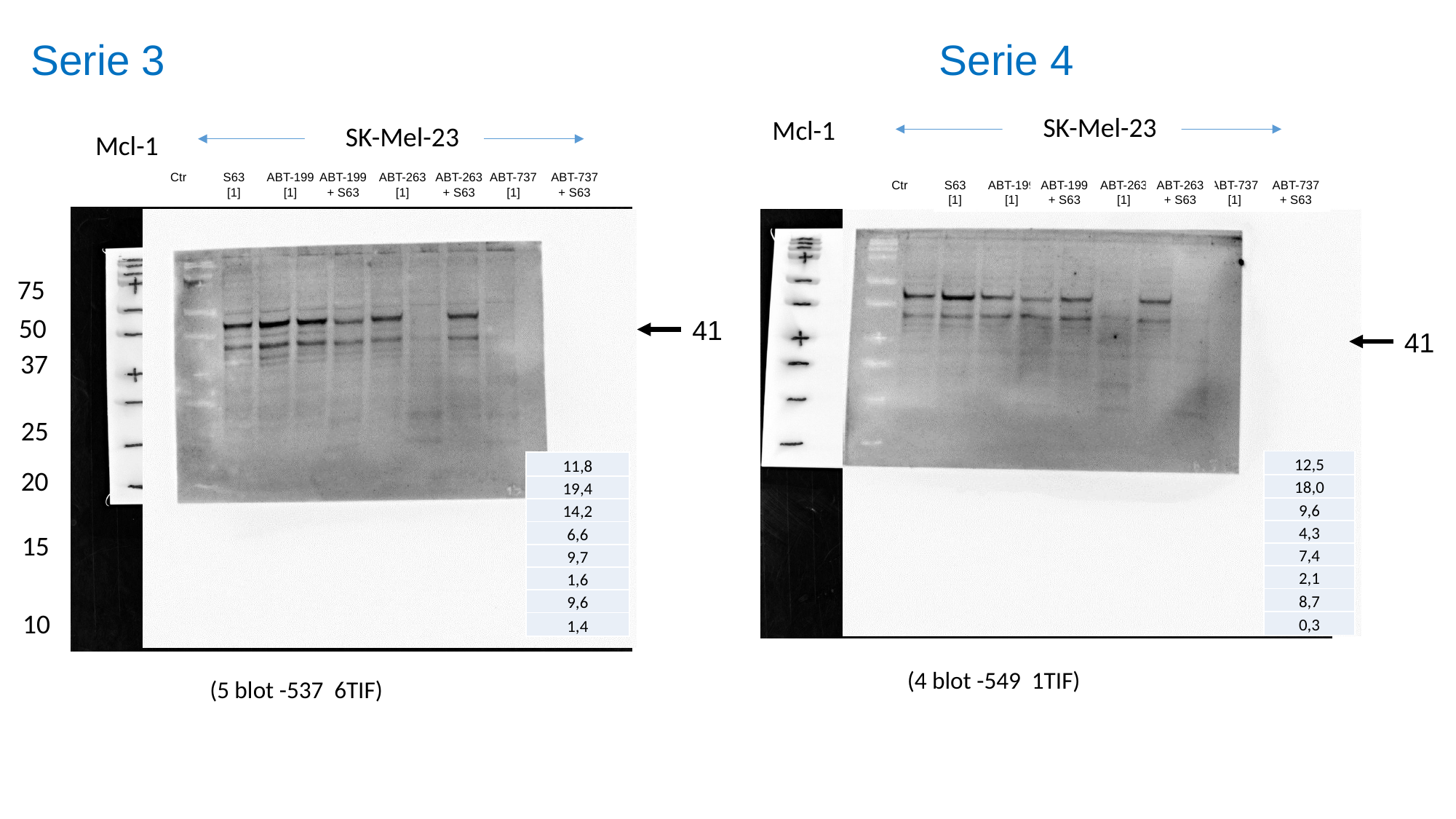

Serie 3
Serie 4
SK-Mel-23
Mcl-1
SK-Mel-23
Mcl-1
Ctr
S63
[1]
ABT-199
[1]
ABT-199
+ S63
ABT-263
[1]
ABT-263
+ S63
ABT-737
[1]
ABT-737
+ S63
Ctr
S63
[1]
ABT-199
[1]
ABT-199
+ S63
ABT-263
[1]
ABT-263
+ S63
ABT-737
[1]
ABT-737
+ S63
75
50
41
41
37
25
| 12,5 |
| --- |
| 18,0 |
| 9,6 |
| 4,3 |
| 7,4 |
| 2,1 |
| 8,7 |
| 0,3 |
| 11,8 |
| --- |
| 19,4 |
| 14,2 |
| 6,6 |
| 9,7 |
| 1,6 |
| 9,6 |
| 1,4 |
20
15
10
 (4 blot -549 1TIF)
 (5 blot -537 6TIF)

## Slide 27
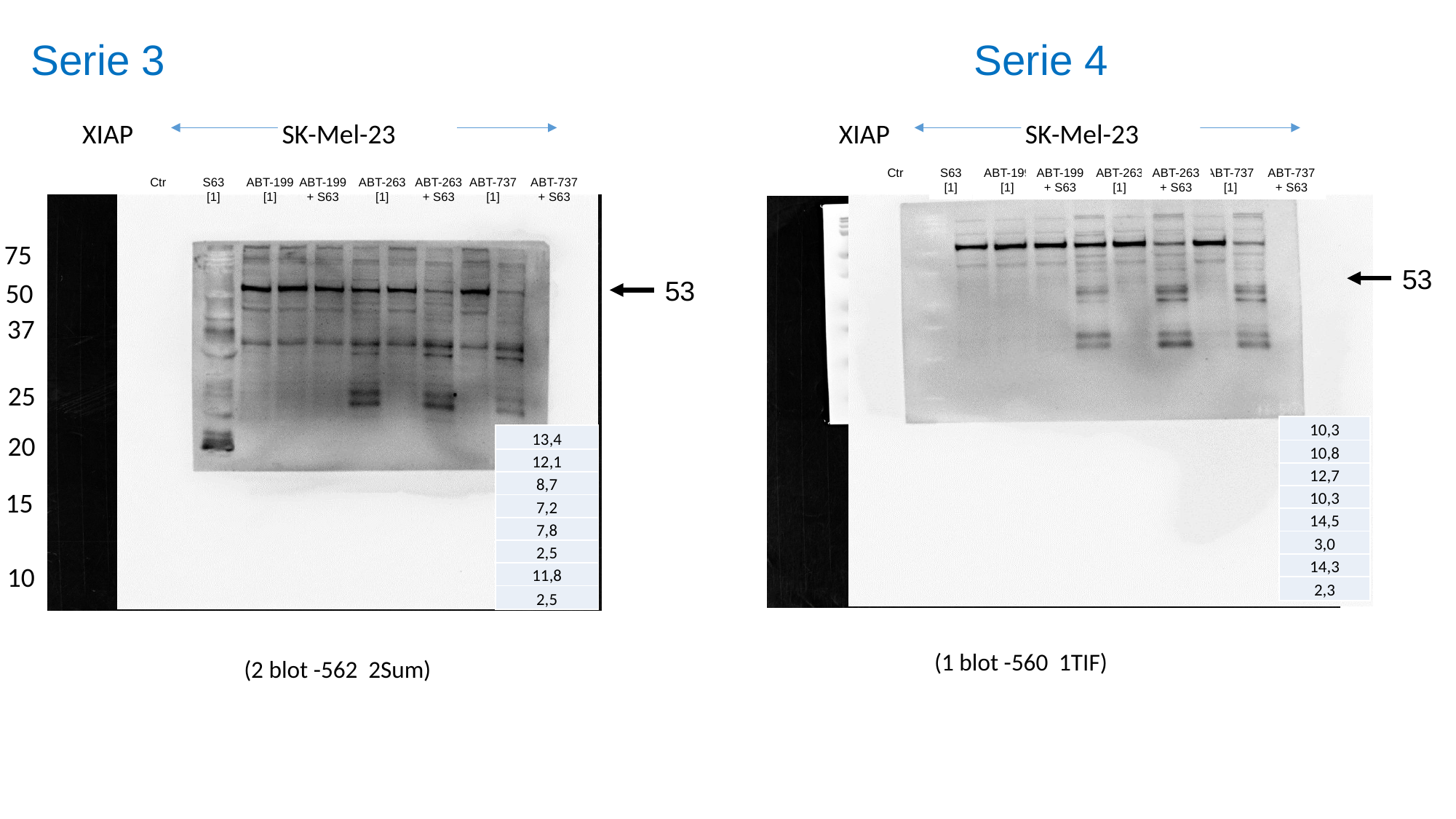

Serie 3
Serie 4
 XIAP
SK-Mel-23
 XIAP
SK-Mel-23
Ctr
S63
[1]
ABT-199
[1]
ABT-199
+ S63
ABT-263
[1]
ABT-263
+ S63
ABT-737
[1]
ABT-737
+ S63
Ctr
S63
[1]
ABT-199
[1]
ABT-199
+ S63
ABT-263
[1]
ABT-263
+ S63
ABT-737
[1]
ABT-737
+ S63
75
53
53
50
37
25
| 10,3 |
| --- |
| 10,8 |
| 12,7 |
| 10,3 |
| 14,5 |
| 3,0 |
| 14,3 |
| 2,3 |
20
| 13,4 |
| --- |
| 12,1 |
| 8,7 |
| 7,2 |
| 7,8 |
| 2,5 |
| 11,8 |
| 2,5 |
15
10
 (1 blot -560 1TIF)
 (2 blot -562 2Sum)

## Slide 28
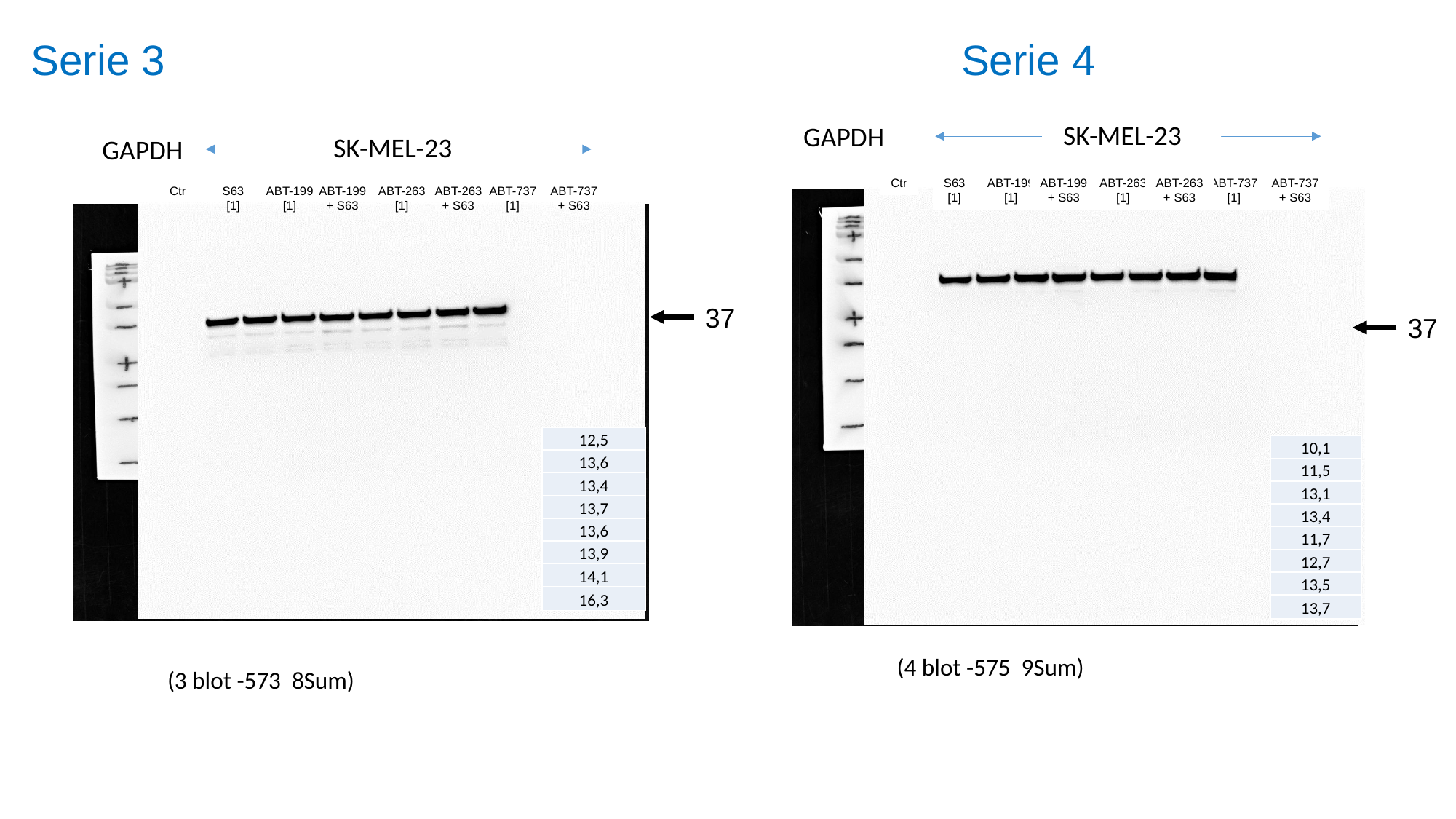

18
16
Serie 3
Serie 4
SK-MEL-23
 GAPDH
SK-MEL-23
 GAPDH
Ctr
S63
[1]
ABT-199
[1]
ABT-199
+ S63
ABT-263
[1]
ABT-263
+ S63
ABT-737
[1]
ABT-737
+ S63
Ctr
S63
[1]
ABT-199
[1]
ABT-199
+ S63
ABT-263
[1]
ABT-263
+ S63
ABT-737
[1]
ABT-737
+ S63
37
37
| 12,5 |
| --- |
| 13,6 |
| 13,4 |
| 13,7 |
| 13,6 |
| 13,9 |
| 14,1 |
| 16,3 |
| 10,1 |
| --- |
| 11,5 |
| 13,1 |
| 13,4 |
| 11,7 |
| 12,7 |
| 13,5 |
| 13,7 |
 (4 blot -575 9Sum)
 (3 blot -573 8Sum)

## Slide 29
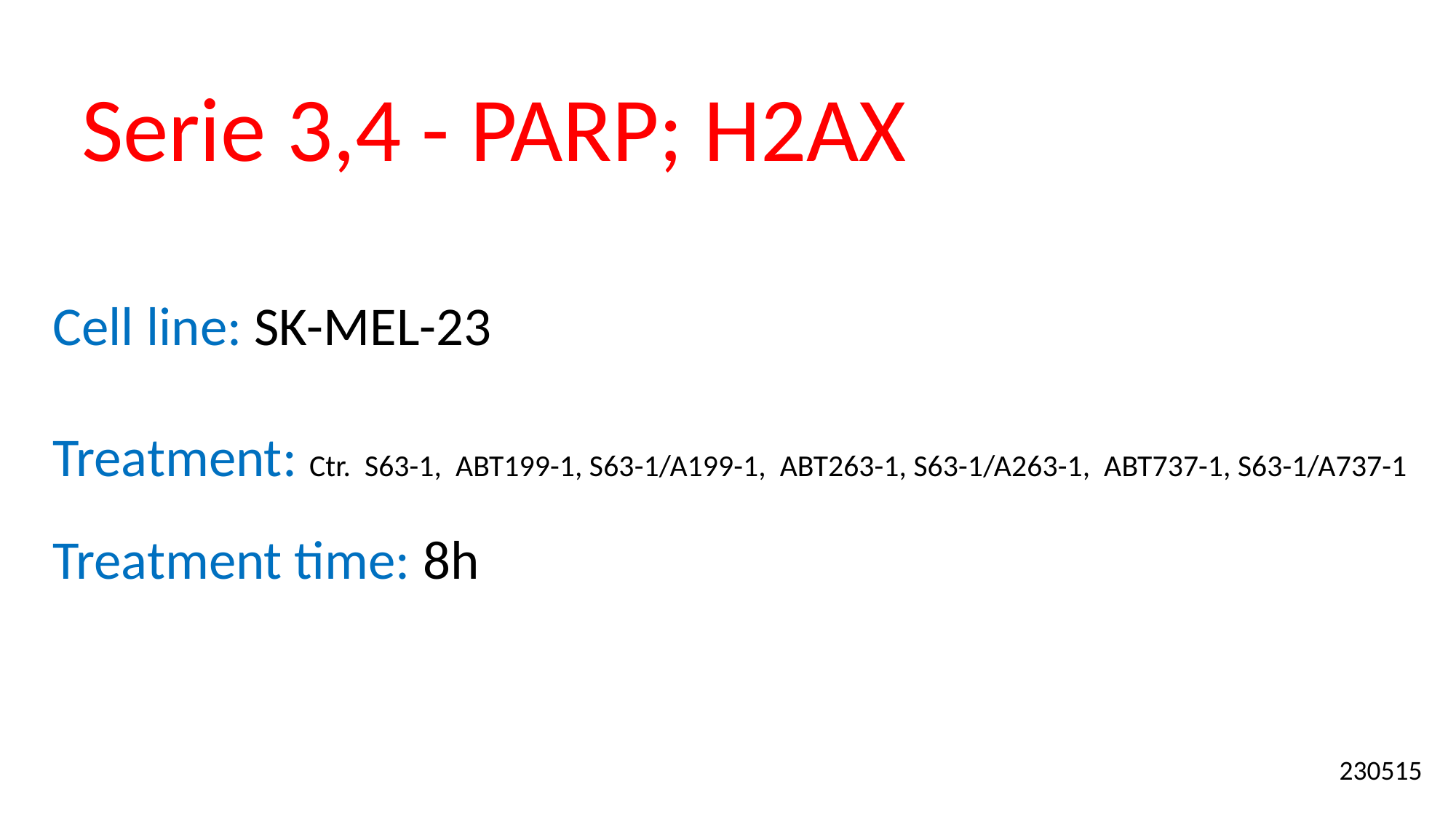

Serie 3,4 - PARP; H2AX
Cell line: SK-MEL-23
Treatment: Ctr. S63-1, ABT199-1, S63-1/A199-1, ABT263-1, S63-1/A263-1, ABT737-1, S63-1/A737-1
Treatment time: 8h
230515

## Slide 30
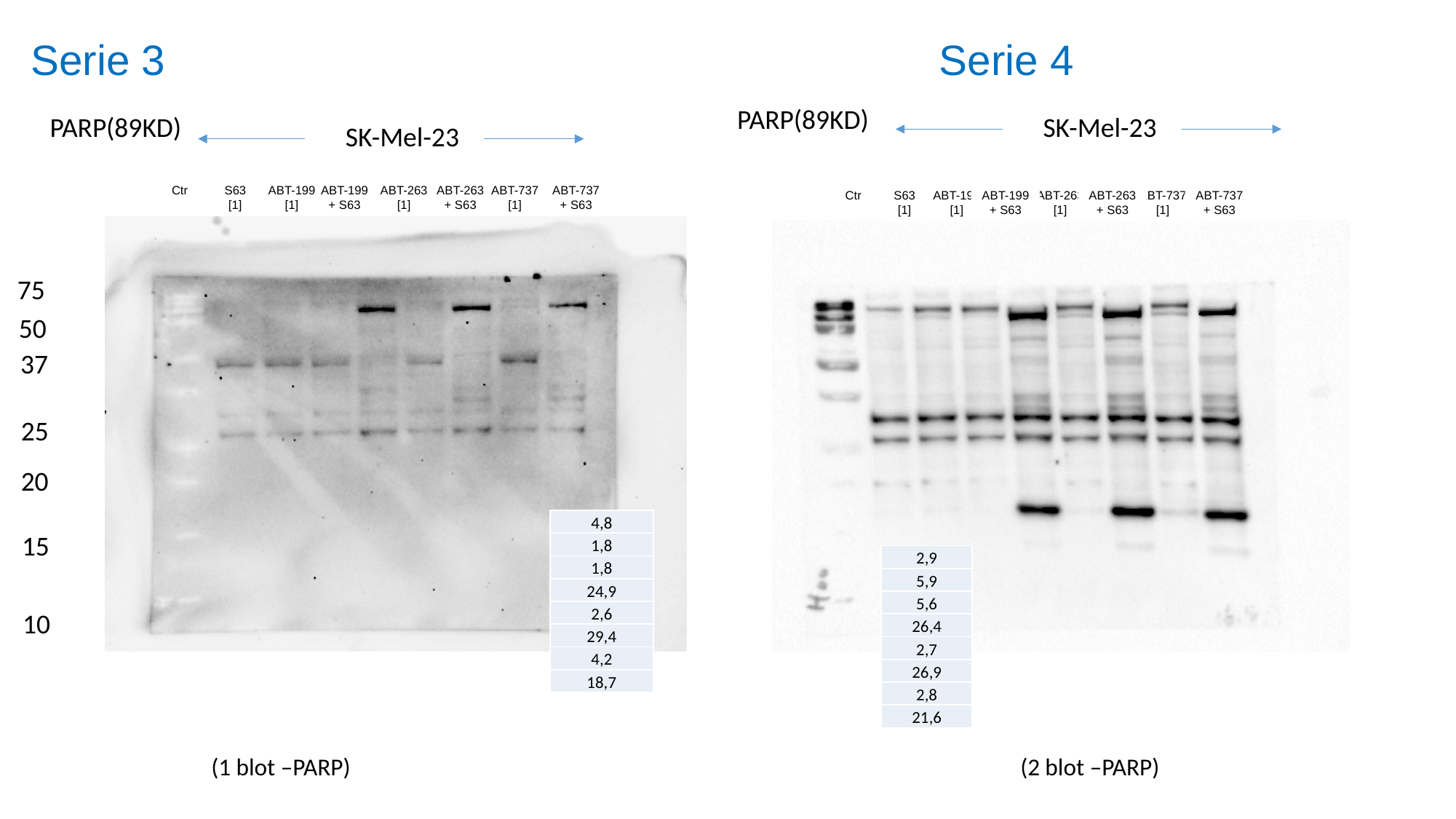

Serie 3
Serie 4
PARP(89KD)
SK-Mel-23
PARP(89KD)
SK-Mel-23
Ctr
S63
[1]
ABT-199
[1]
ABT-199
+ S63
ABT-263
[1]
ABT-263
+ S63
ABT-737
[1]
ABT-737
+ S63
Ctr
S63
[1]
ABT-199
[1]
ABT-199
+ S63
ABT-263
[1]
ABT-263
+ S63
ABT-737
[1]
ABT-737
+ S63
75
50
37
25
20
| 4,8 |
| --- |
| 1,8 |
| 1,8 |
| 24,9 |
| 2,6 |
| 29,4 |
| 4,2 |
| 18,7 |
15
| 2,9 |
| --- |
| 5,9 |
| 5,6 |
| 26,4 |
| 2,7 |
| 26,9 |
| 2,8 |
| 21,6 |
10
 (1 blot –PARP)
 (2 blot –PARP)

## Slide 31
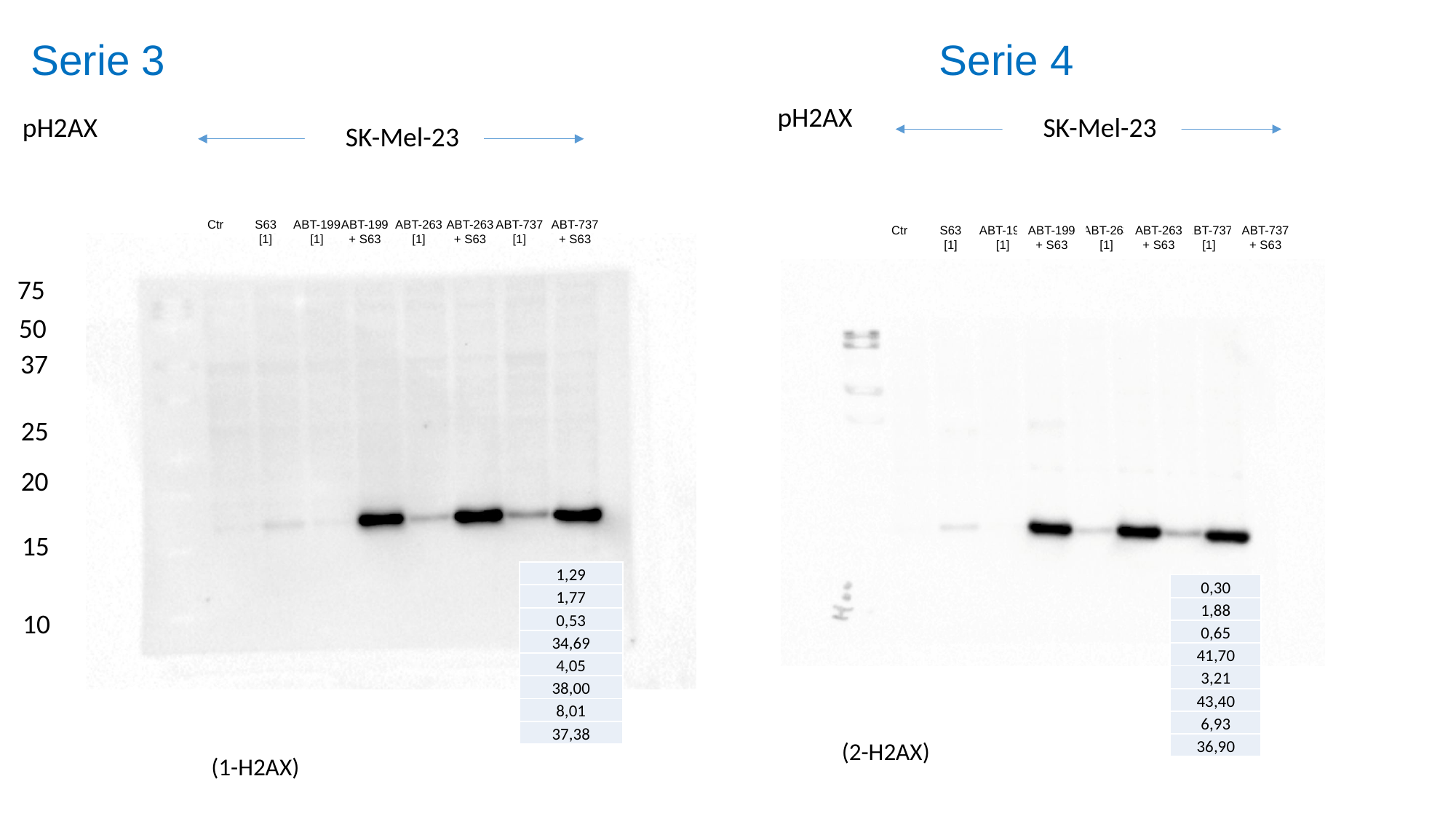

Serie 3
Serie 4
pH2AX
SK-Mel-23
pH2AX
SK-Mel-23
Ctr
S63
[1]
ABT-199
[1]
ABT-199
+ S63
ABT-263
[1]
ABT-263
+ S63
ABT-737
[1]
ABT-737
+ S63
Ctr
S63
[1]
ABT-199
[1]
ABT-199
+ S63
ABT-263
[1]
ABT-263
+ S63
ABT-737
[1]
ABT-737
+ S63
75
50
37
25
20
15
| 1,29 |
| --- |
| 1,77 |
| 0,53 |
| 34,69 |
| 4,05 |
| 38,00 |
| 8,01 |
| 37,38 |
| 0,30 |
| --- |
| 1,88 |
| 0,65 |
| 41,70 |
| 3,21 |
| 43,40 |
| 6,93 |
| 36,90 |
10
 (2-H2AX)
 (1-H2AX)

## Slide 32
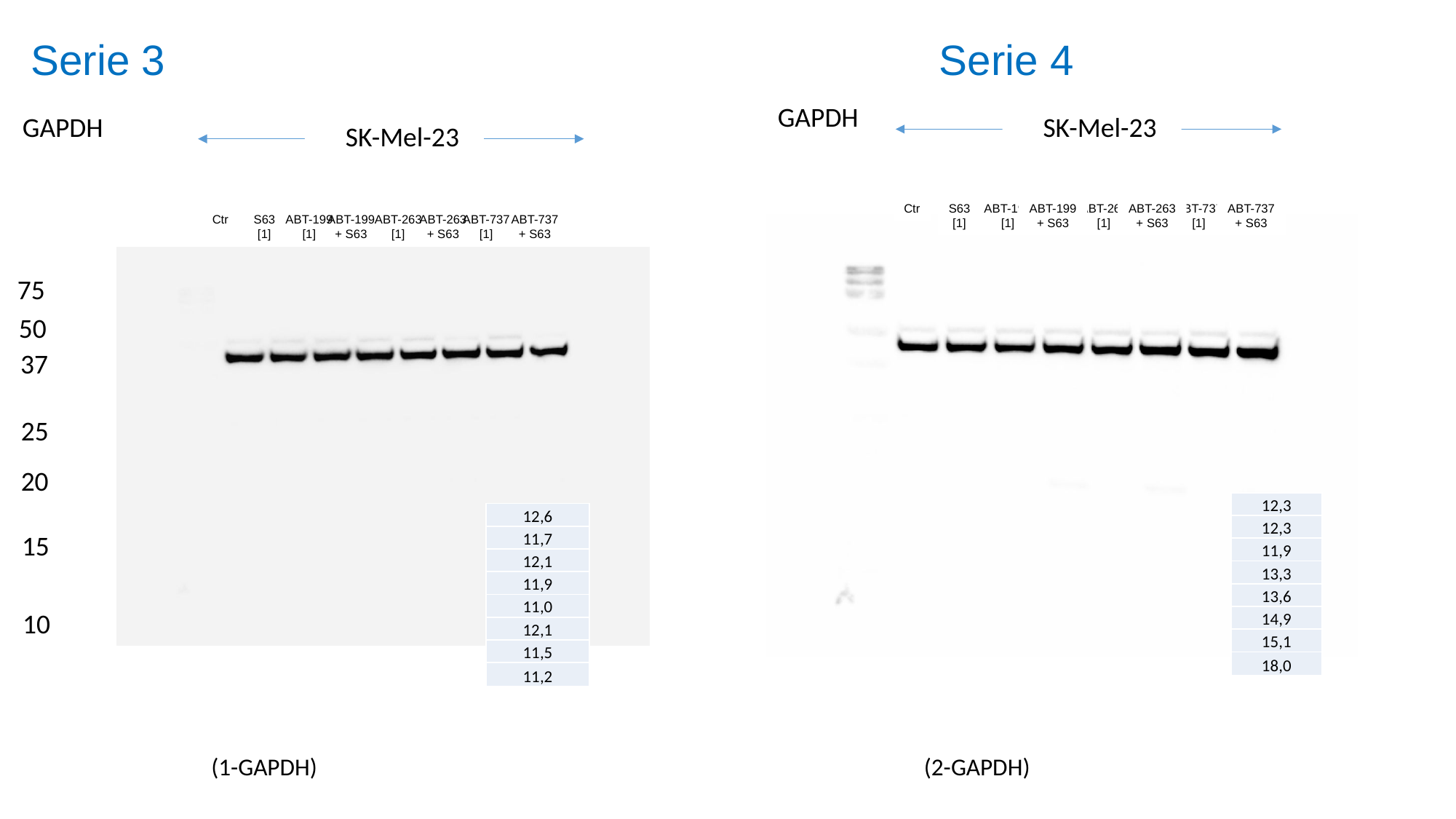

Serie 3
Serie 4
GAPDH
SK-Mel-23
GAPDH
SK-Mel-23
Ctr
S63
[1]
ABT-199
[1]
ABT-199
+ S63
ABT-263
[1]
ABT-263
+ S63
ABT-737
[1]
ABT-737
+ S63
Ctr
S63
[1]
ABT-199
[1]
ABT-199
+ S63
ABT-263
[1]
ABT-263
+ S63
ABT-737
[1]
ABT-737
+ S63
75
50
37
25
20
| 12,3 |
| --- |
| 12,3 |
| 11,9 |
| 13,3 |
| 13,6 |
| 14,9 |
| 15,1 |
| 18,0 |
| 12,6 |
| --- |
| 11,7 |
| 12,1 |
| 11,9 |
| 11,0 |
| 12,1 |
| 11,5 |
| 11,2 |
15
10
 (1-GAPDH)
 (2-GAPDH)
